# Vitamin C in Critically Ill Patients: An Updated Systematic Review and Meta-Analysis

**DOI:** 10.3390/nu13103564

**Published:** 2021-10-12

**Authors:** Dhan Bahadur Shrestha, Pravash Budhathoki, Yub Raj Sedhai, Sujit Kumar Mandal, Shreeja Shikhrakar, Saurab Karki, Ram Kaji Baniya, Markos G. Kashiouris, Xian Qiao, Alpha A. Fowler

**Affiliations:** 1 Department of Internal Medicine, Mount Sinai Hospital, Chicago, IL 60608, USA; 2 Department of Internal Medicine, Bronxcare Health System, Bronx, NY 10457, USA; pravash.budhathoki123@gmail.com; 3 Department of Internal Medicine, Division of Hospital Medicine, Virginia Commonwealth University, School of Medicine, Richmond, VA 23298, USA; 4 Department of Internal Medicine, Nepalese Army Institute of Health Sciences, Kathmandu 44600, Nepal; sujeetmandal4888@gmail.com; 5 Department of Internal Medicine, Kathmandu University School of Medical Sciences, Dhulikhel 45209, Nepal; shikhrakarshreeja@gmail.com; 6 Department of Internal Medicine, Military Hospital, Itahari 56705, Nepal; saurabkarki1010@gmail.com; 7 Department of Internal Medicine, Our Lady of the Lake Regional Medical Center, Baton Rouge, LA 70808, USA; rbaniya.md@gmail.com; 8 Department of Internal Medicine, Division of Pulmonary Disease and Critical Care, Virginia Commonwealth University, School of Medicine, Richmond, VA 23298, USA; alpha.fowler@vcuhealth.org; 9 Department of Internal Medicine, Division of Pulmonary Disease and Critical Care, Eastern Virginia Medical School, Norfolk, VA 23507, USA; xxqiao@sentara.com

**Keywords:** vitamins, ascorbic acid, critical illness, acute kidney injury, intensive care units, oxidative stress

## Abstract

Background: Vitamin C is a water-soluble antioxidant vitamin. Oxidative stress and its markers, along with inflammatory markers, are high during critical illness. Due to conflicting results of the published literature regarding the efficacy of vitamin C in critically ill patients, and especially the concerns for nephrotoxicity raised by some case reports, this meta-analysis was carried out to appraise the evidence and affirmation regarding the role of vitamin C in critically ill patients. Methods: We searched the database thoroughly to collect relevant studies that assessed intravenous vitamin C use in critically ill patients published until 25 February 2021. We included randomized controlled trials and observational studies with 20 or more critically ill patients who have received intravenous ascorbic acid (vitamin C). After screening 18,312 studies from different databases, 53 were included in our narrative synthesis, and 48 were included in the meta-analysis. We used the Covidence software for screening of the retrieved literature. Review Manager (RevMan) 5.4 was used for the pooling of data and Odds Ratios (OR) and Mean difference (MD) as measures of effects with a 95% confidence interval to assess for explanatory variables. Results: Pooling data from 33 studies for overall hospital mortality outcomes using a random-effect model showed a 19% reduction in odds of mortality among the vitamin C group (OR, 0.81; 95% CI, 0.66–0.98). Length of hospital stay (LOS), mortality at 28/30 days, ICU mortality, new-onset AKI and Renal Replacement Therapy (RRT) for AKI did not differ significantly across the two groups. Analysis of data from 30 studies reporting ICU stay disclosed 0.76 fewer ICU days in the vitamin C group than the placebo/standard of care (SOC) group (95% CI, −1.34 to −0.19). This significance for shortening ICU stay persisted even when considering RCTs only in the analysis (MD, −0.70; 95% CI, −1.39 to −0.02). Conclusion: Treatment of critically ill patients with intravenous vitamin C was relatively safe with no significant difference in adverse renal events and decreased in-hospital mortality. The use of vitamin C showed a significant reduction in the length of ICU stays in critically ill patients.

## 1. Introduction

Vitamin C (ascorbic acid) is a water-soluble essential vitamin synthesized by most plants and animals. Vitamin C and stress have long been associated, and previous research has shown that vitamin C reduces systemic inflammation, helps correct sepsis-induced coagulopathy, and prevents vascular injury [1,2,3]. Vitamin C has drawn substantial interest in critical care medicine, and several studies have been published, especially in the last five years, with intriguing but conflicting results. In a primary study by Fowler et al. [3] in 2014, vitamin C decreased the risk of organ failure in patients with a critical illness. However, the subsequent CITRIS-ALI trial failed to show benefit in organ dysfunction or suppress the inflammatory markers and vascular injury but reported a 28-day mortality benefit: 29.8% in the vitamin C group and 46.3% in the placebo group [4]. Marik et al. [5] showed a reduction in mortality and organ failure with vitamin C use among severe sepsis or septic shock patients. A study by Zabet et al. [6] in 2016 showed a reduction in the dose and duration of vasopressors medication use in surgical septic shock patients. A study by Nakajima et al. [7] in 2019 showed mortality benefit with high dose vitamin C use in patients with severe burn. Thus, vitamin C has been extensively studied in various critical care settings including medical, surgical, trauma and burn ICUs. Prior meta-analyses have shown a reduction in the duration of vasopressor and mechanical ventilation days [8,9], although a mortality benefit has not been seen [9,10,11,12]. Many studies have been published since the publication of the last meta-analyses by Wei et al. and Putzu et al. [10,11].

Thus, in order to fully appraise the available data, we sought to perform this systematic review and meta-analysis, including 53 studies with 352,395 critically ill patients, to evaluate the role of vitamin C, paying particular interest to the renal safety of vitamin C.

## 2. Methods

The protocol for this systematic review and meta-analysis was prepared and registered in PROSPERO, and the review was conducted according to the protocol’s predefined criteria (CRD42020222906) [13]. We abide by our study as per the standard Preferred Reporting Items for Systematic Reviews and Meta-Analysis (PRISMA) guidelines [14]. 

### 2.1. Search Strategy

We conducted a thorough literature search on PubMed, PubMed Central, Scopus and Embase to collect relevant studies published until 25 February 2021, reporting the use of intravenous vitamin C in critically ill patients. We used appropriate search words such as ascorbic acid, vitamin C, sepsis, septic shock, critical illness, intensive care unit, ICU, burn, ARDS and trauma using appropriate Boolean operators without any filters and language restrictions. Details of the electronic database search are available in Supplementary file S1. 

### 2.2. Selection Criteria

Inclusion criteria:

Type of studies: randomized controlled trials and other observational studies, namely, cohort studies, case–control studies, cross-sectional studies and case series with 20 or more critically ill patients.

Population: Adult patient with a critical illness.

Intervention: Intravenous vitamin C alone or in combination with other antioxidants. The comparator includes standard of care or placebo.

Study outcome: Hospital mortality, 28/30-day mortality, ICU mortality, length of stay in ICU and hospital, the adverse renal outcome in the form of new-onset AKI and AKI requiring renal replacement therapy were our main outcomes of interest. We used Odds Ratios (OR) and mean differences (MD) as a measure of effects to assess the explanatory variables.

Exclusion criteria:

Types of studies: Any form of review including meta-analysis, narrative reviews, case reports, opinions, editorials, letter to the editor with no primary data, study protocols, conference abstracts/presentations, thesis dissertation and animal studies.

Full-text articles that are not retrievable.

Incomplete studies (still ongoing) or preliminary reports with no complete data available.

COVID-19 related studies.

### 2.3. Data Extraction

We used Covidence software to screen the literature imported from databases for the eligibility assessment [15]. Every citation was reviewed independently by two reviewers (S.K. and S.S.) for eligibility, and conflicts were resolved by a third reviewer (S.M.). After two phases of screening (title and abstract and full-text review), the data from selected studies were extracted in a Microsoft Excel sheet. The number of manuscripts chosen was divided across the reviewers. Each reviewer curated the data from assigned articles, which was later verified for accuracy and any discrepancies by other reviewers. The internal review was carried out thoroughly to ensure the correctness of the data curation among the outcomes of interest with no subjective errors.

### 2.4. Risk of Bias

**Figure 1:**
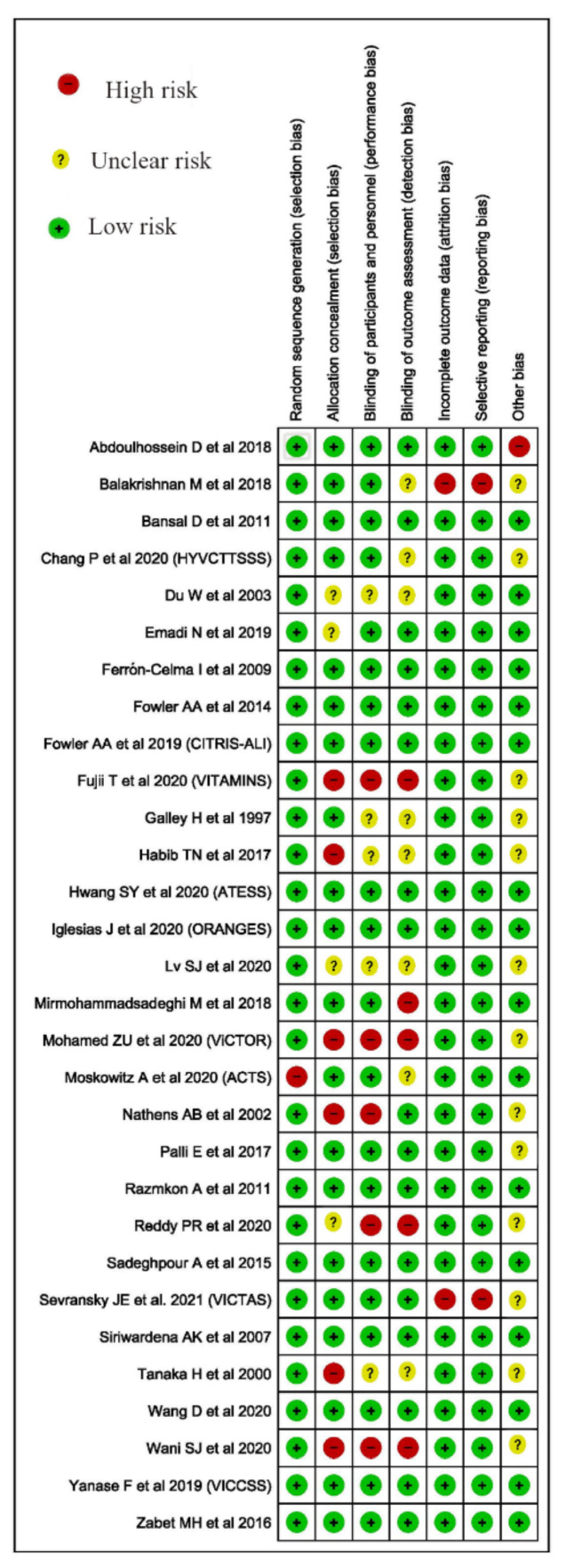
Cochrane ROB 2.0 of the included trials.

We used the Cochrane Risk of Bias (ROB) 2.0 tool for randomized control trials and Joanna Briggs Institute (JBI) critical appraisal checklist for observational studies for quality and risk of bias assessment (Figure 1 and Table 1) [16,17]. We used Review Manager (RevMan) 5.4 for creating a summary of biases for RCTs [18].

### 2.5. Statistical Analysis

Revman 5.4 was used to pool the data using Odds Ratio (OR) to estimate dichotomous outcome with a 95% Confident Interval (CI). Based on the level of heterogeneities, we used the fixed/random-effects model to pool the result. We used the mean differences (MD) between vitamin C and control groups for continuous variables. Where mean was not available in the study, mean and standard deviation (SD) were estimated using the sample size, median value and inter-quartile ranges [40].

### 2.6. Assessment of Heterogeneity

Based on the established recommendation for the statistical heterogeneity, we stratified heterogeneity as low, moderate and high to I^2^ values of 25, 50 and 75%, respectively [41]. Based on the calculated heterogeneity I^2^ test value, we used the random/fixed effect model for the analysis. We conducted sensitivity analysis by re-running the analysis to assess the hidden differences where appropriate.

## 3. Results

**Figure 2:**
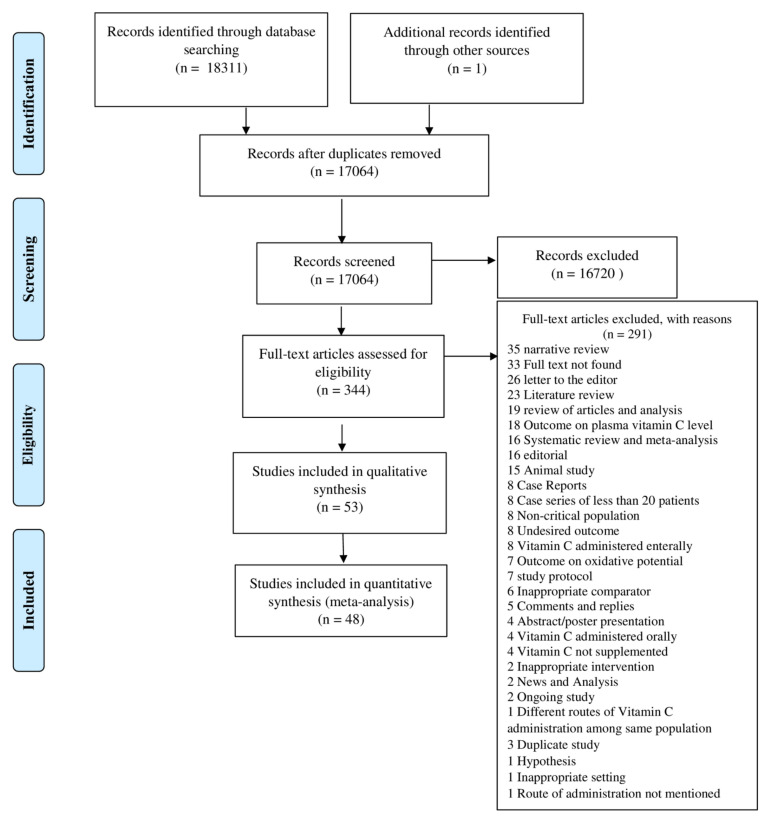
PRISMA Flow Diagram.

After electronic database searches, we retrieved 18,312 studies imported in Covidence, and 1248 duplicates were removed. After the duplicate check, we screened the 17,064 studies in a title and abstracts review, and we excluded 16,720 studies for not meeting the inclusion criteria. A total of 344 studies were filtered for full-text review, of which 291 studies were excluded for definite reasons. After a full-text review, we included 53 studies in the narrative review and 48 studies (27 randomized controlled trials and 21 observational studies) in the meta-analysis (Figure 2).

### 3.1. Narrative Summary

A total of 53 studies were included in the narrative review; of them, 23 were observational studies, while 30 were RCTs (Table 2). Basic study details are presented in Supplementary file S2.

### 3.2. Quantitative Synthesis

#### 3.2.1. Hospital Mortality

**Figure 3:**
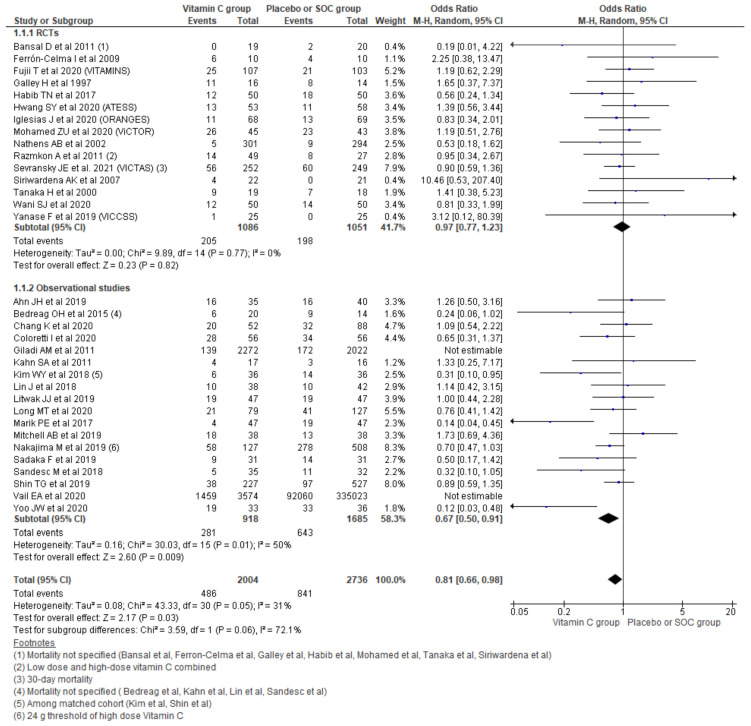
Forest plot showing overall hospital mortality using random-effect model.

Pooling the data on hospital mortality from the 33 studies using a random effect model showed a 19% reduction in odds for hospital mortality (OR, 0.81; 95% CI, 0.66–0.98; *n* = 4740; I^2^ = 31%; *p* = 0.03). Considering the type of study running subgroup analysis showed that a 33% reduction in mortality was reported among the observational studies (OR 0.67, 95% CI 0.50–0.91; *n* = 2603; I^2^ = 50%; *p* = 0.009). However, pooling the hospital mortality outcome among only 15 RCTs could not establish such a relation (OR 0.97, 95% CI 0.77–1.23; *n* = 2137; I^2^ = 0%; *p* = 0.82) (Figure 3).

#### Sensitivity Analysis among RCTs

To further analyze the hospital mortality outcome among RCTs, we re-run the analysis based on the subgroups of critically ill patients as surgical and sepsis/septic shock. Neither among patients with sepsis/septic shock (OR, 0.98; 95% CI, 0.76–1.26; *n* = 1297; I^2^ = 0%; *p* = 0.86) nor among surgical patients (OR, 1.08; 95% CI, 0.63–1.86; *n* = 860; I^2^ = 4%) was a significant difference for hospital mortality observed while comparing vitamin C and placebo or SOC groups (Supplementary file S3, Figure S1).

Based on the blinding of RCTs, the odds for mortality was shown to be slightly higher in double blinded trials among the vitamin C group (OR, 1.04; 95% CI, 0.76–1.43; *n* = 938; I^2^ = 0%; *p* = 0.80), while for single blinded/open, odds were slightly reduced (OR 0.92, 95% CI 0.65 to 1.29; *n* = 1199; I^2^ = 0%; *p* = 0.61) compared with the placebo or SOC group, but it could not reach statistical significance (Supplementary file S3, Figure S2).

Re-running analysis was conducted based on concomitant use of antioxidants to see any differences. Analysis comparing placebo/SOC with vitamin C only (OR, 0.93; 95% CI, 0.49–1.76; *n* = 207; I^2^ = 5%; *p* = 0.82) and placebo/SOC with other concomitant antioxidants use with vitamin C (OR, 0.99; 95% CI, 0.77–1.27; *n* = 1930; I^2^ = 0%; *p* = 0.94) also could not show significant differences in hospital mortality (Supplementary file S3, Figure S3).

#### Sensitivity Analysis among Observational Studies

In the sepsis/septic shock group, mortality could not reach significant differences (OR, 0.73; 95% CI, 0.46–1.15; *n* = 340,351; I^2^ = 86%; *p* = 0.17), while among surgical patients (OR, 0.69; 95% CI, 0.56–0.85; *n* = 5143; I^2^ = 4%; *p* = 0.0005), a significant reduction in hospital mortality was observed comparing the vitamin C with placebo/SOC groups (Supplementary file S3, Figure S4).

The analysis comparing placebo/SOC with vitamin C only could not reach significant differences in mortality (OR, 0.86; 95% CI, 0.64–1.16; *n* = 963; I^2^ = 0%; *p* = 0.32), while comparing placebo/SOC with other concomitant antioxidants use with vitamin C showed a significant reduction in hospital mortality (OR, 0.60; 95% CI, 0.38–0.95; *n* = 344,531; I^2^ = 91%; *p* < 0.0001) (Supplementary file S3, Figure S5).

#### 3.2.2. 28/30-Day Mortality

**Figure 4:**
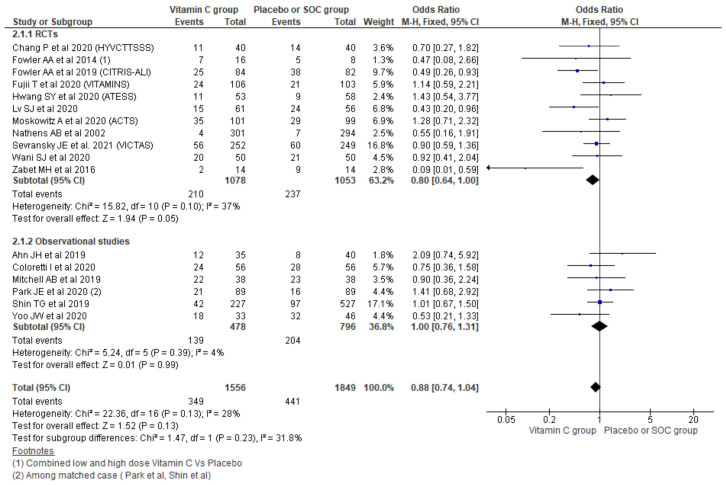
Forest plot showing 28-days mortality using fixed-effect model.

Using a fixed effect model effect, the pooling of data from 17 studies showed a 0.88 odds of 28/30-day mortality among the vitamin C group compared with placebo/SOC (95% CI, 0.74–1.04; *n* = 3405; I^2^ = 28%; *p* = 0.13), which could not reach statistical significance. Further, the subgroup analysis based on the type of studies showed a 0.80 odds of 28/30-day mortality among the vitamin C group among RCTs (OR, 0.80; 95% CI, 0.64–1.00; *n* = 2131; I^2^ = 37%; *p* = 0.05), which also did not differ significantly with placebo/SOC. Similarly, taking observational studies only also showed no significant differences across the two groups (OR, 1.00; 95% CI, 0.76–1.31; *n* = 1274; I^2^ = 4%; *p* = 0.99) (Figure 4).

Considering mild–moderate heterogeneity and pooling the results using random-effect model also showed no significant differences (Supplementary file S3, Figure S6).

#### 3.2.3. ICU Mortality

Pooling data from 14 studies reporting ICU mortality showed an OR of 0.83 for overall ICU mortality (95% CI, 0.61–1.13; *n* = 2448; I^2^ = 38%; *p* = 0.24), though it could not reach statistical significance. Similarly, further subgroup analysis taking the type of study under consideration showed a similar result with RCTs (OR, 1.02; 95% CI, 0.75–1.38; *n* = 1716; I^2^ = 0%; *p* = 0.91) and observational studies (OR, 0.69; 95% CI, 0.41–1.17; *n* = 732; I^2^ = 55%; *p* = 0.17) (Supplementary file S3, Figure S7).

#### 3.2.4. Length of Hospital Stay

**Figure 5:**
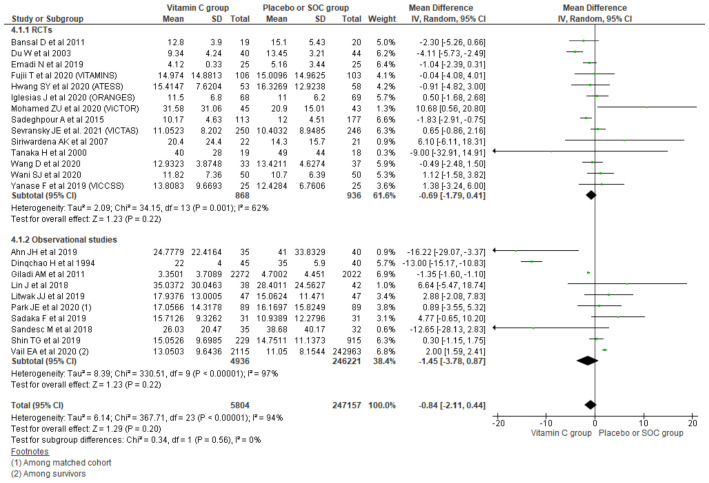
Forest plot showing mean difference of LoHS using random-effect model.

Pooling data from 24 studies reporting the length of hospital stay (LoHS) outcome using a random effect model showed a mean difference of -0.84 days comparing the vitamin C group with placebo/SOC (95% CI, −2.11 to 0.44; *n* = 252,961; I^2^ = 94%; *p* = 0.20). Similarly, further subgrouping of the LoHS outcome based on the type of study also could not reach the level of significance for reduction in LoHS among the vitamin C group compared with placebo/SOC among RCTs (MD, −0.69; 95% CI, 1.79 to 0.41; *n* = 1804; I^2^ = 62%; *p* = 0.22) and observational studies (MD, −1.45; 95% CI, −3.78 to 0.87; *n* = 251,157; I^2^ = 97%; *p* = 0.22) (Figure 5).

##### Sensitivity Analysis among RCTs

Among the patients in sepsis/septic shock, there were no significant differences in the length of hospital stay between the two groups (MD, 0.63; 95% CI, −0.41 to 1.68; *n* = 1141; I^2^ = 0%; *p* = 0.23); however, among critically ill surgical patients, an average 1.7 days reduction in LoHS was observed among the vitamin C group compared with placebo/SOC (MD, −1.70; 95% CI, −2.89 to −0.51; *n* = 663; I^2^ = 52%; *p* = 0.005) (Supplementary file S3, Figure S8).

Running analysis for LoHS taking RCTs based on the blinding of trials showed a significant reduction in the length of hospital stay of the vitamin C group among both double blinded trials (MD, −0.54; 95% CI, −1.43 to 0.36; *n* = 1247; I^2^ = 34%; *p* = 0.24), and the single blinded/open RCTs’ reduction in LoHS among the vitamin C group could not reach statistical significance (MD, −0.78; 95% CI, −3.77 to 2.20; *n* = 557; I^2^ = 73%; *p* = 0.61) compared with the placebo or SOC group (Supplementary file S3, Figure S9).

Analysis comparing vitamin C only with placebo/SOC showed on average a 1.7 day reduction in LoHS among the vitamin C group (MD, −1.70; 95% CI, −3.02 to −0.37; *n* = 581; I^2^ = 61%; *p* = 0.01), while such reduction is not seen in other concomitant antioxidants use with vitamin C (MD, 0.31; 95% CI, −0.91 to 1.53; *n* = 1223; I^2^ = 21%; *p* = 0.62) and also could not show significant differences in hospital mortality (Supplementary file S3, Figure S10).

##### Sensitivity Analysis among Observational Studies

In both the sepsis/septic shock group (MD, 1.34; 95% CI, −0.43 to 3.10; *n* = 246,631; I^2^ = 64%; *p* = 0.14) and the surgical group (MD, −5.25; 95% CI, −14.19 to 3.69; *n* = 4526; I^2^ = 97%; *p* = 0.25), the reduction in LoHS could not reach statistical significance comparing the vitamin C with the placebo/SOC groups (Supplementary file S3, Figure S11).

Running analysis considering the concomitant use of other antioxidants with vitamin C among observational studies also could not show significant differences in the length of hospital stay comparing placebo/SOC with vitamin C only (MD, −8.12; 95% CI, −19.97 to 3.74; *n* = 240; I^2^ = 80%; *p* = 0.18) or the concomitant use of other antioxidants with vitamin C (MD, 0.83; 95% CI, −1.31 to 2.97; *n* = 250,917; I^2^ = 97%; *p* = 0.45) (Supplementary file S3, Figure S12).

#### 3.2.5. Length of ICU Stay

**Figure 6:**
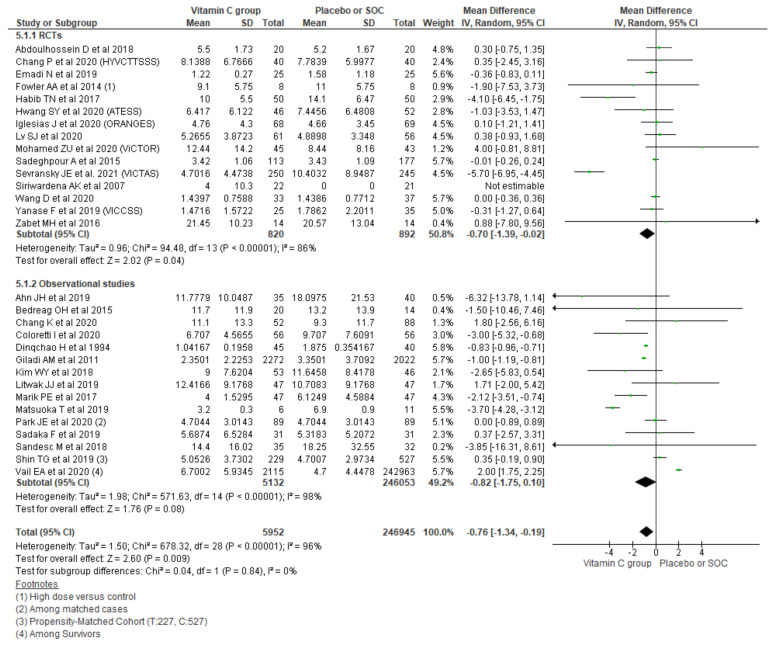
Forest plot showing mean difference of length of ICU stays using a random-effect model.

Polling data from 30 studies reporting the length of ICU stay outcome using a random effect model showed an overall mean difference of −0.76 days, indicating a 0.76-day shorter ICU stay among the vitamin C group compared with placebo/SOC (95% CI, 95% CI, −1.34 to −0.19; *n* = 252,897; I^2^ = 96%; *p* = 0.009). Similarly, further subgrouping of the length of ICU stay based on the type of study also showed a small but significant reduction in the length of ICU stay among the vitamin C group comparing with placebo/SOC among RCTs (MD, −0.70; 95% CI, −1.39 to −0.02; *n* = 1712; I^2^ = 86%; *p* = 0.04), while the observational studies could not reach the level of significance (MD, −0.82; 95% CI, −1.75 to 0.10; *n* = 251,185; I^2^ = 98%; *p* = 0.08) (Figure 6).

#### 3.2.6. New-Onset Acute Kidney Injury (AKI)

**Figure 7:**
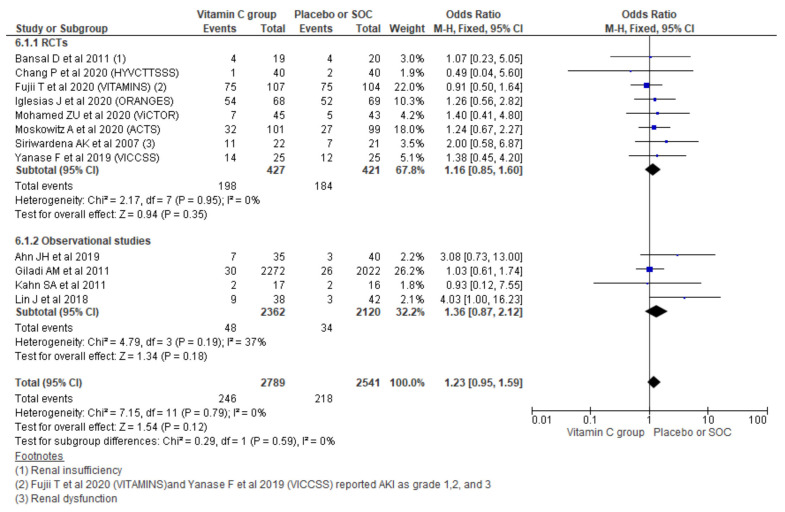
Forest plot showing the new occurrence of AKI using a fixed-effect model.

Pooling new onset AKI outcomes using a fixed effect model from 12 studies showed no significant increment in new AKI (OR, 1.23; 95% CI, 0.95 to 1.59; *n* = 5330; I^2^ = 0%; *p* = 0.12). Similarly, considering the type of study also could not show a significant increment in new AKI among observational studies (OR, 1.36; 95% CI, 0.87 to 2.12; *n* = 4482; I^2^ = 37%; *p* = 0.18) and RCTs (OR, 1.16; 95% CI, 0.85 to 1.60; *n* = 848; I^2^ = 0%; *p* = 0.35) (Figure 7).

Pooling the new onset AKI outcomes using a random-effect model also showed no significant difference in new AKI outcomes (OR, 1.22; 95% CI, 0.94 to 1.58; *n* = 5330; I^2^ = 0%; *p* = 0.14). Similarly, the type of study could not show significant changes among observational studies and RCTs (Supplementary file S3, Figure S13).

#### 3.2.7. Renal Replacement Therapy (RRT) for AKI

**Figure 8:**
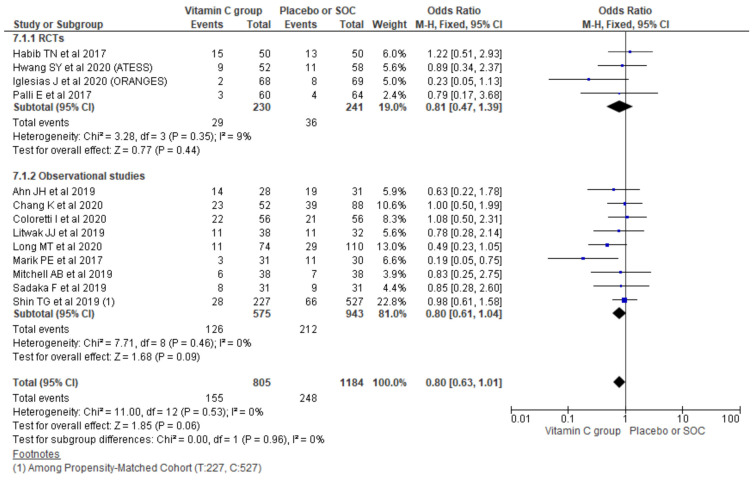
Forest plot showing requirement of RRT for AKI using a random-effect model.

The pooling requirement of RRT for AKI using a random-effect model from 13 studies showed no significant differences in the overall requirement of RRT for AKI (OR, 0.80; 95% CI, 0.63 to 1.01; *n* = 1989; I^2^ = 0%; *p* = 0.06). Similarly, considering the type of study also could not show a significant difference among the two treatment groups in observational studies (OR, 0.80; 95% CI, 0.61 to 1.04; *n* = 1518; I^2^ = 0%; *p* = 0.09) and RCTs (OR, 0.81; 95% CI, 0.47 to 1.39; *n* = 471; I^2^ = 9%; *p* = 0.44) (Figure 8).

## 4. Discussion

Herein, we report a comprehensive systematic review and meta-analysis including 48 previously published studies. We have included 27 randomized controlled trials and 21 observational studies that have studied the role of intravenous vitamin C in critically ill patients. We found a reduction in overall hospital mortality among critically ill patients who received vitamin C, in contrast to the findings of an earlier meta-analyses by Langlois et al. and Wei et al. [11,12]. However, this finding could have been impacted by the inclusion of observational studies in the cumulative analysis. These findings could not be replicated when a subgroup analysis was performed for the randomized controlled trials alone. Mortality benefit was observed when a cumulative analysis was performed for the observational studies alone. 

Oxidative stress is thought to play a central role in the pathophysiology of the inflammatory syndrome post-operative state after surgery and sepsis. Vitamin C deficiency is commonly seen in surgical patients and patients in intensive care units [43,71]. The intense inflammatory process leads to an increased turnover of vitamin C, further resulting in severe deficiency [43,71]. Parenteral vitamin C has been investigated for its potential anti-inflammatory, antioxidant effects [3]. Vitamin C has also been studied for its properties to improve immune function, regulate microcirculation and prevent thrombosis [3]. Although the therapeutic efficacy of vitamin C is seen in individual studies, we found no difference in mortality among patients receiving intravenous vitamin C while performing a subgroup analysis of RCTs including surgical patients or patients with sepsis/septic shock. A reduction in mortality was observed among surgical patients in observational studies. We found no difference in the length of hospital stay in the analysis of the cohort and subgroup analyses. A reduction in the length of ICU stay was observed while analyzing studies reporting ICU stay and also among RCTs only separately, although a level of statistical significance could not be achieved while analyzing observational studies separately. Prior meta-analyses conducted by Langlois et al., Putzu et al. and Wei et al. found no difference in the duration of hospital stay and the length of ICU stay [10,11,12]. However, in a subgroup analysis, Putzu et al. found decreased lengths of hospital and ICU stays among patients undergoing cardiac surgery [10]. Vitamin C has shown to decrease the risk of postoperative atrial fibrillation in patients undergoing cardiac surgery, a further benefit observed in terms of the deceased length of ICU stay suggests that vitamin C may have beneficial effects from mechanisms yet to be recognized [72,73].

Vitamin C is metabolized into oxalate compounds that can accumulate in the kidneys and trigger acute kidney injury [44]. In our meta-analysis, we found no increase in the new-onset acute kidney injury or requirement for renal replacement therapy among the patients receiving vitamin C. This finding is consistent with the results of a prior meta-analyses conducted by Putzu et al. and Wei et al. [10,11]. Collectively, this suggests that vitamin C is potentially more renally safe than perceived.

We have sought to perform the most comprehensive meta-analysis, including 48 studies in quantitative analysis evaluating the vitamin C use in critically ill patients and their clinical outcomes. We have only included studies in which vitamin C was administered parenterally. High dose oral vitamin C saturates the intestinal absorption mechanism; thus, a higher plasma concentration cannot be achieved by a higher oral dose administration. We assumed that the intravenous administration makes it possible to achieve the higher plasma concentration required to scavenge oxidizing radicals and protect from their deleterious effects. Prior meta-analyses conducted in the role of vitamin C among critically ill patients have included fewer studies than our analysis [10,11,12]. They have included studies using both oral and parenteral formulations of vitamin C, which adds to the strength of our study [10,11,12]. Our analysis is further strengthened by the subgroup analyses conducted based on the clinical settings, medical and surgical etiology of hospitalization, sepsis and septic shock, and the type of studies.

Our study has several limitations. The included RCTs and observational studies have their own inherent limitations. The baseline characteristics of the population studied and the assessment of organ dysfunction were not individually reported across the studies. The formulations, dosing and duration of vitamin C use are comparable and offer granularity of data in assessing individual influence. Further, the patients could have been enrolled in different stages, and the severity of sepsis and ARDS, and clinical outcomes could have been influenced by the stage of the pathology.

ICU pathophysiology is complex and clinical outcomes are determined several factors relating to patients’ general health and acute physiological state. This includes factors that cannot be reversed by vitamin C therapy alone and results can be confounded. Various studies in our analysis included treatment with other antioxidants such as thiamine and steroids, which can act as potential confounders. Nevertheless, our observation of no significant difference across the two groups in acute kidney injury and need for renal replacement therapy is reassuring in terms of the renal safety of intravenous vitamin C therapy and can guide future investigators.

## 5. Conclusions

The treatment of critically ill patients with vitamin C did not offer mortality benefits in terms of a reduction in in-hospital mortality, 28/30-day mortality and ICU mortality based on the cumulative analysis of the randomized controlled trials. However, vitamin C did reduce the length of hospitalization, although it did not reduce the number of ICU days. In addition, vitamin C therapy was relatively safe, especially from a renal standpoint, given the no significant group difference in the incidence of acute kidney injury and the need for renal replacement therapy.

## Figures and Tables

**Table 1:** JBI Bias Assessment.

Questionnaires	Were the Two Groups Similar and Recruited from the Same Population?	Were the Exposures Measured Similarly to Assign People to Both Exposed and Unexposed Groups?	Was the Exposure Measured in a Valid and Reliable Way?	Were Confounding Factors Identified?	Were Strategies to Deal with Confounding Factors Stated?	Were the Groups/Participants Free of the Outcome at the Start of the Study (or at the Moment of Exposure)?	Were the Outcomes Measured in a Valid and Reliable Way?	Was the Follow Up Time Reported and Sufficient to Be Long Enough for Outcomes to Occur?	Was Follow Up Complete, and If Not, Were the Reasons to Loss to Follow Up Described and Explored?	Were Strategies to Address Incomplete Follow Up Utilized?	Was Appropriate Statistical Analysis Used?	Overall Appraisal
Ahn et al., 2019 [19]	Yes	Yes	Yes	No	No	Yes	Yes	Yes	Yes	NA	Yes	Include
Bedreag et al., 2015 [20]	Yes	Yes	Yes	No	NA	Yes	Yes	Yes	Yes	NA	Yes	Include
Chang et al., 2020 [21]	Yes	Yes	Yes	Yes	No	Yes	Yes	Yes	Yes	NA	Yes	Include
Coloretti et al., 2020 [22]	Yes	Yes	Yes	Yes	Yes	Yes	Yes	Yes	Yes	NA	Yes	Include
Dingchao et al., 1994 [23]	Yes	Yes	Yes	No	NA	Yes	Yes	Yes	Yes	NA	Yes	Include
Giladi et al., 2011 [24]	Yes	Yes	No	Yes	No	Yes	Yes	Yes	Yes	NA	Yes	Include
Kahn et al., 2011 [25]	Yes	Yes	Yes	Yes	Yes	Yes	Yes	Yes	Yes	NA	Yes	Include
Kim et al., 2018 [26]	Yes	Yes	Yes	Yes	Yes	Yes	Yes	Yes	Yes	NA	Yes	Include
Lin et al., 2017 [27]	Yes	Yes	Yes	Yes	Yes	Yes	Yes	Yes	Yes	NA	Yes	Include
Litwak et al., 2019 [28]	Yes	Yes	Yes	No	No	Yes	Yes	Yes	Yes	NA	Yes	Include
Long et al., 2020 [29]	Yes	Yes	Yes	Yes	No	Yes	Yes	Yes	Yes	NA	Yes	Include
Marik et al., 2017 [5]	Yes	Yes	Yes	Yes	Yes	Yes	Yes	Yes	NA	NA	Yes	Include
Masood et al., 2019 [30]	NA	NA	Yes	No	No	Yes	Yes	Yes	Yes	Yes	Yes	Include
Matsuoka et al., 2020 [31]	Yes	Yes	Yes	No	No	Yes	Yes	Yes	Yes	NA	Yes	Include
Mitchell et al., 2020 [32]	No	Yes	Yes	Yes	No	Yes	Yes	Yes	Yes	NA	Yes	Include
Nagel et al., 2020 [33]	Yes	Yes	Yes	Yes	No	Yes	Yes	Yes	Yes	NA	Yes	Include
Nakajima et al., 2019 [7]	Yes	Yes	Yes	Yes	Yes	Yes	Yes	Yes	Yes	NA	Yes	Include
Park et al., 2020 [34]	Yes	Yes	Yes	Yes	Yes	Yes	Yes	Yes	Yes	NA	Yes	Include
Sadaka et al., 2020 [35]	Yes	Yes	Yes	Yes	Yes	Yes	Yes	Yes	Yes	NA	Yes	Include
Sandesc et al., 2018 [36]	Yes	Yes	Yes	No	No	Yes	Yes	Yes	Yes	NA	Yes	Include
Shin et al., 2019 [37]	Yes	Yes	Yes	Yes	Yes	Yes	Yes	Yes	Yes	NA	Yes	Include
Vail et al., 2020 [38]	Yes	Yes	Yes	Yes	Yes	Yes	Yes	Yes	Yes	NA	Yes	Include
Yoo et al., 2020 [39]	Yes	Yes	Yes	Yes	Yes	Yes	Yes	Yes	Yes	NA	Yes	Include

**Table 2:** Narrative summary of included studies.

ID	Population	Intervention	Comparisons	Outcome
Abdoulhossein et al., 2018 [42]	N = 40 [T = 20, C = 20] Age (mean ± SD): T = 40.35 ± 8.94, C = 39.95 ± 9.05 Gender, *n* (%); Male: T = 14(70%), C = 12(60%) GCS, *n* (%) 13: T = 1 (5%) C = 1(5%) 14: T = 6 (30%) C = 5 (25%) 15: T = 13 (65%) C = 14 (70%)	Intravenous infusion vitamin C (500 mg in 50 mL normal saline) was administered by pump, steady, for up to 30 min along with standard of care.	The control group received intravenous infusion distilled water (5 mL in 50 mL of normal saline), by pump, steady, for up to 30 min.	ICU stay after intervention, days T = 5.5 ± 1.73 C = 5.2 ± 1.67
Ahn et al., 2019 [19]	N = 75 T = 35 C = 40 Age, mean ± SD (years) T = 67.8 ± 12.1 C = 63.6 ± 15.3 Sex, male, *n* [%] T = 24 [43] C = 29 [44] APACHE II score T = 25.2 ± 6.1 C = 24.1 ± 6.0 Day 1 SOFA score T = 10.3 ± 3.3 C = 11.3 ± 3.1 Vasopressors, *n* [%] T = 33/35 [94] C = 40/40 [100] Hydrocortisone, *n* [%] T = 16/35 [45] C = 27/40 [46] Creatinine, mean ± SD, mg/dL T = 1.74 ± 1.81 C = 2.15 ± 1.81AKI, *n* [%] T = 21/35 [47] C = 28/40 [74]	A total of 2 g of vitamin C mixed in 50 mL of 5% dextrose solution or normal saline was administered intravenously over 30 min every 8 h until ICU discharge.	Treatment protocol based on the Surviving Sepsis Guidelines. Patients could receive hydrocortisone to maintain blood pressure if required.	28-day mortality, *n* [%] T = 12/35 [34] C = 8/40 [20] 90-day mortality, *n* [%] T = 21/35 [48] C = 19/40 [49] ICU mortality, *n* [%] T = 12/35 [34] C = 12/40 [30] Hospital mortality, *n* [%] T = 16/35 [45] C = 16/40 [40] Shock reversal, days T = 3 [2,3,4,5] C = 3 [2,3,4,5] Mechanical ventilation, days T = 8 [5,6,7,8,9,10,11,12,13,14,15,16,17] C = 8 [4,5,6,7,8,9,10,11,12,13,14,15,16,17,18,19] ICU LOS, days T = 10 [6,7,8,9,10,11,12,13,14,15,16,17,18,19] C = 11 [7,8,9,10,11,12,13,14,15,16,17,18,19,20,21,22,23,24,25,26,27,28,29,30,31,32,33,34,35] Hospital LOS, days T = 23 [11,12,13,14,15,16,17,18,19,20,21,22,23,24,25,26,27,28,29,30,31,32,33,34,35,36,37,38,39,40] C = 41 [19,20,21,22,23,24,25,26,27,28,29,30,31,32,33,34,35,36,37,38,39,40,41,42,45,47,48,49,50,51,52,53,54,55,56,57,58,59,60,61,62,63,64,65,66] ΔSOFA score, first 4 ICU days T = −1.4 ± 3.3 C = −1.4 ± 3 RRT for AKI, *n* [%] T = 14/28 [55] C = 19/31 [65] Recovery from AKI, T = 13/28 [45] C = 16/31 [57] Total AKI, *n* T = 28/35 C = 31/40 New AKI during treatment: T = 7/35 C = 3/40
Balakrishnan et al., 2018 [50]	N = 24 [T = 12, C = 12] Age, mean: T = 55.41 ± 15.70; C = 53.41 ± 8.27 Male, *n* (%) T = 8(66.6), C = 7(58.3) Euroscore II T = 1.95 ± 1.29 C = 2.78 ± 4.06 APACHE IV Score T = 60.91 ± 17.52 C = 51.81 ±10.76	Intravenous vitamin C (1.5 g every 6 h for 4 days), hydrocortisone (50 mg every 6 h for 4 days) as well as intravenous thiamine (200 mg every 12 h for 4 days). The vitamin C was administered as an infusion over 30–60 min and mixed in a 50-milliliter solution of either 5% dextrose in water (D5W) or normal saline.	Received saline in a black syringe.	Urea (mg/dL) Day 1: T = 76.12 ± 47.18; C = 75.29 ± 46.96 Day 2: T = 79.85 ± 59.84; C = 71.03 ± 50.76 Day 3: T = 90.61 ± 66.78; C = 89.64 ± 59.73 Day 4: T = 89.58 ± 56.55; C = 93.43 ± 52.48 Creatinine (mg/dL) Day 1: T = 2.73 ± 2.16; C = 1.52 ± 0.79 Day 2: T = 2.17 ± 1.33; C = 1.43 ± 0.81 Day 3: T = 2.24 ± 1.33; C = 1.59 ± 0.75 Day 4: T = 2.25 ± 1.46; C = 1.68 ± 0.84
Bedreag et al., 2015 [20]	N = 34 [T = 20 C = 14] Gender, % male; T = 75 C = 85.71 Age, mean (SD), years T = 52 (14.5) C = 53.8 (20.9) APACHE II, mean (SD) T = 10.7 (5.8) C = 10.8 (7.6) ISS, mean (SD) T = 26.1 (8,4) C = 31.1 (9.4) GCS < 7, *n* (%) T = 1 (5) C = 6 (42.8) Urea (mg/dL), T = 59.5 (10.5) C = 64.1 (3.1)	Antioxidant therapy consisted of the administration of vitamin C, vitamin B1 and N-acetylcysteine (NAC).All patients received vitamin C (i.v): 1 g three times daily, for 5 days; vitamin B1 (i.v.): 100 mg daily for 3 days; and N-Acetylcysteine (i.v.): 300 mg twice a day, for the whole period of the ICU stay.	Standard of care.	Incidence of sepsis T = 8/20 C = 9/14 LOS in ICU T = 11.7 ± 11.9, C = 13.2 ± 13.9 Mortality T = 6/20 (30%) C = 9/14 (64.2%) Urea (mg/dL), mean (SD) at discharge from ICU T:27.8 (10.5); C: 57.1 (20.8)
Chang K et al., 2020 [21]	N = 140 T = 52 C = 88 Age, mean (SD) T = 63.9 (15.7) C = 63.6 (14.5) Female, *n* (%) T = 21 (40) C = 34 (39) APACHE IV, T = 95.1 (32.3) C = 86.1 (28.4) SOFA, T = 9.9 (3.8) C = 8.6 (3.1) Vasopressors, *n* (%) T = 51 (98) C = 85 (97)	Vitamin C at a dose of at least 1500 mg every six hours for up to 96 h.	Patients were treated following the Surviving Sepsis Campaign guidelines.	Hospital death, *n* (%) T = 20/52(39) C = 32/88 (36) ICU death, *n* (%) T = 16/52 (31) C = 20/88 (23) Ventilator-free days, T = 16.0 (11.8) C = 17.8 (11.8) Vasopressor-free days, T = 16.9 (11.8) C = 19.7 (11.5) ICU LOS, mean (SD) T = 11.1 (13.3) C = 9.3 (11.7) Dialysis, *n* (%) T = 23/52 (44) C = 39/88 (44) Vasopressors, *n* (%) T = 51 (98) C = 85 (97)
Chang P et al., 2020 [51]	N = 80 T = 40 C = 40 Age, y T = 59.5 ± 15.0 C = 63.7 ± 12.8 Sex, male T = 22 (57.5) C = 21 (52.5) Mechanical ventilation T = 30 (75) C = 32 (80) Vasopressors T = 22 (55) C = 24 (60) SOFA score T = 9.6 ± 4.5 C = 10.1 ± 4.0 APACHE II score T = 22.1 ± 8.4 C = 23.8 ± 7.6 CRF *n* (%) T = 4/40 (10) C = 5/40 (12.5) AKI *n* (%) T = 17/40 (42.5) C = 21/40 (52.5)	IV hydrocortisone (50 mg every 6 h for 7 days or until ICU discharge, whichever occurred first), vitamin C (1.5 g every 6 h for 4 days or until ICU discharge, whichever occurred first), and IV thiamine (200 mg every 12 h for 4 days or until ICU discharge, whichever occurred first)	Same frequency and volume of saline.	28-d mortality T = 11/40 (27.5) C = 14/40 (35) ICU LOS, d T = 7.5 (4–12.8) C = 7.5 (4–11.8) Duration of vasopressors, h T = 46 (23.8–102.5) C = 58.5 (28–104) New AKI after entering ICU T = 1/40 (2.5) C = 2/40 (5) Change in SOFA score, 72 h T = 3.5 ± 3.3 C = 1.8 ± 3.0 Duration mechanical ventilation, h T = 126.5 (63.5–239.3) C = 94.5 (39.8–211) Lactate clearance, 72 h, % T = 21.3 (−49.7 to 44.2) C = 0 (−35.1 to 47.7)
Coloretti et al., 2020 [22]	N = 112 T = 56 C = 56 Age (years) T = 69 (56–76) C = 69 (56–77) Male n(%) T = 37 (66.1) C = 32 (57.1) SAPS II score T = 61 (47–72) C = 56 (47–71) SOFA score T = 11 (8–12) C = 11 (8–13) Medical admission n(%) T = 36 (64.3) C = 33 (58.9)	Use of intravenous vitamin C at the dosage of 1.5 g every 6 h and thiamine at the dosage of 200 mg every 12 h up to discontinuation of vasoactive drugs or steroids.	Current sepsis management guidelines.	30-day mortality *n* (%) T = 24/56 (42.8) C = 28/56 (50.0) Hospital mortality *n* (%) T = 28/56(50.0) C = 34/56 (60.7); Mechanical ventilation: T: 41/56; C: 48/56 Vasopressors length (days) T = 3 (2–5) C = 4 (2–7) Mechanical ventilation (days) T = 3 (0–8) C = 6 (2–15) RRT n(%) T = 22/56 (39.2) C = 21/56 (37.5) ICU LOS (days) T = 6 (4–10) C = 9 (5–15)
Dingchao et al., 1994 [23]	N = 85 [T = 45 C = 40] There were no significant differences in age, weight, CPB time and aortic clamping time between the two groups.	Receiving 250 mg/kg of ascorbic acid. Ascorbic acid was introduced intravenously 30 min before CPB and at the aortic decamping (125 mg/kg each time).	Standard of care without ascorbic acid.	ICU stay (hour) T = 25 ± 4.7 C = 45 ± 8.5 Hospital stay (day) T = 22 ± 4.0 C = 35 ± 5.9
Du et al., 2003 [52]	N = 84 [ T = 40, C = 44] Age, mean years T = 40 ± 12 C = 43 ± 14 Gender (M/F) T= 25/15 C = 28/16	In the treatment group, vitamin C (10 g/day) was given intravenously for 5 days, high dose.	In the control group (*n* = 44), vitamin C (1 g/day) was given intravenously for 5 days (Low dose).	Hospital LOS T = 9.34 ± 4.24 C = 13.45 ± 3.21
Emadi et al., 2019 [45]	N = 50 [T = 25 C = 25] Age (years) T = 60 ± 6.62 C = 63.64 ± 8.26 Gender (male/female) (%) T = 72/28 C = 56/44	Received 5 g of intravenous vitamin C before induction of anesthesia and 5 g of vitamin C in the cardioplegic solution.	Given the same amount of placebo (normal saline).	Intubation time (h) T = 10.76 ± 2.14 C = 11.72 ± 2.57 ICU stay (days) T = 1.22 ± 0.27 C = 1.58 ± 1.18 Hospital stay (days) T = 4.12 ± 0.33 C = 5.16 ± 3.44
Fowler et al., 2014 [3]	N = 26 [TH = 8, TL = 8, C = 8] Gender(M/F): TH = 4/4 TL = 5/3 C = 4/4 Age, range in years TH = 49–92 TL = 30–70 C = 54–68 APACHE II score, mean (range). TH = 24.0 (12–33) TL = 20.4 (12–23) C = 20.4 (15–29) SOFA score, mean ± SE. TH = 10.8 ± 4.4 TL = 10.1 ± 2.0 C = 13.3 ± 2.9	Low dose ascorbic acid (Lo-AscA): TL 50 mg/kg/24 h High dose ascorbic acid (Hi-AscA) TH: 200 mg/kg/24 h. Ascorbic acid dosage was divided into 4 equal doses and administered over 30 min every 6 h for 96 h in 50 mL of 5% dextrose and water. Study drug infusion was initiated 2–4 h following informed consent and randomization.	Placebo: 5% dextrose and water.	Days on Vasopressor, mean (range) TH = 3.6 (2–8); TL = 2.1 (1–6); C = 3.9 (1–10) Ventilator Free Days, mean (range) TH = 4.8 (0–19); TL = 8.4 (0–22); C = 7.6 (0–23) ICU Length of Stay, mean (range) TH = 9.1 (2–25); TL = 8.1 (1–19); C = 11.0 (2– 25) 28-Day mortality, TH = 4/8 (50.6%); TL = 3/8 (38.1%); C = 5/8 (62.5%)
Fowler et al., 2019 [4]	N = 167 [T = 84 C = 83] Age, median (IQR), y T = 54 (39–67) C = 57 (44–70) Gender, number (%) Men T = 45 (54) C = 45 (54) Women T = 39 (46) C = 38 (46) Incidence of shock, No. (%) At baseline, vasopressor in use T = 57 (68) C = 60 (72) SOFA scores, mean (SD) At randomization T = 9.8 (3.2) C = 10.3 (3.1) At 96 h T = 8.02 (4.2) C = 6.96 (3.5) Corticosteroid use during study, No. (%) T = 56 (67) C = 54 (65)	Vitamin C at 50 mg/kg actual body weight infused intravenously every 6 h for total 96 h in 5% dextrose in water.	Placebo (5% dextrose in water alone).	All-cause mortality in 28 days, T = 25/84 C = 38/82 The ventilator-free days T = 13.1 C = 10.6 The ICU-free days to day 28 T = 10.7 C = 7.7 Transfer out of the ICU by hour 168 or less T = 21/84 C = 10/83 The hospital-free days T = 22.6 C = 15.5 Creatinine 0 h: T = 1.1(1.3)C = 1.7(1.8)48 h: T (82) = 1.6 (1.4); C(73) = 1.2 (1.0) 96 h: T(78) = 1.1(1.1); C(65) = 1.1(0.9) 168 h: T(64) = 1.0(1.2); C(55) = 1.2 (1.6)
Fujii et al., 2020 [53]	N = 211 T = 107 C = 104 Age, mean (SD), y T = 61.9 (15.9) C = 61.6 (13.9) Sex, No. (%) Male n (%) T = 68 (63.6) C = 65 (62.5) Female (%) T = 39 (36.4) C = 39 (37.5) Weight, median (IQR), kg T = 81.0 (66.0–95.0) C = 83.0 (67.5–102.0) Mechanical ventilation T = 66 (61.7) C = 65 (62.5) Norepinephrine T = 99 (92.5) C = 97 (93.3) Vasopressin T = 22 (20.6) C = 22 (21.2) Epinephrine T = 13 (12.1) C = 9 (8.7) Metaraminol T = 8 (7.5) C = 10 (9.6) Inotropes Milrinone T = 6 (5.6) C = 2 (1.9) RRT T = 12 (11.2) C = 12 (11.5) AKI, No. (%)h T = 74 (69.2) C = 75 (72.1) Stage 1 (mild) T = 27 C = 32 Stage 2 (moderate) T = 34 C = 23 Stage 3 (severe) T = 13 C = 20 APACHE III score T = 77.4 (29.7) C = 83.3 (28.8) SOFA score T = 8.6 (2.7) C = 8.4 (2.7) Serum creatinine, Median (IQR), mg/dL T = 1.73 (1.16–2.64); C = 1.78 (1.07–2.90)	IV vitamin C (1.5 g every 6 h), hydrocortisone (50 mg every 6 h) and thiamine (200 mg every 12 h).	Received IV hydrocortisone (50 mg every 6 h).	28-d Mortality, No. (%) T = 24/106 (22.6) [*n* = 106] C = 21/103 (20.4) [*n* = 103] 90-d Mortality, No. (%) T = 30/105 (28.6) [*n* = 105] C = 25/102 (24.5) [*n* = 102] ICU mortality, No. (%) T = 21/107 (19.6) C = 19/104 (18.3) Hospital mortality, No. (%) T = 25/107 (23.4) C = 21/103 (20.4) [*n* = 103] 28-d Cumulative vasopressor-free days, median (IQR) T = 25.6 (17.8 to 26.8) [*n* = 106] C = 25.8 (19.6 to 26.8) [*n* = 103] 28-d Cumulative MV-free days, median (IQR) T = 25.3 (5.2 to 28.0) [*n* = 106] C = 24.8 (9.5 to 28.0) [*n* = 103] 28-d RRT–free days, T = 28.0 (23.5 to 28.0) [*n* = 105] C = 28.0 (21.0 to 28.0) [*n* = 103] 28-d ICU-free days, T = 21.9 (0 to 25.8) [*n* = 106] C = 22.1 (3.9 to 25.8) [*n* = 103] Hospital LOS, d T = 12.3 (6.2 to 26.0) [*n* = 106] C = 12.3 (6.2 to 26.1) [*n* = 103] AKI (1,2,3) T = 75/107, C = 75/104 Stage 1 T = 18/107 (16.8) C = 14/104 (13.5) Stage 2 T = 18/107 (16.8) C = 22/104 (21.2) Stage 3 T = 39/107 (36.4) C = 39/104 (37.5)
Giladi et al., 2011 [24]	N = 4294 T = 2272 C = 2022 Male *n* (%) T = 1832 (81%) C = 1452 (72%) White race *n* (%) T = 1291 (79%) C = 1587 (78%) Mean age, years (SD) T = 40 ± 17 C = 39 ± 18 Mean admission GCS (SD) T = 12 ± 5 C = 13 ± 4 Mean ISS (SD) T = 20 ± 12.3 C = 21 ± 12.6 Mean predicted survival(SD) by TRISS methodology T = 0.822 ± 0.285 C = 0.885 ± 0.222 Renal failure, *n* (%) T = 30/2272 (1.3%) C = 26/2022 (1.3%)	Intravenous ascorbic acid 1000 mg intravenously in 100 mL of NS every 8 h, alpha-tocopherol 1000 IU (1 mL) via naso- or orogastric tube every 8 h, and selenium 200 mcg intravenously in 100 mL NS once daily. Ascorbic acid was administered as a bolus over 1 h and selenium as a bolus over 2 h, although both were permitted to be changed to an enteral dosage form once enteral access was established. The treatment course was 7 days or until discharge from hospital, whichever came first.	No antioxidants, only standard of care.	Mortality: T = 139/2272(6.1%) C = 172/2022(8.5%) ICU-LOS days, (IQR) T = 2 (1–4) C = 3 (1–6) Hospital LOS, days,(IQR) T = 3 (1–6) C = 4 (2–8). Ventilator free days (IQR) T = 3 (1–6) C = 3 (1–6). Respiratory failure, *n* (%) T = 395/2272 (17.4%) C = 558/2022 (27.6%) Ventilator-dependent respiratory failure, *n* (%) T = 160/2272 (7.1%) C = 218/2022 (10.8%) Renal failure, *n* (%) T = 30/2272 (1.3%) C = 26/2022 (1.3%) SIRS, *n* (%) T = 360/2272 (15.0%) C = 283/2022 (13.8%) ICU subset analysis Hospital stay T = 8.8 days C = 10.6 days ICU stay T = 3.9 days C = 4.9 days
Hwang et al., 2020 [49]	N = 111 T = 53 C = 58 Age (years) T = 70 (62–76)C = 69 (62–74) Sex (male) T = 20 (37.7) C = 22 (37.9) Chronic renal disease T = 3 (5.7) C = 0 (0) SOFA score T = 8 (6–10) C = 8 (6–10) APACHE II score T = 22 (14–32) C = 22 (17–32) Acute kidney injury Stage 1 T = 13/53 (24.5) C = 15/58 (25.9) Stage 2 T = 13/53 (24.5) C = 17/58 (29.3) Stage 3 T = 15/53 (28.3) C = 13/58 (22.4) Mechanical ventilation at enrolment T = 12 (22.6) C = 14 (24.1)	Vitamin C (50 mg/kg, maximum single dose 3 g, daily dose 6 g) and thiamine (200 mg) were mixed in a 50-milliliter 0.9% saline bag, respectively, and intravenously administered to patients over 60 min every 12 h for a total of 48 h.	Identical volume of 0.9% saline from the placebo drug ampoule was administered to patients using the same protocol.	7-day mortality T = 5/53 (9.4) C = 6/58 (10.3) 28-day mortality T = 11/53 (20.8) C = 9/58 (15.5) 90-day mortality T = 17/53 (32.1) C = 16/58 (27.6) In-hospital mortality T = 13/53 (24.5) C = 11/58 (19) ICU mortality T = 7/46 (15.2) C = 7/52 (13.5) Shock reversal T = 44/53 (83) C = 49 /58(84.5) Vasopressor-free days T = 11 (5–12) C = 11 (10–12) Mechanical ventilation (days) T = 6 (3–12) [*n* = 23] C = 7 (3–8) [*n* = 24] Ventilator-free days T = 11 (2–14) C = 11 (3–14) New use of RRT T = 9/52 (17.3) C = 11/58 (19) RRT-free days T = 14 (14–14) [*n* = 52] C = 14 (14–14) [*n* = 58] ICU LOS (day) T = 5 (3–11) [*n* = 46] C = 5.5 (4–12.5) [*n* = 52] ICU-free days T = 9 (3–11) C = 9 (0–11) Hospital LOS (day) T = 14 (11–21) C = 13.5 (9–26)
Iglesias et al., 2020 [54]	Total patients (N) = 137 T = 68 C = 69 Age T = 70 ± 12 C = 67 ± 14 Sex (male) T = 32 (47%) C = 27 (39%) ESRD T = 3 (0.4%) C = 0 (0%) CKD T = 10 (7%) C = 4 (2.9%) Mechanical ventilation T = 34 (50%) C = 35 (51%) Vasopressors T = 56 (82%) C = 47 (68%) AKI T = 54 (79%) C = 52 (75%) Day 1 SOFA T = 8.3 ± 3 C = 7.9 ± 3.5 APACHE II T = 24 ± 7.6 C = 24.9 ± 8.7 APACHE IV T = 88 ± 28.3 C = 87.5 ± 29.7	Ascorbic acid 1500 mg q 6 h, thiamine 200 mg every 12 h and hydrocortisone 50 mg q6 h.	A matching saline placebo for a maximum of 4 days.	Days of HAT therapy or placebo T = 3.3 ± 0.8 C = 3.25± 1 Vasopressor after study enrollment T = 4 (6%) C = 10 (14.5%) RRT for AKI T = 2/68 (3%) C = 8/69 (11.5%) ΔSOFA score at 72 h T = 2.9 ± 3.3 C = 1.93 ± 3.5 Duration of vasopressors, h T = 27 ± 22 C = 53 ± 38 Hospital mortality (%) T = 11/68 (16%) C = 13/69 (19.4%) ICU mortality (%) T = 6/68 (9%) C = 10/69 (14%) Hospital LOS, d T = 11.5 ± 6.8 C = 11 ± 6.2 ICU LOS, d T = 4.76 ± 4.3 C = 4.66 ± 3.45 Ventilator-free days T = 22 ± 6.2 C = 22.4 ± 4.3 AKI T = 54/68 (79%) C = 52/69 (75%) (Serum creatinine (mg/dL) SCr 24 h T = 1.74± 1.21 C = 1.85 ± 1.6 SCr 48 h T = 1.62± 1.32 C = 1.8± 1.71 SCr 72 h T = 1.47± 1.3 C = 1.67 ± 1.71 SCr at discharge T = 1.32± 1.13 C = 1.37± 1.18
Kahn et al., 2011 [25]	N = 33 [T = 17, C = 16] Age (yr) T = 42 ± 16 C = 50 ± 20 Male T = 16 C = 16 APACHE II T = 17 ±7 C = 18 ±8 Inhalation: T = 4 C = 4	LR based on Parkland formula with 66 mg/kg/hr VC administered during resuscitation until the patient was down to maintenance fluid (approximately 24 h).	LR solution only according to the Parkland formula.	Mortality T = 4/17 C = 3/16 Need of vasopressors, mean hours T = 6 ± 9, C = 11 ± 10 Renal failure: T = 2/17 C = 2/16 Pneumonia: T = 7/17 C = 10/16 Fasciotomy: T = 3/17 C = 3/16
Kim et al., 2018 [26]	N = 99 T = 53 C = 46 Male: T = 41 (77) C = 29 (63) Chronic kidney disease: T = 8 (15) C = 8 (17) ARDS at ICU admission T = 12 (23) C = 10 (22) APACHE II score at ICU admission T = 28 (21−32) C = 27 (22−32) SOFA score at ICU admission T = 11 (8–14) C = 11 (7–12) Use of MV in 1st day T = 43 (81) C = 36 (78) Vasopressor use in 1st day T = 33 (62) C = 22 (48) Use of RRT in 1st day T = 19 (36) C = 5 (11) Creatinine, mg/dL T = 1.1 (0.7–1.9) C = 0.8 (0.6–1.6)	Treatment group was treated with vitamin C protocol. The protocol consisted of intravenous vitamin C (1.5 g every 6 h for 4 days), hydrocortisone (50 mg every 6 h for 7 days followed by a taper over 3 days) and intravenous thiamine (200 mg every 12 h for 4 days). Treatment group was administered 6 g of vitamin C per day (divided into four equal doses).	The patients who were admitted to the same ICU between June 2016 and January 2017 were not treated with the vitamin C protocol and thus formed the control group.	At 28-day vasopressor-free days; T:18.8 ± 11.2, C: 19.6 ± 11.4 At 28-day ventilator-free days T: 10.7 ± 10.8, C:9.7 ± 10.9 ICU stay, d T:9 (4–14); C:12 (6–17) Hospital mortality: T:11/53 (21); C:17/46 (37) Among PPM cohort (T = 36, C = 36) At 28-day vasopressor-free; T = 19.8 ± 10.8, C = 20.5 ± 11.1 At 28-day ventilator-free days; T = 12.3 ± 11.0, C = 9.9 ± 10.7ICU stay, d T = 9 (5–14) C = 12 (7–17) Hospital mortality T = 6 (17) C = 14 (39)
Lin et al., 2018 [27]	N = 80 T = 38 C = 42 Age T = 41 ± 15 C = 42.4 ± 17 Inhalation injury T = 52% C = 36% Admission BUN (mg/dL) T = 12 (7–17) C = 12 (9–15) Admission Cr (mg/dL) T = 1.18 ± 0.6 C = 1.26 ± 0.5 mg/d	High dose ascorbic acid was started for Burn Shock Resuscitation at a dose of 66 mg/kg/hr, along with BSR based on Parkland formula calculations and received lactated Ringers for resuscitation.	Burn shock resuscitation (BSR) based on Parkland formula calculations and received lactated Ringers for resuscitation.	Mortality T = 10/38(26%) C = 10/42(24%) Ventilator days T = 11 (6–28) C = 5.5 (1–22) Hospital LOS (days) T = 29 (18–57) C = 24.5 (14–46) Abdominal Compartment Syndrome (ACS) T = 2/38 (5.2%) C = 1/42 (2.3%) AKI T = 9/38 (23%) C = 3/42 (7%) Ventilator-Associated Pneumonia (VAP) T = 11/38 (29%) C = 6/42 (14%) day 1 BUN (mg/dL) T = 12 (7–15) C = 12 (6–17) day 1 Cr (mg/dL) T = 1.26 ± 0.6 C = 1.42 ± 0.5 mg/d
Litwak et al., 2019 [28]	N = 94 (T = 47 C = 47) Age, mean SD, year T = 58.3 ±17.0 C= 60.1 ±14.0 Sex, male, No (%) T = 28 (59.6) C = 29 (61.7) CKD T = 4 (8.5) C = 10 (21.3) Mechanical ventilation, No (%) T = 43 (91.5) C = 39 (83.0) Vasopressors, No (%) T = 47 (100) C = 47 (100) AKI, No (%) T = 38 (80.9) C = 32 (68.1) Day 1 SOFA, mean SD T = 10.6 ± 10.6 C= 9.7 ± 10.0 APACHE II, mean SD T = 21.5± 8.0 C = 20.0 ±7.4 APACHE IV, T = 88.6 ± 29.1 C = 84.1 ± 25.4 Creatinine, median (IQR), mg/dL (excluding CKD) T = 1.4 (0.9–2.2) C = 1.4 (0.9–2.5)	At least one dose of each of the following medications intravenously: 1.5 g vitamin C every 6 h, 200–300 mg hydrocortisone daily (50 mg every 6 h or 100 mg every 8 h) and thiamine 200 mg every 12 h.	Standard of care but could receive hydrocortisone.	Hospital mortality T = 19/47 C = 19/47 ICU mortality T = 17/47 C = 18/47 RRT for AKI T= 11 of 38 C = 11 of 32 ICU LOS, days T = 11.0 (7.0–19.0) C = 10.0(5.0–17.0) Hospital LOS, days T = 19.0(9.0–26.0) C = 14.0 (8.0–23.0) Vasopressors, hours T = 84.2(37.0–169.3) C = 62.5(32.6–105.9) Change in SOFA score in 72 h, mean ± SD T = 1.3 ± 4.1 C= 0.1 ± 4.7 Sub group analysis Hospital mortality T = 7/20 C = 19/47 ICU mortality T = 6/20 C = 18/47 RRT for AKI T = 5 of 16 C= 11 of 32 ICU LOS days T = 14.5(7.5–22.0) C = 10.0 (5.0–17.0) Hospital LOS, days T = 19.0(9.3–23.5) C = 14.0(8.0–23.0)
Long et al., 2020 [29]	N = 206 T = 79 C = 127 Age, Yrs (mean, SD) T = 64.4 (13.9) C = 61.1 (16.2) Male gender *n*,% T = 43 (54.4) C = 71 (55.9) ESRD T = 5/79 (6.3) C = 17/127(13.4) Ventilator initiation, *n*, % T = 33 (41.7) C = 71 (55.9) Hydrocortisone use, *n*,% T = 79(100) C= 40 (31.5)	Hydrocortisone 50 mg IV every 6 h, vitamin C in a dose of 1.5 g IV every 6 h and thiamine 200 mg IV every 12 h, until the patient was either liberated from vasopressors, was discharged from the ICU or until 4 days of therapy had elapsed, whichever came first.	Standard of care: Surviving Sepsis Campaign Guidelines.	APACHE IV score T = 80.0 C = 88.2Observed ICU mortality, N (%) T = 9/79 (11.4) C = 33/127 (26.0) Hospital mortality T = 21/79 (26.6) C = 41/127 (32.3) Vasopressor duration, hours(median)T = 13.9 C = 24.2 New RRT initiation *n* (%)T = 11/74 (14.9%) C = 29/110 (26.4%) Ventilator duration days T = 3.4 C = 3.3 ICU LOS, days(median) T = 2.0 C = 2.5 Hospital LOS, days(median)T = 9.5 C = 9.1
Lv et al., 2020 [55]	N = 117 T = 61 C = 56 Sex, male, *n* (%) T = 30 (49.2) C = 29 (51.8) Age, mean ± SD, T = 58.7 ± 14.3 C = 60.2 ± 14.1 CRF, *n* (%) T = 3 (4.9) C = 4 (7.1) Ventilator, *n* (%) T = 31 (50.8) C = 28 (50.0) Vasoactive drugs, *n* (%) T = 35 (57.4) C = 33 (58.9) AKI, *n* (%)T = 29/61 (47.5) C = 27/56 (48.2) APACHE II, Median [IQR] T = 21.0 (19.0, 28.0) C = 23.0 (20.0, 29.0) Day 1 SOFA, mean ± SD T = 8.6 ± 2.9 C = 8.9 ± 3.1	Vitamin C from the day of entering ICU, administered intravenously by 3.0 g of vitamin C dissolved into 5% dextrose (100 mL/time, 2 times/day) until ICU discharge.	Intravenously by 5% dextrose (100 mL/time, 2 times/day) as placebo.	28-day mortality (%) T = 15/61 (24.6) C = 24/56 (42.9) ICU stay, day, Median [IQR] T = 4.1 (3.2, 8.3) C = 3.9 (3.1, 7.5) SOFA after72 h Median [IQR] T = 4.2 (1.2, 6.6) C = 2.1 (1.1, 4.3) Application time of vasoactive drugs [h, (IQR)] T = 25.6 (18.8, 40.6) C = 43.8 (24.7, 66.8)
Marik 2017 [5]	N = 94 [T = 47 C = 47] Sex, male, *n* T = 27 C = 23 Ventilation, N (%) T = 22 (47) C = 26 (55) Vasopressors, N (%) T= 22 (46) C = 22 (46) AKI, N (%)T = 31/47 (66) C = 30/47 (64) Day 1 SOFA, mean ± SD; T = 8.3 ± 2.8 C = 8.7 ± 3.7 APACHE II, mean ± SD; T= 22.1 ± 6.3 C = 22.6 ± 5.7; CRF T = 7/47 (15) C = 8/47 (17) Creatinine mean ±SD, mg/dL T = 1.9 ± 1.4 C = 1.9 ± 1.1	Intravenous vitamin C (1.5 g every 6 h for 4 days or until ICU discharge), hydrocortisone (50 mg every 6 h for 7 days or until ICU discharge followed by a taper over 3 days), as well as intravenous thiamine (200 mg every 12 h for 4 days or until ICU discharge).	Patients received hydrocortisone (50 mg every 6 h) at the discretion of the attending physician and all standard of care.	Hospital mortality, N; T = 4/47 C= 19/47 ICU LOS, median and IQR, d T = 4 (3–5) C = 4 (4–10) Vasopressors, mean ± SD, h T= 18.3 ± 9.8 C = 54.9 ± 28.4 Change in SOFA, 72 h T = 4.8 ± 2.4 C = 0.9 ± 2.7 RRT for AKI, No. (%) T = 3/31 (10%) C = 11/30 (33%)
Masood et al., 2019 [30]	N = 50 Mean age: 46.7 ± 18.4 (range: 16 to 77 years old) Gender: Male 28 (56); Female 22 (44)	IV thiamine 200 mg BD for 5 days along with IV vitamin C 1500 mg QID and IV hydrocortisone 50 mg QID for 5 days.	-	Median days in ICU= 8.3 [IQR = 5] Mortality, N = 24/50 (48%) SOFA score= 8.5 ± 1.5 Duration of vasopressor support < 24 h = 29 (58%) 24–48 h = 13 (26%)
Matsuoka et al., 2020 [31]	N = 17 T = 6 C = 11 Age (years) T = 67.7 ± 1.4 C = 69.7 ± 2.3 Male N (%) T = 4 (67) C = 10 (91) Both group received Methyl-prednisolone	Received ascorbic acid (1.5 g) and thiamine (200 mg) as per protocol.	Standard postoperative management.	Ventilation (days): T = 2.0 ± 0.0; C = 4.2 ± 0.3 ICU LOS (days): T = 3.2 ± 0.3; C = 6.9 ± 0.9 ARDS: T = 0 (0) C = 1 (9) Pneumonia T = 2 (33) C = 5 (45)
Mirmohammadsadeghi et al., 2018 [56]	N = 314 T = 160 C = 154 Age (mean years) T = 62.1 C = 62.7 Age >70 years T = 5 C = 6 Gender (male) T = 128 C = 116	2 g of vitamin C intravenously (IV) 24 h preoperatively, and postoperatively 500 mg every 12 h IV for 48 h in ICU, and 500 mg every 12 h PO for 48 h in ward.	Standard of care.	Postoperative stroke (%) T = 3/160 (1.9) C = 1/154 (0.7) Postoperative TIA (%) T = 0/160 (0) C = 2/154 (1.3) ICU stay (hours) T = 50.4 C = 52.5 Hospital stay (days) T = 8.13 C = 8.22 Atrial fibrillation (patients) T = 12/160 C = 12/154 Inotrope dependent T = 37/160 C = 39/154
Mitchell et al., 2019 [32]	N = 76 T = 38 C = 38 Mean age, years ± SD T = 68 ± 10 C = 68 ± 10 Male, *n* (%) T = 36 (95) C = 37 (97) Mean SOFA score ± SD T = 6.9 ± 4 C = 6 ± 3 Need for vasopressors, *n* (%) T = 30 (79) C = 29 (76 Ventilation, *n* (%) T = 19 (50) C = 17 (45) CKD/ESRD T = 9/38 (24) C = 8/38 (21)	Vitamin C was administered at a dose of 1.5 g IV every 6 h and thiamine 200 mg IV every 12 h for 4 days in addition to hydrocortisone.	IV prednisolone alone.	ICU mortality *n* (%) T = 12/38 (32) C = 13/38 (34) Hospital mortality *n* (%) T = 18/38 (47) C = 20/38 (53) 28 day Mortality *n* (%) T = 22/38 (58) C = 23/38 (61) 60 day Mortality *n* (%) T = 22/38 (58) C = 25/38 (66) AKI requiring RRT, *n* (%) T = 6/38 (16) C = 7/38 (18) Vasopressor duration, days T = 2.5 C = 4.8 Hospital LOS, days T = 20 C = 32.6 ICU LOS, days T = 7.1 C = 15.6
Mohamed et al., 2020 [57]	N = 88 T = 45 C = 43 Age, mean T = 58.69 ± 14.89 C = 59.37 ± 15.01 Male T = 31 (69) C = 32 (74) CKD T = 6 (14) C = 11 (25) GCS T = 15 (2–15) C = 15 (2–15) SOFA T = 11.22 ± 2.99 C = 10.89 ± 3.82 Creatinine (mg/dL) T = 2.8 ± 1.6 C = 2.5 ± 1.9	Intravenous hydrocortisone (50 mg every 6 h), vitamin C (AA) (1.5 g every 6 h) and thiamine (200 mg every 12 h) for 4 days.	Standard of care for septic shock.	Mortality (%) T = 26/45 (57) C = 23/43 (53) Reversal shock (hrs) T = 34.58 ± 22.63 C = 45.42 ± 24.4 Mechanical ventilation T = 22 (48.8) C = 20 (46.5) SOFA at 72 h T = 8.9 ± 3.6 C = 9.3 ± 3.8 Change in SOFA T = 2.23 ± 2.4 C = 1.38 ± 3.1 New onset of AKI T = 7/45 (15) C = 5/43 (12) ICU LOS T = 12.44 ± 14.2 C = 8.44 ± 8.16 Hospital LOS T = 31.58 ± 31.06 C = 20.9 ± 15.01
Moskowitz et al., 2020 [58]	N = 200 T= 101 C= 99 Age, mean y, (SD) T = 68.9 (15.0) C = 67.7 (13.9) Sex, Female T = 44 (43.6) C = 45 (45.5) Chronic kidney disease stage 2 (Mild) T = 1 (1.0) C = 1 (1.0) 3 (Moderate) T = 4 (4.0) C = 6 (6.1) 4 (Severe) T = 2 (2.0) C = 3 (3.0) Unknown 3 (3.0)3 (3.0) SOFA mean (SD) T = 9.1 (3.5) C= 9.2 (3.2) Ventilation, No. (%) T = 48 (47.5) C = 44 (44.4)	Ascorbic acid (1500 mg), hydrocortisone (50 mg) and thiamine (100mg) every 6 h for 4 days or until intensive care unit (ICU) discharge. Hydrocortisone was administered intravenously as a push dose in 1 mL of saline over 1 to 2 min.	0.9% sodium chloride in a matching volume (approximately 100 mL) using the same techniques at the same time points.	SOFA score at 72 h, mean (SD): T = 4.4 (4.1) [*n* = 90] C = 5.1 (4.3) [*n* = 88] 30 d, Mortality *n* (%) T = 35/101 (34.7) C = 29/99 (29.3) AKI *n* (%) T = 32/101(31.7) C = 27/99 (27.3) Ventilator-free days, median (IQR) T = 6 (2–7) C = 6 (0–7) Shock-free days, median (IQR) T = 5 (3–5) C = 4 (1–5) Incidence of delirium, No./total (%) T = 31/83 (37.4) C = 35/76 (46.1) ICU-free days, median (IQR) T = 22 (3–25) C = 21 (4–25)
Nagel et al., 2020 [33]	N = 38 T = 19 C = 19 Age (years) T = 47 ± 18.8 C = 49 ± 17.9 Gender (female:male) T = 6 13 C = 6:13 ABSI(abbreviated burn severity index) T = 7 (5–12) C = 7 (4–12) Admission Crea (mg/dL): T = 0.99 ± 0.34 C= 1.14 ± 0.64	High dose ascorbic acid was infused continuously over 24 h with 66 mg/kg per hour intravenously.	Low dose ascorbic acid 3.5 g as a single infusion.	Mortality (%) T (HDAA) = 5/19 (26.3) C (LDAA) = 2/19 (10.5) 24-hr Vasopressor use (%) T = 5/19 (26.3) C = 8/19(42.1) 24–72 hrs Vasopressor use T = 5/19(26.3); C = 10/19 (52.6) Hospital LOS (d) T = 39 ± 33 C = 58 ± 46 Pneumonia (%) T = 2/19 (10.5) C = 2/19(10.5) Ventilation (d) T = 19.5 ± 26.0 C = 10.1 ± 20.9 AKI (AKIN 1, 2, 3): T = 15/19; C = 13/18 RRT (%) T = 4/19 (21.0) C = 3/19 (15.8) 24-hr Crea (mg/dL): T = 1.85 ± 0.9 C = 1.67 ± 1.18 72-hr Crea (mg/dL): T = 1.27 ± 0.76 C = 1.32 ± 1.23
Nakajima et al., 2019 [7]	Before PSM N = 2713 T = 157 C = 2556 Age (years), median (IQR) T = 67 (48–80) C = 69 (51–80) Male, *n* (%) T = 87 (55.4) C = 1561 (61.1) Inhalation injury, *n* (%) T = 45 (28.7) C = 523 (20.5) After PSM N = 785 T = 157 C = 628 Age(years), median (IQR) T = 67 (48–80) C = 67.5 (50–80) Male n(%) T = 87 (55.4) C = 332 (52.9) Inhalation injury n (%) T = 45 (28.7) C = 181 (28.8)	High dose vitamin C was administered under the following two varying thresholds: dosage more than (1) 10 g within 2 days of admission and (2) 24 g within 2 days of admission. High-dose vitamin C administered as a continuous intravenous infusion during the initial 24 h period after admission.	Standard of care.	In hospital mortality 10 g threshold of high dose Vitamin C T = 72/157 C = 364/628 24 g threshold of high dose Vitamin C T = 58/127 C = 278/508
Nathens et al., 2002 [59]	N = 595 T = 301 C = 294 Age (mean± SD) T = 38± 15 C = 39 ± 15 Gender Male T = 230 (76) C = 222 (76) Illness severity ISS (mean ±SD) T = 19.1± 10 C = 20 ± 11 APACHE II (mean ±SD) T = 13.6± 6 C = 14.3± 6	Alpha-tocopherol 1000 IU (20 mL) q8h per naso- or orogastric tube and 1000 mg ascorbic acid given intravenously in 100 mL of D5W q8h for the shorter duration of admission to the ICU or 28 days.	Standard of care.	Duration of ventilation (mean days) T = 3.7 C = 4.6 Ventilator-free days (mean) T = 24.1 C = 23.3 Multiple organ dysfunction score (mean) T = 2.4 C = 3.2 ICU length of stay, days (mean) T = 5.3 C = 6.4 Hospital length of stay (mean) T = 14.6 C = 15.1 Multiple organ failure T = 8/301 (2.7%) C = 18/294 (6.1%) Mortality 28-day T = 4/301 (1.3%) C = 7/294 (2.4%) ICU T = 3/301 (1.0%) C = 9/294 (3.1%) Hospital T = 5/301 (1.7%) C = 9/294 (3.1%)
Palli et al., 2017 [60]	N = 124 [T = 60 C = 64] Age (yrs) T= 51.34 (2.71) C = 50.51 (2.65) Male n (%) T = 46 (76.66) C = 55 (85.93) APACHE II score T = 14.44 (1.01) C = 13.29 (0.78) SOFA score T = 5.94 (0.5) C = 6.14 (0.41)Ventilator T= 32/60 (53.33) C = 44/64 (68.75) Vasoactive therapy, n (%) T = 20/60 (33.33) C = 25/64 (39.06)	Intravenously receive NAC (1200 mg) and ascorbic acid (2 g) dissolved separately in 100 mL of normal saline (N/S) 0.9%, 2 h before and at 10 h and 18 h following the infusion of contrast agent.	200 mL of intravenous N/S 0.9%.	ICU LOS (days, median) T = 32.5 (5.11) C = 27.5 (4.49) ICU mortality, *n* (%) T = 15/60 (25) C = 11/64 (17.18) RRT 10 days post contrast agent, *n* (%) T = 3/60 (5) C = 4/64 (6.25)
Park et al., 2020 [34]	Before Matching N = 435(T = 94, C = 341) Male [T = 55 (58.5), C = 229 (67.1)] Age: T = 69 (60–76), C = 69 (61–76) CKD T = 7/94 (7.5) C = 23/341 (10.3) Creatinine (mg/dL) T = 2.1 ± 1.5 C = 1.8 ± 1.6 APACHE II score T = 30.2 ± 8.1 C = 28.4 ± 9.2 SOFA score T = 11.5 ±3.4 C = 10.1 ± 3.7 Mechanical ventilation, T = 53 (56.4) C = 173 (50.7) After Matching N = 178(T = 89, C = 89) Male: [T = 52 (58.4) C = 55 (61.8)] Age: T = 69 (61–76), C = 71 (62–78) CKD T = 7/89 (7.8) C = 9/89 (10.1) Creatinine (mg/dL) T = 1.9 ± 1.4 C = 2.2 ± 2.3 APACHE II score T = 30.0 ± 7.9 C = 30.0 ± 8.9 SOFA score T = 11.4 ± 3.5 C = 11.5 ± 3.4 Mechanical ventilation, T = 49 (55.1) C = 48 (53.9)	Vitamin C (3 g/12 h or 1.5 g/6 h) and thiamine (200 mg/12 h) mixed in 50- or 100-milliliter solution bags of 5% dextrose in water or normal saline were administered intravenously within 6 h of shock recognition.	Protocol-driven therapies based on the updated Surviving Sepsis Campaign guidelines were provided for all included patients.	Before Matching Delirium free days T= 11 C = 13 Delirium coma free days T = 13 C = 11 Incidence of delirium = 62/94 C = 194/341 Duration of delirium, days T= 2 C = 1 Hospital stay, days T = 16 C = 13 ICU stay, days T = 4 C= 4 28-day mortality T = 23/94 C = 72/341 Among matched cohort Delirium free days T = 11 C = 12 Delirium coma free days T = 11 C= 12 Incidence of delirium T = 57/89 C = 54/89 Hospital stay days median (IQRs) T = 16 (8–27); C= 13 (7–28) ICU stay days T= 4 (3–7); C= 4 (3–7) 28-day mortality T= 21/89; C= 16/89
Reddy et al., 2020 [61]	N = 19 [T1 = 7 T2 = 5 C = 7] Age mean (SD) T1 = 56.5 (12) T2 = 53.8 (11) C = 55.4 (12.3) Male T1= 3 (15.7) T2 = 4 (21) C = 3 (15.7) APACHE score, mean (SD) T1= 17 (3.8) T2 = 18.4 (2.5) C = 21.2 (6.5) SOFA score, mean (SD) T1 = 11 (3.4) T2 = 9.2 (0.4) C = 8.8 (2.9)	T1 (hydrocortisone + vitamin C)- Hydrocortisone 200 mg over 24 h infusion and ascorbic acid in dose of 1.5 g IV every 6 h T2 (hydrocortisone + vitamin C + thiamine)- Hydrocortisone 200 mg over a 24-hour infusion, ascorbic acid in dose of 1.5 g IV every 6 h and thiamine dose of 200 mg IV every 12 h	Hydrocortisone was administered as 200 mg over a 24-hour infusion only.	Time to shock reversal (minutes), mean (SD) T1(7) = 2525 (3086); T2 (5) = 1860 (749); C(5) = 7422 (8348) Time to vasopressor reduction (minutes) from SOFA (h) 4–3 mean (SD) T1 (5) = 2310 (2515); T2 (5) = 1800 (1282); C (6) = 4935 (6406)
Sadaka et al., 2020 [35]	N = 62; T = 31, C = 31 Age, years (SD) T = 67 (16) C = 70 (12) Male, *n* (%) T = 16 (52) C = 16 (52) APACHE III score (SD) T = 95 (30) C = 96 (29) Mechanical ventilation, *n* (%) T = 27 (87) C = 27 (87) Creatinine (SD) T = 2.6 (2.4) C = 2.4 (1.9)	Intravenous (IV) ascorbic acid (1.5 g every 6 h for 4 days), hydrocortisone (50 mg every 6 h for 7 days) and thiamine (200 mg every 12 h for 4 days).	Patients were managed as per sepsis management guidelines.	ICU mortality, *n* (%) T = 3/31 (9.6) C = 13/31 (42.0) Hospital mortality, *n* (%) T = 9/31 (29) C = 14/31 (45) ICU LOS, days, median (IQR) T = 6.4 (1.2–9.6) C = 4.0 (2.5–9.2) LOHS, days, median (IQR) T = 15.0 (10.0–22.0) C = 9.3 (3.7–19.5) RRT for AKI, *n* (%) T = 8/31 (26) C = 9/31 (29) Duration of Vasopressors, days, media (IQR) T = 4.5 (4.0–6.0) C = 2.0 (1.0–3.0) Ventilation-free days, median (IQR) T = 10.2 (5.0–15.0) C = 10.2 (1.6–18.0)
Sadeghpour et al., 2015 [62]	N = 290; T = 113, C = 177 Age, y T = 57.28 ± 14.09 C = 54.22 ± 14.39 Male T = 80 (70.8) C = 111 (62.7) Intubation time, h T = 11.83 ± 3.91 C = 14.14 ± 9.52	A total of 2 g of vitamin C intravenously, immediately before surgery in the operating theatre, followed by 1 g daily oral doses of the tablets for the first four postoperative days.	Equal number of identical placebo tablets.	ICU Stay T = 3.42 ± 1.06 C = 3.43 ± 1.09 Hospital Stay T = 10.17 ± 4.63 C = 12 ± 4.51 Death, Impaired Renal Function and Infection T = 4/113 (3.54) C = 18/177 (10.2)
Sandesc et al., 2018 [36]	N = 67; T = 35 C = 32 Age (yrs), mean (SD) T = 45.62 (16.88) C = 42.67 (16.72) Gender (male), % (*n*) T = 74.28 (26) C = 75 (24) APACHE II, mean (SD) T = 11.74 (7.11) C = 12.09 (15.94) Lac (mmol/mL) T = 4.89 (1.99) C = 5.1 (2.5)	Antioxidant therapy included continuous intravenous sodium ascorbate 3000 mg/24 h and N-acetylcysteine 1200 mg/24 h.	Antioxidant Therapy.	Sepsis, *n* (%) T = 13/35 (37.14) C = 28/32 (88.75) MODS, *n* (%) T = 8/35 (22.85) C = 21/32 (65.62) Mechanical ventilation > 96 h, *n* (%) T = 19/35 (54.28) C = 17/32 (53.21) ICU LOS, mean (SD) T = 14.4 (16.02) C = 18.25 (32.55) LOHS days, T = 26.03 (20.47) C = 38.68 (40.17) Mortality, *n* (%) T = 5/35 (14.28) C = 11/32 (33.6)
Shin et al., 2019 [37]	N:1144 T:229 C:915 Age N:67 (58–75) T:67 (58–76) C:67 (60–75) Male N:713 (62.3) T:136 (59.4) C:577 (63.1) CKD N:78 (6.8) T:15 (6.6) C:63 (6.9) Vasopressor use N:966 (84.4) T:217 (94.8) C:749 (81.9) Mechanical ventilation N:328 (17.9) T:67 (39.3) C:261 (28.5) SOFA in 24 h N:8 (5–11) T:9 (6–12) C:8 (5–11) APACHE II N:20 (15–27) T:27 (21–52) C:27 (20–56)	Within 6 h of shock recognition, vitamin C and thiamine were mixed in 50- or 100-milliliter solution bags of 5% dextrose in water or normal saline and intravenously administered for 1 day (vitamin C, 3 g/12 h or 1.5 g/6 h; thiamine, 200 mg/12 h).	All patients with septic shock were treated with protocol-driven resuscitation bundle therapy based on the Surviving Sepsis Campaign guidelines.	Overall Cohort 28-day mortality: T: 42/229 (18.3); C:160/915 (17.5) In-hospital mortality: T:38/229 (16.6); C:167/915 (18.3) ICU stay (days) T:4 (3–8); C:4 (3–8) Hospital stay (days); T:14 (9–22); C:13 (8–23) Ventilation T:6.0(3.0–15.0); C:6.0(3.0–12.0) New use of RRT T:28 (12.3); C:106 (11.9) Propensity-Matched Cohort (T:227, C:527 28-day mortality T:42/227 (18.5); C:92/527 (17.5) In-hospital mortality: T:38/227 (16.7); C:97/527 (18.4) ICU stay (days) T:4 (3–8); C:4 (3–7) Hospital stay (days) T:14 (9–22); C:13 (8–23) Ventilation T:5.5 (3.0–15.0); C:5.0 (3.0–10.0) New use of RRT T = 28/227 (12.4) C = 66/527 (12.9)
Siriwardena et al., 2007 [63]	N = 43 T = 22 C = 21 Age (SD) T = 64 (13) C = 71 (14) Sex (% male) T = 8(36%) C = 7(33%) APACHE admission (SD) T = 10.5 (3.4) C = 11.0 (3.5) MODS at admission (IQR) T = 1.36(0–2) C = 1.19 (0–2)	Selenium in 50 mL of normal saline, ascorbic acid in 100 mL of normal saline and *n*-Acetylcysteine as per protocol.	Maximal conventional therapy plus intravenous placebo for 7 days.	Organ dysfunction (day 7) Organ dysfunction (%) T = 7/22 (32) C = 4/21 (19) APACHE (SD) T = 5.73 (3.28) C = 5.33 (1.06) Renal dysfunction (%) T = 11/22 (50) C = 7/21 (33) LOHS (days)T = 20.4 (24.4) C = 14.3 (15.7) Days in ICU T = 4.0 (10.3) C = 0.0 (0.0) All-cause mortality T = 4/22 (18.2%) C = 0/21 (0.0%)
Vail et al., 2020 [38]	N = 338,597, T = 3574; C = 335,023 Female, N (%) T = 1671 (46.8) C = 163,190 (48.7) Age, mean ±SD, years T = 64.6 ± 14.8 C = 66.1 ± 14.7 RRT at admission, N (%) T = 999 (28.0) C = 52,015 (15.5)	At least one charge for high-dose IV ascorbic acid (one or more vials totaling ≥500 mg in one calendar day) and at least one charge for both IV hydrocortisone and IV thiamine (of any dose) on or within one day of ascorbic acid administration	Routine treatment of septic shock without hydrocortisone, ascorbic acid or thiamine.	LOHS, median (IQR), days Survivors T = 12 (7, 20) C = 10 (6, 17) Non survivors T = 5 (2, 12) C = 5 (2, 12) ICU stay: Survivors T = 6 (3, 11) C = 4 (2, 8) Non survivors T = 4 (2, 9) C = 3 (1, 8) IMV T = 2625/3574 (73.4) C = 201,229/335,023 (60.1) NIMV T = 1175/3574 (32.9) C = 104,970/335,023 (31.3) Vasopressor use, Survivors T = 2 (1, 4) C = 2 (1, 3) Non-survivors T = 3 (2, 6) C = 2 (1, 4) Hospital mortality T = 1459/3574 (40.8) C = 92,060/335,023 (27.5)
Wang et al., 2020 [64]	N = 70 T = 33 C = 37 Age (years) T = 59.15 ± 8.11 C = 55.00 ± 9.92 M/F T = 13 (39.39)/20 (60.61) C = 14 (37.84)/23 (62.16)	Intravenous vitamin C 1 g (diluted to 10 mL) with a total of 3 g, 10 min after induction of anesthesia, 10 min before cardiac reanimation and at the moment of sternal closure.	Intravenous 10 mL saline.	ICU stay (h) T = 39.5 (20.8–44.3) C = 39.5 (20.5–44.5) LOHS (days) T = 14 (10–15) C = 12 (11–17) Atrial fibrillation T = 10/33 (30.30) C = 10/37 (27.03) Extubation time (min) T = 660 (401–920) C = 660 (358–862) Pulmonary complication T = 4 (12.12) C = 12 (32.43)
Wani et al., 2020 [48]	N = 100 T = 50 C = 50 Age (Median, IQR) T = 65 (59, 25–72) C = 70 (56, 25–72) Male: *n* (%) T = 28 (56%) C = 31 (62%) APACHE II Median (IQR) T = 18.5 (15–24.75) C = 20 (15–24) SOFA Mean ± SD (Range) T = 9.22 ± 3.54 (4–16) C = 9.36 ± 3.66 (2–16) Renal disease *n* (%) T = 6 (12.0%) C = 4 (8.0%) Acute kidney injury *n* (%) T = 37 (74%) C = 39 (78%)	Intravenous vitamin C (1.5 g q 6 hourly for 4 days or until discharge from the hospital), hydrocortisone (50 mg q 6 hourly for 7 days or until ICU discharge followed by a taper over 3 days) and intravenous thiamine (200 mg q 12 hourly for 4 days or until discharge from the hospital).	Standard of care, consisted of broad-spectrum antibiotics, intravenous fluids, vasopressors and mechanical ventilation as indicated.	Hospital mortality N (%) T = 12/50 (24%) C = 14/50 (28%) Ventilation N (%) T = 3/50 (6%) C = 3/50 (6%) Duration of vasopressor use (Mean ± SD, hours) Range T = 75.72 ± 30.29 (20–140) C = 96.13 ± 40.50 (30–200) LOHS (Mean ± SD, days) (Range) T = 11.82 ± 7.36 (4–36) C = 10.70 ± 6.39 (2–27) 30 day mortality N (%) T = 20/50 (40%) C = 21/50 (42%) SOFA score Day 4 T = 5.64 ± 3.55 C = 6.62 ± 3.94
Yanase et al., 2020 [65]	N = 50 T = 25 C = 25 Age, years, (IQR) T = 67.0 (82.0, 74.0) C = 64.0 (59.0, 69.0) Sex, male T = 15/25 (60.0%) C = 23/25 (92.0%) Serum creatinine (μmol/L): T = 85.0 (75.0, 98.0) C = 92.0 (80.0, 100.0)	Vitamin C 1500 mg in normal saline (100-milliliter bag) administered 6 hourly in the vitamin C group.	Normal saline (100-milliliter bag) administered 6 hourly in the placebo group.	ICU LOS, days T = 1.4 (0.5, 2.5) C = 1.5 (0.5, 3.3) LOHS, days T = 13.2 (7.9, 20.2) C = 12.5 (8.1, 16.7) ICU mortality T = 1/25 (4.0%) C = 0/25 (0.0%) Hospital mortality T = 1/25 (4.0%) C = 0/25 (0.0%) AKI (1, 2, 3): T = 14/25; C = 12/25 Stage 1 T = 3/25 (12.0%) C = 1/25 (4.0%) Stage 2 T = 10/25 (40.0%) C = 9/25 (36.0%) Stage 3 T = 1/25 (4.0%) C = 2/25 (8.0%) Atrial fibrillation T = 11/25(44.0%) C = 8/25(32.0%)
Yoo et al., 2020 [39]	N = 79 T = 33 C = 46 Age, yr T = 66 (55.5–81) C = 73.5 (63–79) Male sex T = 20 (60.6) C = 34 (73.9 CKD T = 4 (12.1) C = 3 (6.5) APACHE II T = 26 (20.5–32.5) C = 30 (24–33.3) SOFA T = 13 (11–15) C = 12 (10–14.3) AKI T = 18 (54.5) C = 29 (63) RRT T = 7 (21.2) C = 13 (28.3)	The total supplementation of vitamin B1 was 200 mg/day intravenously infused 50 mg every 6 h. A total of 2 g of vitamin C intravenously infused 500 mg every 6 h.	No vitamin B and C supplementation, only standard of care.	14-Day mortality T = 15/33 (45.5) C = 22/46 (47.8) 30-Day mortality T = 18/33 (54.5) C = 32/46 (69.6) ICU mortality T = 18/33 (54.5) C = 32/46 (69.6) In hospital mortality T = 19/33 (59.4) C = 33/36 (71.7) Ventilator free days T = 7.7 ± 10.8 C = 2.7 ± 7.3 ICU-free days T = 6.9 ± 9.8 C = 2.2 ± 6.5
Zabet et al., 2016 [6]	N = 28 T = 14 C = 14 Age years, SD T = 64.14 ± 15.98 C = 63.71 ± 13.84 Sex (male) T = 10 (71.42) C = 11 (78.57) APACHE II score T = 19.07 ± 5.18 C = 23 ± 5.61 SOFA score T = 11.78 ± 2.22 C = 12.35 ± 3.00	25 mg/kg intravenous ascorbic acid every 6 h for 72 h.	50 mL of dextrose 5% solution as intravenous infusion over 30 min.	ICU LOS (days) T = 21.45 ± 10.23 C = 20.57 ± 13.04 28-day mortality T = 2/14 (14.28) C = 9/14 (64.28) Duration of norepinephrine administration (h) T = 49.64 ± 25.67 C = 71.57 ± 1.60
Ferron Celma et al., 2009 [47]	N = 20 T = 10 C = 10 Age (y) T = 67.8 ± 4.5 C = 65.1 ± 3.6 POSSUM Score T = 55.0 ± 3.3 C = 50.4 ± 1.4 Sex (Men/Women) T = 5/5 C = 6/4 Pressors T = 3/10 C = 2/10 Ventilatory support T = 3/10 C = 2/10 Creatinine (mg/dL) T = 2.15 ± 0.12 C = 1.99 ± 0.21 Lactate (meq/L) T = 3.22 ± 1.25 C = 1.97 ± 0.55	450 mg/day of the vitamin C administered in 5% dextrose water in 3 divided doses.	Identical administration of 5% dextrose.	Mortality T = 6/10 C = 4/10
Habib et al., 2017 [66]	N = 100 T = 50 C = 50 Male T = 28 (56) C = 30 (60) Age Mean ± SD. T = 42.78 ± 9.49 C = 41.70 ± 10.2 APACHE II T = 22.0 ± 6.70 C = 21.8 ± 7.07 SOFA Mean ± SD. T = 10.2 ± 3.42 C = 11.4 ± 5.08 BUN mg/dL T = 100.23 ± 11.31 C = 100.40 ± 17.30 S. Cr mg/dL T = 3.22 ± 2.21 C = 5.83 ± 0.16	Intravenous 1.5 gm vitamin C (ascorbic acid, Cevarol) every 6 h in the first 24 h after admission until ICU discharge plus conventional sepsis treatment.	Conventional sepsis treatment only.	Vasopressor Mean ± SD. T = 2.30 ± 1.2 C = 6.50 ± 2.57 Ventilator Mean ± SD. T = 4.60 ± 2.08 C = 7.87 ± 3.01 ICU LOS Mean ± SD. T = 10.00 ± 5.50 C = 14.10 ± 6.47 RRT T = 15/50 (30%) C = 13/50 (26%) Survivors T = 38/50 (76%) C = 32/50 (64%) Non-survivors T = 12/50 (24%) C = 18/50 (36%)
Tanaka et al., 2000 [67]	N = 37 T = 19 C = 18 Age, mean ±SD, y T = 40 ± 20 C = 49 ± 22 Male T = 13 C = 12 Inhalational injuries T = 15/19 C = 12/18	An injectable ascorbic acid solution was diluted 10-fold with distilled water. The ascorbic acid solution was administered as a continuous intravenous infusion.	Fluid resuscitation without ascorbic acid.	LOHS, mean ± SD, d T = 40 ± 28 C= 49 ± 44 Ventilation, mean ± SD, d T = 12.1 ± 8.8 C = 21.3 ± 15.6 Mortality, no of patients T = 9/19 C = 7/18
Galley et al., 1997 [68]	N = 30 T = 16 C = 14 Age median (range) T = 67 (21–78) C = 70 (22–89) Male: Female T = 13:3 C = 9:5 APACHE II median (range) T = 27.5 (17–54) C = 24 (11–35)	Intravenous antioxidant therapy comprising *n*-acetylcysteine 150 mg/kg for 30 min then 20 mg/kg/h plus bolus doses of 1 g ascorbic acid (vitamin C) and 400 mg alpha-tocopherol (vitamin E).	Comparable volume of 5% dextrose as placebo.	Mortality, *n* T = 11/16 C = 8/14
Razmkon et al., 2011 [69]	N = 76 T1 = 26 T2 = 23 C = 27 Age (y; mean, range) T1 = 31.1 (16–67) T2 = 29.5 (19–75) C = 29.4 (16–68) Sex (male/female) T1 = 20/6 T2 = 20/3 C = 23/4 Admission GCSs (mean, range) T1 = 5.9 (3–8) T2 = 6.1 (3–8) C = 6.5 (3–8)	T1 group, low-dose vitamin C (500 mg/d IV) for 7 days; T2 group, high-dose vitamin C (10 g IV on the first (admission) day and repeated on the fourth day, followed by vitamin C 4 g/d IV for the remaining 3 days	Identical placebo.	Hospital mortality T1 = 7/26 T2 = 7/23 C = 8/27 Mortality after 2 months T1 = 8/26 T2 = 7/23 C = 8/27 Mortality after 6 months T1 = 9/26 T2 = 7/23 C = 8/27
Bansal et al., 2011 [70]	N = 39 [T = 19, C = 20] Age (years) Mean ±SD T = 39.9 (10.9) C = 38.6 (11.4) Males (%) T = 15 (79) C = 15 (75) APACHE score (SD) T = 11.2(2.9) C = 11.5 (2.7)	vitamin C (1000 mg in 100 mL of normal saline), vitamin E (200 mg oral) and vitamin A (10,000 IU intramuscularly).	The control group was given the standard treatment only.	Organ dysfunction(day 7) T = 7 /19 (37) C = 8/20 (40) LOHS (days) Mean (SD) T = 12.8 (3.9) C = 15.1 (5.43) Renal insufficiency T = 4/19 C = 4/20 Mortality T = 0/19 C= 2/20
Sevransky et al., 2021 [46]	N = 501(T = 252, C = 249) Median age, 62 [IQR, 50–70] years Female: 46%	Intravenous vitamin C (1.5 g), thiamine (100 mg) and hydrocortisone (50 mg) every 6 h.	Matching placebo.	Mortality in 30-day: T:56 /252; C: 60/249 Mortality 180-day: T:102 /252; C: 94/249 ICU mortality: T:52 /252; C: 49/249 ICU stay, median (IQR), day; T:4 (2–8) [*n* = 250] C:4 (2–8) [*n* = 245] Hospital stay, median (IQR), day; T: 10 (6–17) [*n* = 250]; C: 9 (5–17) [*n* = 246] Ventilator- and vasopressor-free days {median(IQR)}: T: 25 (0–29) days; C: 26 (0–28) days

## Data Availability

The datasets analyzed during the current study are available from the corresponding author on reasonable request.

## Supplementary Materials

The following are available online at https://www.mdpi.com/article/10.3390/nu13103564/s1, Supplementary file S1: Electronic search details, Supplementary file S2. Basic study details, Supplementary file S3: Additional analysis.
