# Supplementary material for: Vitamin C in Critically Ill Patients: An Updated Systematic Review and Meta-Analysis
Source: Nutrients. 2021 Oct 12;13(10):3564. doi: 10.3390/nu13103564 (PMC8539952; doi:10.3390/nu13103564)
Supplement: Supplementary file 1 [file nutrients-13-03564-s001.zip › Supplementary file S1. Electronic search details.pdf]

Supplementary file S1. Electronic search details

Vitamin C in critically ill patients: An Updated Systematic Review and Meta-analysis

Dhan Bahadur Shrestha, MD[1], Pravash Budhathoki, MD[2], Yub Raj Sedhai, MD[3*], Sujit Kumar Mandal, MBBS[4], Shreeja Shikhrakar, MBBS[5], Saurab Karki, MBBS[6], Ram Kaji Baniya, MD[7] Markos G. Kashiouris, MD MPH[8], Xian Qiao MD[9], and Alpha A. Fowler, III, MD[8]

[1]Department of Internal Medicine, Mount Sinai Hospital, Chicago, IL, USA

[2]Department of Internal Medicine, Bronxcare Health System, Bronx, NY, USA

[3]Department of Internal Medicine, Division of Hospital Medicine, Virginia Commonwealth University, School of Medicine, Richmond, VA, USA

[4]Nepalese Army Institute of Health Sciences, Kathmandu, Nepal

[5]Kathmandu University School of Medical Sciences, Dhulikhel, Kavrepalanchok, Nepal

[6]Military Hospital, Itahari-4, Sunsari, Nepal

[7]Our Lady of the Lake Regional Medical Center, Baton Rouge, LA, USA

[8]Department of Internal Medicine, Pulmonary and Critical Care, Virginia Commonwealth University, School of Medicine, Richmond, VA, USA

[9]Department of Internal Medicine, Pulmonary and Critical Care, Eastern Virginia Medical School Norfolk, VA, USA

Pubmed

Hits: 884

("vitamin c" OR "ascorbic acid" OR "ascorbate") AND ("sepsis" OR "septic shock" OR "critical illness" OR "Intensive Care Unit" OR "ICU" OR "burn" OR "ARDS" OR "trauma")

https://pubmed.ncbi.nlm.nih.gov/?term=+%28%22vitamin+c%22+OR+%22ascorbic+acid%22+OR+%22ascorbate%22%29+AND+%28%22sepsis%22+OR+%22septic+shock%22+OR+%22critical+illness%22+OR+%22Intensive+Care+Unit%22+OR+%22ICU%22+OR+%22burn%22+OR+%22ARDS%22+OR+%22trauma%22%29

Pubmed Central

Hits: 15313

("vitamin c" OR "ascorbic acid" OR "ascorbate") AND ("sepsis" OR "septic shock" OR "critical illness" OR "Intensive Care Unit" OR "ICU" OR "burn" OR "ARDS" OR "trauma")

https://www.ncbi.nlm.nih.gov/pmc/?term=(%22vitamin+c%22+OR+%22ascorbic+acid%22+OR+%22ascorbate%22)+AND+(%22sepsis%22+OR+%22septic+shock%22+OR+%22critical+illness%22+OR+%22Intensive+Care+Unit%22+OR+%22ICU%22+OR+%22burn%22+OR+%22ARDS%22+OR+%22trauma%22)

Embase

Hits: 112

Search: https://www.embase.com/#advancedSearch/resultspage/history.3/page.1/25.items/orderby.date/source.

Scopus

Hits: 2000

("vitamin c" OR "ascorbic acid" OR "ascorbate") AND ("sepsis" OR "septic shock" OR "critical illness" OR "Intensive Care Unit" OR "ICU" OR "burn" OR "ARDS" OR "trauma")

https://www.scopus.com/results/results.uri?numberOfFields=0&src=s&clickedLink=&edit=&editSaveSearch=&origin=searchbasic&authorTab=&affiliationTab=&advancedTab=&scint=1&menu=search&tablin=&searchterm1=%28%22vitamin+c%22+OR+%22ascorbic+acid%22+OR+%22ascorbate%22%29+AND+%28%22sepsis%22+OR+%22septic+shock%22+OR+%22critical+illness%22+OR+%22Intensive+Care+Unit%22+OR+%22ICU%22+OR+%22burn%22+OR+%22ARDS%22+OR+%22trauma%22%29&field1=TITLE_ABS_KEY&dateType=Publication_Date_Type&yearFrom=Before+1960&yearTo=Present&loadDate=7&documenttype=All&accessTypes=All&r

esetFormLink=&st1=%28%22vitamin+c%22+OR+%22ascorbic+acid%22+OR+%22ascorbate%22%29+AND+%28%22sepsis%22+OR+%22septic+shock%22+OR+%22critical+illness%22+OR+%22Intensive+Care+Unit%22+OR+%22ICU%22+OR+%22burn%22+OR+%22ARDS%22+OR+%22trauma%22%29&st2=&sot=b&sdt=b&sl=183&s=TITLE-ABS-KEY%28%28%22vitamin+c%22+OR+%22ascorbic+acid%22+OR+%22ascorbate%22%29+AND+%28%22sepsis%22+OR+%22septic+shock%22+OR+%22critical+illness%22+OR+%22Intensive+Care+Unit%22+OR+%22ICU%22+OR+%22burn%22+OR+%22ARDS%22+OR+%22trauma%22%29%29&sid=b0edf479bae139c7f8ea1d1d484d75bf&searchId=b0edf479bae139c7f8ea1d1d484d75bf&txGid=87a4a3b838c2a1b7cc17e330c7248561&sort=plf-f&originationType=b&rr=
