# Supplementary material for: Vitamin C in Critically Ill Patients: An Updated Systematic Review and Meta-Analysis
Source: Nutrients. 2021 Oct 12;13(10):3564. doi: 10.3390/nu13103564 (PMC8539952; doi:10.3390/nu13103564)
Supplement: Supplementary file 1 [file nutrients-13-03564-s001.zip › Supplementary file S2. Basic study details.pdf]

Supplementary file S2. Basic study details

Vitamin C in critically ill patients: An Updated Systematic Review and Meta-analysis

Dhan Bahadur Shrestha, MD[1], Pravash Budhathoki, MD[2], Yub Raj Sedhai, MD[3*], Sujit Kumar Mandal, MBBS[4], Shreeja Shikhrakar, MBBS[5], Saurab Karki, MBBS[6], Ram Kaji Baniya, MD[7] Markos G. Kashiouris, MD MPH[8], Xian Qiao MD[9], and Alpha A. Fowler, III, MD[8]

[1]*Department of Internal Medicine, Mount Sinai Hospital, Chicago, IL, USA*

[2]*Department of Internal Medicine, Bronxcare Health System, Bronx, NY, USA*

[3]*Department of Internal Medicine, Division of Hospital Medicine, Virginia Commonwealth University, School of Medicine, Richmond, VA, USA*

[4]*Nepalese Army Institute of Health Sciences, Kathmandu, Nepal*

[5]*Kathmandu University School of Medical Sciences, Dhulikhel, Kavrepalanchok, Nepal*

[6]*Military Hospital, Itahari-4, Sunsari, Nepal*

[7]*Our Lady of the Lake Regional Medical Center, Baton Rouge, LA, USA*

[8]*Department of Internal Medicine, Pulmonary and Critical Care, Virginia Commonwealth University, School of Medicine, Richmond, VA, USA*

[9]*Department of Internal Medicine, Pulmonary and Critical Care, Eastern Virginia Medical School Norfolk, VA, USA*

Table S1. Basic details of included studies

ID	Title	Country	Study design	Start date	End date	Inclusion criteria	Exclusion criteria
Abdoulhossein 2018	Effect of vitamin C and vitamin E on lung contusion: A randomized clinical trial study.	Iran	Randomized, double-blind, placebo-controlled clinical trial	Feb-15	June 2018	Patients admitted in ICU surgery with diagnosis of pulmonary contusion due to blunt trauma. Diagnosis of lung contusion: was made by clinically manifestation of respiratory distress, hypoxemia, tachypnea, diminished breath sounds, hypercarbia, chest wall pain and subcutaneous emphysema, at the time of admission. 1-3rib fractures and flail chest, mild to moderate hemothorax, also was detected on plain radiography.	Penetrative trauma, patients in critical condition, complicated patients, needing intubation more than 3 days, requiring emergently major surgical intervention such as brain, abdominal and major orthopedic surgery (pelvic, femur fracture), patients with opium addiction, chronic obstructive pulmonary disease, asthma Glasgow Coma Scale (GCS), less than 13, major associated trauma, pulmonary edema, blood transfusion and thromboembolic effects in long bone fracture, were excluded to minimize confounding the cause of adverse events, any other potential confounder of pure study of lung contusion such as pneumonia, adult respiratory distress syndrome
Ahn 2019	Vitamin C alone does not improve treatment outcomes in mechanically ventilated patients with severe sepsis or septic shock: a retrospective cohort study.	Korea	A retrospective cohort study.	January 2017	July 2017	Those patients who met the criteria for severe sepsis or septic shock and required mechanical ventilation within 24 hours from ICU admission	Age less than 18 years, pregnancy, death within 24 hours from ICU admission, do not resuscitate or limitation of care order, and organ transplantation during ICU stay
Balakrishnan	Hydrocortisone, vitamin C and thiamine for the treatment of sepsis and septic shock following cardiac surgery	India	Double-blinded randomized control study	-	-	Patients diagnosed with septic shock and a procalcitonin (PCT) level >7 ng/mL were included in the study	Septic patients with a PCT level <7 ng/mL within the first 24 h of ICU admission were not eligible for inclusion in the study. Patients<18 years of age, pregnant women, chronic renal failure and immunocompromised were excluded from the study. Patients were considered immunocompromised, if they were taking >10 mg of prednisone-equivalent per day for at least 2 weeks, were receiving cytotoxic therapy or were diagnosed with an acquired immunodeficiency syndrome.
Bedreag 2015	Influence of antioxidant therapy on the clinical status of multiple trauma patients. A retrospective single center study.	Romania	Retrospective study	January 2014	December 2014	The inclusion criteria were: systolic blood pressure (SBP) < 89 mmHg, multiple trauma, and Injury Severity Score (ISS) > 16.	-

Chang K 2020	Adding vitamin C to hydrocortisone lacks benefit in septic shock: a historical cohort study.	Canada	Historical cohort study	December 2016	February 2018	Patients who received at least one dose of hydrocortisone.	Hydrocortisone or vitamin C was administered beginning more than 72 hr after ICU admission, or if it was a readmission. Patients who initially received corticosteroids other than hydrocortisone were excluded
Chang P 2020	Combined Treatment With Hydrocortisone, Vitamin C, and Thiamine for Sepsis and Septic Shock: A Randomized Controlled Trial.	China	Single-center, single-blind, randomized, parallel, controlled trial	September 2017	January 2019	Meeting the diagnostic criteria for Sepsis-3 developed by the American Society of Critical Care Medicine/European Society of Intensive Care Medicine, ≥ 18 years of age, and PCT ≥ 2 ng/mL when entering the ICU	Pregnancy; limitations of care (families discontinued using treatment for sepsis); noninfectious factors, such as severe head injury, uncontrollable major bleeding, cardiogenic shock, advanced tumors, and paraquat poisoning, that may lead to death; and persistent infection sources that cannot be removed by puncture and drainage, debridement, or other surgical procedures.
Collier 2008	Impact of high-dose antioxidants on outcomes in acutely injured patients.	United States	A retrospective cohort study	October 2005	September 2006	all trauma patients admitted from October 1, 2004 to September 30, 2006, 1 year before AO implementation (AO−) and 1 year after AO implementation (AO+)	Patients who were pregnant (admission β-human chorionic gonadotropic) or had a serum creatinine level >2.5 mg/dL
Coloretti 2020	Adjunctive therapy with vitamin c and thiamine in patients treated with steroids for refractory septic shock: A propensity matched before-after, case-control study.	Italy	Propensity matched before-after, case-control study.	January 2015	November 2019	Consecutive adult patients admitted to the polyvalent ICU of the Modena University Hospital with septic shock and receiving low-dose steroids from January 2015 to November 2019	Patients with do-not-resuscitate orders or end of-life decisions during the ICU stay
Dingchao 1994	The protective effects of high-dose ascorbic acid on myocardium against reperfusion injury during and after cardiopulmonary bypass.	China	observational study	-	-	Patients undergoing Cardiopulmonary Bypass (CPB).	-
Du 2003	Therapeutic efficacy of high-dose vitamin C on acute pancreatitis and its potential mechanisms	China	Randomized control study	-	-	Patients diagnosed with acute pancreatitis based on the AP diagnostic criteria worked out by the Pancreatic Surgery Subgroup of Chinese Medical Association	Patients with serious diseases in main visceral organs such as the heart, brain, liver and kidney, peptic ulcer disease, diabetes mellitus, auto-immune diseases, and tumors were excluded.
Emadi 2019	The Effect of High-Dose Vitamin C on Biochemical Markers of	Iran	Double-blind randomiz	-	-	The inclusion criteria were: patients who referred to Shiraz hospitals for undergoing CABG and who signed the	The exclusion criteria were: history of arrhythmias, ejection fraction (EF) < 30%, severe renal or hepatic failure, permanent or temporary pacemaker, treatment with

	Myocardial Injury in Coronary Artery Bypass Surgery.		ed clinical trial			written consent for the participation in the study	antiarrhythmic drugs and digoxin, presence of a degree of atrioventricular block (AV-block) or bradycardia with a rate of < 50 per minute, history of recent myocardial infarction (less than a month), high initial troponin I level, and history of redo or complex (valve replacement + CABG) surgery.
Fowler 2014	Phase I safety trial of intravenous ascorbic acid in patients with severe sepsis.	United States	Randomized double blind placebo-controlled trial	2014		Patients were screened and enrolled following admission to the Medical Respiratory Intensive Care Unit in the VCU Medical Center, Richmond, Virginia. Severe sepsis was defined as: 1) Presence of a systemic inflammatory response: (fever: >38°C or hypothermia: <36°C (core temp only), heart rate > 90 beats/min, leukocytosis: >12,000 WBC/μL or leukopenia: <4,000 WBC/μL or >10% band forms) [23], 2) Suspected or proven infection, and 3) Presence of sepsis induced organ dysfunction: Arterial hypoxemia (PaO2/ FiO2 < 300), systolic blood pressure (SBP) < 90 mm Hg or SBP decrease > 40 mm Hg unexplained by other causes, Lactate > 2.5 mMol/L Urine output < 0.5 ml/kg/hour for greater than two hours despite fluid resuscitation, platelet count < 100,000, acutely developing coagulopathy (INR > 1.5), Bilirubin > 2 mg/dL. If these three criteria were met within 48 hours of ICU admission, informed consent was obtained from family members of patients deemed eligible for the study.	-
Fowler 2019	Effect of Vitamin C Infusion on Organ Failure and Biomarkers of Inflammation and Vascular Injury in Patients With Sepsis and	United States	Randomized, double-blind, placebo-controlle	September 2014	November 2017	Intensive care unit (ICU) admission for sepsis, patients were followed up for development of acute respiratory failure. They were included in CITRIS-ALI if they were undergoing mechanical ventilation through an endotracheal tube,	Patients were excluded if they had a known allergy to vitamin C; there was no ability to obtain informed consent; they were younger than 18 years, non-English speaking, or a ward of the state; more than 48 hours had elapsed since they met ARDS criteria (ie, informed consent was required to occur within 48 hours of the patients' meeting ARDS

	Severe Acute Respiratory Failure: The CITRIS-ALI Randomized Clinical Trial.		d, multicenter trial			had a PaO2 to FiO2 ratio less than 300 mm Hg, had bilateral opacities by chest radiography within 1 week of known clinical insult, had new or worsening respiratory symptoms without evidence of left atrial hypertension, 16 had suspected or proven infection, and met 2 of 4 systemic inflammatory response criteria.17 All criteria had to be met within a 24-hour period.	criteria); they did not have a patient surrogate or physician committed to full support; they were pregnant or breastfeeding; they were moribund and not expected to survive 24 hours; they required home mechanical ventilation (via tracheostomy or noninvasively); they were receiving home oxygen greater than 2 L/min; or they had interstitial lung disease, diffuse alveolar hemorrhage, diabetic ketoacidosis, or an active kidney stone.
Fujii 2020	Effect of Vitamin C, Hydrocortisone, and Thiamine vs Hydrocortisone Alone on Time Alive and Free of Vasopressor Support Among Patients With Septic Shock: The VITAMINS Randomized Clinical Trial.	Australia, New Zealand and Brazil	Randomized controlled trial	May 2018	July 2019	Diagnosis of septic shock<br>All the diagnostic criteria of septic shock (based on the SEPSIS-3 consensus criteria)<br>below has to be fulfilled simultaneously within the last 24 hours, and a vasopressor is infused continuously at the time of enrolment.<br>• Definition of septic shock:<br>Sepsis AND<br>Need for vasopressor therapy to maintain mean arterial pressure<br>>65 mmHg for >2 hours AND<br>Lactate >2 mmol/L, despite adequate fluid resuscitation<br>• Definition of sepsis:<br>Suspected or documented infection AND<br>Acute increase of ≥2 SOFA points consequent to the infection (a<br>proxy of organ dysfunction)	1. Age < 18 years<br>2. Pregnancy<br>3. DNR (do not resuscitate)/DNI (do not intubate) orders<br>4. Death is deemed to be imminent or inevitable during this admission, and either the attending physician, patient or substitute decision-maker is not committed to active treatment<br>5. Patients with known HIV infection<br>6. Patients with known glucose-6 phosphate dehydrogenase (G6PD) deficiency<br>7. Patients transferred from another ICU or hospital with a diagnosis of a septic shock for > 24 hours<br>8. Patients with a diagnosis of a septic shock for > 24 hours<br>9. Patients with known or suspected<br>a. history of oxalate nephropathy or hyperoxaluria<br>b. short bowel syndrome or severe fat-malabsorption<br>c. acute beri-beri disease<br>d. acute Wernicke's encephalopathy<br>e. malaria<br>f. scurvy<br>g. Addison's disease<br>h. Cushing's disease<br>10. Clinician expects to prescribe systemic glucocorticoids for an indication other than septic shock (not including nebulized or inhaled corticosteroid)<br>11. Patient is receiving treatment for systemic fungal infection7 or has documented Strongyloides infection at the time of<br>randomization

							12. Patient with known chronic iron overload due to iron storage and other diseases<br>13. Patient previously enrolled in this study<br>14. Clinician expects to prescribe high dose vitamin C for another indication
Giladi 2011	High-dose antioxidant administration is associated with a reduction in post-injury complications in critically ill trauma patients.	United States	Retrospective cohort study	October 1, 2004	September 30, 2006	All trauma patients admitted from October 1, 2004 to September 30, 2006, 1 year before AO implementation (AO) and 1 year after AO implementation (AO+).	Patients who were pregnant (admission b-human chorionic gonadotropic) or had a serum creatinine greater than 2.5 mg/dL did not receive antioxidants.
Hwang 2020	Combination therapy of vitamin C and thiamine for septic shock: a multi-centre, double-blinded randomized, controlled study	South Korea	Randomized controlled trial	December 2018	January 2020	Adult patients (19–89 years old) who presented to an ED and were diagnosed with septic shock during ED stay	Patients who were transferred from another hospital with vasopressor administration or mechanical ventilator support, patients who had limitations on treatment (e.g., patients with a signed do-not-resuscitate order), patients with an underlying terminal-stage disease; patients taking at least 1 g/day of vitamin C or receiving intravenous thiamine prior to enrolment, patients experiencing cardiac arrest prior to enrolment, patients diagnosed with renal or ureteral stones, patients who met the inclusion criteria more than 24 h after ED arrival, and patients who declined to participate in the trial (directly or by legal proxy)
Iglesias 2020	Outcomes of Metabolic Resuscitation Using Ascorbic Acid, Thiamine, and Glucocorticoids in the Early Treatment of Sepsis: The ORANGES Trial.	USA	Randomized controlled trial	February 2018	April 2019	Adults (≥18 years of age) with a primary diagnosis of sepsis or septic shock according to the 2016 Surviving Sepsis Campaign definitions. Diagnosis of sepsis or septic shock within 12 hours of admission to the ICU and compliance with the 3-hour sepsis bundle.	patients under the age of 18, were pregnant, had a do not resuscitate or do not intubate order on admission, had a terminal end-stage disease (eg, stage IV cancer, end-stage heart failure), did not have a primary admitting diagnosis of sepsis or septic shock, required immediate surgery, had HIV and a CD4 < 50 mm, had known glucose-6 phosphate dehydrogenase deficiency, were transferred from another hospital, or presented with sepsis or septic shock more than 24 hours from admission
Kahn 2011	Resuscitation after severe burn injury using high-dose ascorbic acid: a retrospective review.	United States	Retrospective study	April 2007	August 2009	Patients admitted to the burn/trauma intensive care unit (ICU) between April 2007 and August 2009 with burns more than 20% TBSA were reviewed. Patients were included if they were 18 years or older and had suffered thermal injury less than 10 hours before admission. All patients included were free of major	Patients with >10-hour delay in transfer to the burn center were excluded.

						comorbidities before injury, including chronic obstructive pulmonary disease (COPD), myocardial infarction within 6 months before admission, renal insufficiency (creatinine >1.5 mg/dL), and hepatic or immunologic impairment.	
Kim 2018	Combined vitamin C, hydrocortisone, and thiamine therapy for patients with severe pneumonia who were admitted to the intensive care unit: Propensity score-based analysis of a before-after cohort study.	Korea	Retrospective cohort study	September 2018	August 2019	All consecutive critically ill adult patients with severe pneumonia who were admitted to the medical ICU (12 beds) of a 1100-bed university-affiliated tertiary care hospital in Busan, Korea in June 2016–January 2017 and June 2017–January 2018.	Patients were excluded if they were not admitted to ICU and/or required conventional oxygen therapy only, had an acute diagnosis that was not severe pneumonia, the admission to the ICU occurred N48 h after hospitalization, the vitamin C protocol infusion occurred N48 h after hospitalization, and/or a do not resuscitate order issued.
Lin 2018	High-Dose Ascorbic Acid for Burn Shock Resuscitation May Not Improve Outcomes.	United States	Retrospective case control study	2012	2015	Adult (≥18 years) burn patients admitted to the burn center from 2012 to 2015, who received HDAA during BSR.	We excluded patients from the study who were deemed to have a non-survivable burn injury, which was determined by the admitting burn surgeon on admission.
Litwak 2019	Vitamin C, Hydrocortisone, and Thiamine for the Treatment of Severe Sepsis and Septic Shock: A Retrospective Analysis of Real-World Application.		Retrospective cohort study	October 2016	June 2018	Patients with septic shock admitted to medical, surgical or neurocritical care ICU receiving vasopressor therapy	pregnant females
Long 2020	Early hydrocortisone, ascorbate and thiamine therapy for severe septic shock	United States	Retrospective cohort study	January 1, 2018	May 31, 2019	1)admission to the medical ICU service<br>2) a vasopressor requirement within the first 24 hours of admission with a duration of at least 3 hours<br>3)a treatment plan which included early anti-infective therapy<br>4) no requirement for an open surgical intervention to achieve source control as defined below<br>5)if transferred from a referring hospital, arrival to the ICU occurred within 24	-

						hours of initial presentation to the referring hospital<br>6) initiation of iHAT therapy occurred within 24 hours of admission to the ICU with a full course administered as defined below<br>7) absence of an advanced directive or surrogate decision that limited intensive care therapies within the first 24 hours of ICU admission based on a pre-existing poor prognosis or terminal illness.<br>Patients with sepsis caused by obstructive biliary or urologic pathology necessitating interventional but not open-surgical procedures were included.	
Lv 2020	Early use of high-dose vitamin C is beneficial in treatment of sepsis.	China	Prospective randomized controlled method	June 2017	May 2019	Patients admitted at the ICU department agreed to the treatment plan; new comer in ICU; the age was in the range from18 to75 years; the patients in accord with diagnostic criteria of sepsis; and the patients were not treated with vitamin C prior to admission.	Terminal stage patients; pregnant or lactation period female patients; patients with long-term use of hormones or immunosuppressive agents; patients with malignant tumors under radiotherapy, chemotherapy, or immunotherapy; patients with mental disorders; and patients with autoimmune diseases. The termination and exit criteria were listed as follows: patients with poor compliance who could not cooperate with the doctor; patients with severe adverse events occurred; patients or their family members refused to continue the study; and other reasons for the inability to continue the study.
Marik 2017	Hydrocortisone, Vitamin C, and Thiamine for the Treatment of Severe Sepsis and Septic Shock: A Retrospective Before-After Study.	USA	Retrospective before-after clinical study	January 2016	July 2016	Patients admitted to the Hospital with a primary diagnosis of severe sepsis or septic shock and a procalcitonin (PCT) level ≥2 ng/mL	Septic patients with a PCT level < 2 ng/mL within the first 24 h of ICU admission and and Patients < 18 years of age, pregnant patients, and patients with limitations of care were not treated with the vitamin C protocol.
Masood 2019	Effect of Intravenous Vitamin C, Thiamine, and Hydrocortisone (The Metabolic Resuscitation Protocol) on Early Weaning from Vasopressors in Patients with Septic Shock. A	Pakistan	Descriptive case series	August 2017	April 2018	Men and women 16 to 80 years admitted to ICU with septic shock	Pregnant women or Patients already on vasopressor support for more than 24 hours

	Descriptive Case Series Study.						
Matsuoka 2020	Administration of Corticosteroids, Ascorbic Acid, and Thiamine Improves Oxygenation after Thoracoscopic Esophagectomy.	Japan	Retrospective before-after study	July 2018	December 2018	-Patients undergoing planned thoracoscopic esophagectomy for esophageal carcinoma between July and December 2018	-Patients <20 years of age<br>-Those who received massive transfusions because of excessive intraoperative bleeding -Conversion to open esophagectomy<br>-Those with limitations of care
Mirmohammadsadeghi 2018	Preventive Use of Ascorbic Acid For Atrial Fibrillation After Coronary Artery Bypass Graft Surgery.	Iran	Randomized and double-blinded clinical trial.	-	-	Patients undergoing CABG surgery alone with preoperative sinus rhythm.	Complex surgery, patient disagreement to be involved in trial, emergent surgery, history of cardiac arrhythmia or antiarrhythmic drugs, and severe renal or hepatic failure.
Mitchell 2020	Vitamin C and Thiamine for Sepsis and Septic Shock.	United States	Retrospective study	March 2017	July 2018	Between March 2017 and July 2018, patients admitted to the medical or surgical ICU who received IV vitamin C, thiamine, and hydrocortisone were included as the treatment group. The control group were patients admitted between January 2015 and December 2016 who received IV hydrocortisone alone. Patients had a diagnosis of either sepsis or septic shock defined by the 2012 or 2016 Surviving Sepsis Guidelines.	-
Mohamed 2020	Vitamin C Therapy for Routine Care in Septic Shock (ViCTOR) Trial: Effect of Intravenous Vitamin C, Thiamine, and Hydrocortisone Administration on Inpatient Mortality among Patients with Septic Shock.	India	Randomized Controlled Trial	June 2018	August 2019	All patients admitted to the participating intensive care units (ICUs) of the tertiary referral teaching hospital were identified by an alert from the primary physician/nurse. Adult non-pregnant patients with septic shock (Surviving Sepsis Campaign 2016 guidelines) and within 6 hours of initiation of inotropic support	Patients with burns, limitations of care due to terminal illness or acute liver failure
Moskowitz 2020	Effect of Ascorbic Acid, Corticosteroids, and Thiamine on Organ Injury in Septic Shock:	United States	Randomized, blinded, multicent	February 9, 2018	November 26, 2019.	Adult patients (aged ≥18 years) were eligible if they had a suspected or confirmed infection and were receiving a vasopressor because of sepsis. Patients	Allergic to study drug components, had a clinical indication for any of the study drugs, had symptomatic kidney stones within the last year, had glucose-6-phosphate dehydrogenase deficiency or hemochromatosis,

	The ACTS Randomized Clinical Trial.		er clinical trial.			were enrolled within 24 hours once they were identified as meeting inclusion criteria.	were receiving kidney replacement therapy (changed from stage 3b chronic kidney disease after the 19th enrolled patient because of difficulty ascertaining chronic kidney disease stage), were not expected to sur-vive 24 hours, or were a member of a protected population(ie, pregnant, prisoner).
Nagel 2020	Safety, Pharmacodynamics, and Efficacy of High- Versus Low-Dose Ascorbic Acid in Severely Burned Adults.	Germa ny	Retrospe ctive before-and-after-study.	July 2016	January 2018	Patients admitted immediately after burn trauma, legal age (>18 years)	Iron storage disease like hemochromatosis or thalassemia
Nakaji ma 2019	Effect of high-dose vitamin C therapy on severe burn patients: a nationwide cohort study.	Japan	Cohort study	July 2010	March 2016	Aged≥15 years with burn index≥15 were included, patients with first admission were included.	Patients discharged within 1 day after admission (to avoid immortal time bias), patients readmitted patients were excluded from the study.
Nathen s 2002	Randomized, prospective trial of antioxidant supplementation in critically ill surgical patients.	United States	Randomi zed, prospecti ve study	February 1999	June 2000	16 to 74 years old, were admitted to the ICU under the general surgery/trauma service, and were available for enrollment within 24 hours of sustaining their injury (in the case of trauma patients) or undergoing an emergency operation.	Patients with isolated or severe (Glasgow Coma Scale score 6 or less), head injury, brain death, anticipated survival less than 48 hours, burns over more than 20% body surface area, sickle cell anemia, need for anticoagulation with Coumadin while in the ICU, and chronic renal failure (creatinine2.5 mg/dL).
Palli 2017	The impact of N-acetylcysteine and ascorbic acid in contrast-induced nephropathy in critical care patients: an open-label randomized controlled study.	Greece	One-center, two-arm, randomiz ed, open-label, controlle d trial.	2010	2013	Inclusion criteria were age ≥14 years and diagnostic need for contrast-enhanced CT.	Exclusion criteria were history of intravascular administration of contrast agent during the 6-day period prior to randomization, the use of antioxidant agents during the last week before the examination, unstable renal function, use of RRT in the 3-day period before randomization and pregnancy. Unstable renal function defined as a change in serum creatinine values greater than 20% between 2 consecutive days during the 3 days prior to randomization, independently from crude baseline renal function.
Park 2020	Impact of Vitamin C and Thiamine Administration on Delirium-Free Days in Patients with Septic Shock	Korea	Retrospe ctive study	January 2017	July 2018	(1) ≥18 years; (2) diagnosed with septic shock during their ED stay and admitted to the ICU from the ED	Patients who were transferred to the general ward, signed a “do not attempt resuscitation order”, were discharged to home, or expired in the ED
Reddy 2020	Metabolic Resuscitation Using Hydrocortisone, Ascorbic Acid, and	India	Single center, prospecti	-	-	Patients admitted to intensive care unit (ICU) with diagnosis of septic shock and age more than 18 years are included in	pregnant patients and those with new onset acute coronary syndrome along with sepsis

	Thiamine: Do Individual Components Influence Reversal of Shock Independently?		ve, randomized nonblinded clinical trial			the study.<br>A rise in SOFA score of 2 with persistent hemodynamic instability despite fluid resuscitation and requiring vasopressors criteria should be met.	
Sadaka 2020	Ascorbic Acid, Thiamine, and Steroids in Septic Shock: Propensity Matched Analysis.	USA	Retrospective Cohort Study	March 2017	September 2018	18 years of age or older, had a principal diagnosis of SS as defined by the Surviving Sepsis Campaign (Sepsis 3) definition, that is, patients with sepsis who developed systolic blood pressure less than 90 mm Hg, not responding to appropriate fluid resuscitation, and requiring VP to maintain mean arterial pressure greater than or equal to 65 mm Hg	
Sadeghpour 2015	Impact of vitamin C supplementation on post-cardiac surgery ICU and hospital length of stay.	Iran	Randomized controlled trial			Age ≥ 18 years with American Society of Anesthesiologists (ASA) physical status class II-III and candidacy for coronary artery bypass graft operation (CABG) or simple congenital valvular disease surgery	Patients who died within the first postoperative day and those who had not received adequate doses of drugs according to the protocol. Patients with complications (cardiac, respiratory or neurological) or emergent operation.
Sandesc 2018	Analysis of oxidative stress-related markers in critically ill polytrauma patients: An observational prospective single-center study.	Austria	Prospective Study	January 2014	December 2015	Injury Severity Score (ISS) > 16 and age > 18 years.	
Shin 2019	Early Vitamin C and Thiamine Administration to Patients with Septic Shock in Emergency Departments: Propensity Score-Based Analysis of a Before-and-After Cohort Study.	Korea	Propensity Score-Based Analysis of a Before-and-After Cohort Study	July 2017	December 2017	All adult patients (age ≥ 19 years) with septic shock who were diagnosed in an ED were enrolled prospectively since October 2015. Hospital A participated in this registry from the beginning, and Hospital B started enrolling patients in January 2016. Septic shock was defined as refractory hypotension requiring vasopressors despite adequate fluid therapy (20–30 mL/kg crystalloid	Patients were excluded if they were in a “Do Not Attempt Resuscitation” state; if septic shock was recognized 6 hours after arrival in the ED; if they were transferred from other hospitals and did not meet the inclusion criteria on ED arrival; or if they were transferred directly from the ED to other hospitals

						solution), or hypoperfusion, which was defined as a blood lactate concentration ≥4 mmol/L in patients with suspected or confirmed infection.	
Siriwardena 2007	Randomised, double blind, placebo controlled trial of intravenous antioxidant (n-acetylcysteine, selenium, vitamin C) therapy in severe acute pancreatitis	England	Randomised, double blind, placebo controlled trial	June 2001	November 2004	Patients were included whose clinical and biochemical presentation was consistent with acute pancreatitis (specifically, a history of acute abdominal pain associated with a greater than threefold elevation of the serum amylase and/or computed tomographic evidence of acute pancreatitis); with predicted severe acute pancreatitis defined for the purposes of this study as an APACHE II score of 8 or more either at admission or within 48 hours of admission. Inclusion criteria stipulated that patients be enrolled within 72 hours of admission to hospital and that they be 16 years of age or over, not currently enrolled in another trial, without a history of allergy to intravenous antioxidant therapy and able to give written informed consent.	
Vail 2020	Use of Hydrocortisone, Ascorbic Acid, and Thiamine in Adults with Septic Shock.	USA	Retrospective Cohort Study	October 2015	June 2018	Adult patients with septic shock discharged from participating hospitals between October 1, 2015, when International Classification of Disease, 10th edition (ICD-10) coding was adopted in the US, and June 30, 2018.	Patients <18 years old, never admitted to an ICU, or had diagnoses that might result in IV ascorbic acid administration for other reasons (vitamin C deficiency and burn injury). Patients hospitalized during December 2016 when results of the Marik study were first published. hospitals with <6 admissions with septic shock in each quarter year
Wang 2020	Effect of Intravenous Injection of Vitamin C on Postoperative Pulmonary Complications in Patients Undergoing Cardiac Surgery: A Double-Blind, Randomized Trial.	China	Prospective, double-blind, randomized con-trolled trial	December 2018	September 2019	Aged 18–65 years with American Society of Anesthesiologists (ASA) physical status II or III who were scheduled for cardiac surgery under CPB at Affiliated Hospital of Xuzhou Medical University.	Patients were excluded for the following reasons: were scheduled for emergency surgery; had a history of cardiac surgery; were allergic to ascorbic acid; had gout, high oxalate urine, uric acid kidney stone, glucose-6-phosphate dehydrogenase deficiency, hemochromatosis, sickle cell anemia, iron myeloblastic anemia, or thalassemia; had a preoperative chest radiograph showing active lung disease; or had a pulmonary arterial systolic blood pressure >60 mmHg. Patients with operation time exceeding 6 hours, withdrawing informed consent, died of

							non-respiratory diseases during hospitalization are eliminated.
Wani 2020	Combination of vitamin C, thiamine and hydrocortisone added to standard treatment in the management of sepsis: results from an open label randomised controlled clinical trial and a review of the literature.	India	Randomised controlled trial	April 2018	June 2019	Patients who were admitted with a diagnosis of sepsis and septic shock with a serum lactate level of >2 mmol/l were enrolled in the study.<br>The diagnosis of sepsis and septic shock was based on The Third International Consensus Definitions for Sepsis and Septic Shock, where in sepsis is defined as a life-threatening organ dysfunction caused by a dysregulated host response to infection. Organ dysfunction is represented by an increase in the SOFA score of 2 points or more; and septic shock is a subset of sepsis who fulfill the criteria for sepsis and, despite adequate fluid resuscitation, require vasopressors to maintain a mean arterial pressure (MAP) 65 mmHg and have a lactate >2 mmol/L(>18 mg/dL).	Patients aged less than 18 years and pregnant patients.
Yanase 2020	A Pilot, Double-Blind, Randomized, Controlled Trial of High-Dose Intravenous Vitamin C for Vasoplegia After Cardiac Surgery.	Australia and New Zealand	Prospective, double-blind, randomized, controlled trial.	November 8, 2017	October 29, 2018	Adult patients (≥18 years of age) who underwent on-pump cardiac surgery, admitted to the intensive care unit (ICU), and met the study enrollment criteria for postoperative vasoplegia. We included patients only when they met inclusion criteria and could be randomized within six hours after ICU admission. We defined postcardiac surgery vasoplegia as hypotension with normal or increased cardiac index (CI), and a low systemic vascular resistance. We operatively defined vasoplegia by the above criteria and the need for any dose of continuous vasopressor infusion to a maintain mean arterial pressure (MAP) > 65 mmHg, in the setting of a CI ≥ 2.2 Lmin-1m-2and/or of a central venous oxygen saturation > 60%.	Pregnancy, the use of vasopressor or inotropic drugs in the preoperative period, off-pump cardiac surgery, corticosteroids use prior or after surgery, a history of oxalate nephropathy, haemochromatosis, and glucose 6 phosphate dehydrogenase deficiency, if the treating clinician believed there was an additional cause for hypotension other than vasoplegia (bleeding, fluid requirement, pneumothorax, pacemaker issues, heart failure or infection).

Yoo 2020	Clinical Impact of Supplementation of Vitamins B1 and C on Patients with Sepsis-Related Acute Respiratory Distress Syndrome.	Korea	Retrospective Cohort Study	June 2017	May 2019	Patients older than 18 year olds with sepsis-related ARDS who received IMV and admitted at medical ICU (MICU). All patients under IMV met the Berlin criteria for ARDS.	-
Zabet 2016	Effect of high-dose Ascorbic acid on vasopressor's requirement in septic shock.	Iran	Randomized Controlled Trial	September 2014	January 2016	Adult (18–65-year-old) surgical critically ill patients with diagnosis of septic shock who needed a vasopressor drug to maintain mean arterial pressure (MAP) >65 mmHg despite adequate fluid resuscitation	Concomitant use of other antioxidants (such as Vitamin E, selenium, and N-acetylcysteine), corticosteroids administration, any contraindication for high-dose ascorbic acid including bilateral ureteric obstruction, chronic hemodialysis, iron overload, oxalate stone formers, hemochromatosis, and glucose-6-phosphate dehydrogenase deficiency
Ferron Celma 2011	Effect of Vitamin C Administration on Neutrophil Apoptosis in Septic Patients After Abdominal Surgery	Spain	A prospective, randomized, double-blinded clinical trial			Patients undergoing abdominal surgery in Digestive Surgery Department who had a physiological and operative severity score for the enumeration of mortality and morbidity (POSSUM) score indicating a postoperative mortality risk of >30%	
Nabil-Habib 2017	Early Adjuvant Intravenous Vitamin C Treatment in Septic Shock may Resolve the Vasopressor Dependence	Egypt	Prospective controlled study			Adult patients admitted to the critical care department with the diagnosis of septic shock	Pregnant and lactating mothers were excluded.<br>Any patients with a history of oxalate nephrolithiasis or in documented glucose6-phosphate dehydrogenase G6PD deficiency, paroxysmal nocturnal hemoglobinuria, and hereditary hemochromatosis were excluded.<br>Any patient developed any other type of shock state or patients with mixed type of shock were excluded.
Tanaka 2000	Reduction of resuscitation fluid volumes in severely burned patients using ascorbic acid administration: a randomized, prospective study.	Japan	Prospective Randomized Study	December 1992	December 1997	Older than 16 years, thermal injury within 2 hours before admission, burn covering more than 30% of TBSA<br>no pre-existing hepatic, respiratory, cardiac or renal dysfunction, and no pre-existing coagulopathy	

Galley 1997	The effects of intravenous antioxidants in patients with septic shock.	UK	Randomized Controlled Trial			patients in intensive care unit with septic shock, as soon as they fulfilled the criteria for septic shock	
Razmkon 2011	Administration of vitamin C and vitamin E in severe head injury: A randomized double-blind controlled trial	Iran	Randomized, double-blind, placebo-controlled trial			All adult patients (age $ 16 years) with severe head injury, as defined by a Glasgow Coma Scale score of less than or equal to 8, with the radiologic diagnosis of diffuse axonal injury	Patients with significant liver or renal disease, or glucose-6-phosphate dehydrogenase deficiency. Patients with previous significant CNS pathology (e.g., stroke, head injury, or previous craniotomy) affecting measurements on imaging. Patients with space-occupying lesions necessitating emergency evacuation and those with pre resuscitation shock and hypoxic ischemic encephalopathy.
Bansal, et al. 2011	Safety and Efficacy of Vitamin-based Antioxidant Therapy in Patients with Severe Acute Pancreatitis	India	A Randomized Controlled Trial	April 2005	May 2006	adult patients of either gender between 18 and 75 years of age presenting to the medical emergency unit, and whose clinical and biochemical presentation was consistent with AP (specifically, a history of acute abdominal pain associated with a greater than threefold elevation of the serum amylase and/or lipase >3 times normal, along with CT evidence of AP). Severe AP was defined for the purposes of this study as an APACHE II score of 8 or more at admission and CT severity index (CTSI) ≥7. Only those patients presenting with a first attack or an acute exacerbation of chronic pancreatitis was enrolled. The patients were enrolled within 96 hours of onset of the symptoms. All the criteria had to be present before the patient could be randomized.	Exclusion criteria were: age <18 or >75 years; pregnancy; AP secondary to surgery, trauma, or malignancy; psychosis (except alcoholic delirium); need for urgent therapeutic intervention (endoscopic papillotomy, cholecystectomy, and/or choledochotomy); those enrolled in any other trial; patients with serious diseases of the heart, brain, liver, or kidney; peptic ulcer; autoimmune disease.
Sevransky, et al 2021	Effect of Vitamin C, Thiamine, and Hydrocortisone on Ventilator- and Vasopressor-Free Days in Patients With Sepsis	43 hospitals in the United States	Multicenter, randomized, double-blind,, placebo-	August 2018	July 2019	Adult patients with sepsis-induced respiratory and/or cardiovascular dysfunction	Underpowered to show clinically meaningful differences being stopped early. Open label use of corticosteroids Biological heterogeneity due to diverse group of patients

	The VICTAS Randomized Clinical Trial		controlle d trial				
