# Supplementary material for: Vitamin C in Critically Ill Patients: An Updated Systematic Review and Meta-Analysis
Source: Nutrients. 2021 Oct 12;13(10):3564. doi: 10.3390/nu13103564 (PMC8539952; doi:10.3390/nu13103564)
Supplement: Supplementary file 1 [file nutrients-13-03564-s001.zip › Supplementary file S3. Additional analysis.pdf]

Supplementary file S3. Additional analysis

Vitamin C in critically ill patients: An Updated Systematic Review and Meta-analysis

Dhan Bahadur Shrestha, MD[1], Pravash Budhathoki, MD[2], Yub Raj Sedhai, MD[3*], Sujit Kumar Mandal, MBBS[4], Shreeja Shikhrakar, MBBS[5], Saurab Karki, MBBS[6], Ram Kaji Baniya, MD[7] Markos G. Kashiouris, MD MPH[8], Xian Qiao MD[9], and Alpha A. Fowler, III, MD[8]

[1]*Department of Internal Medicine, Mount Sinai Hospital, Chicago, IL, USA*

[2]*Department of Internal Medicine, Bronxcare Health System, Bronx, NY, USA*

[3]*Department of Internal Medicine, Division of Hospital Medicine, Virginia Commonwealth University, School of Medicine, Richmond, VA, USA*

[4]*Nepalese Army Institute of Health Sciences, Kathmandu, Nepal*

[5]*Kathmandu University School of Medical Sciences, Dhulikhel, Kavrepalanchok, Nepal*

[6]*Military Hospital, Itahari-4, Sunsari, Nepal*

[7]*Our Lady of the Lake Regional Medical Center, Baton Rouge, LA, USA*

[8]*Department of Internal Medicine, Pulmonary and Critical Care, Virginia Commonwealth University, School of Medicine, Richmond, VA, USA*

[9]*Department of Internal Medicine, Pulmonary and Critical Care, Eastern Virginia Medical School Norfolk, VA, USA*

1. **Overall hospital mortality**

1.1. Among RCTs

a. **Based on patient group:**

Neither among patients in sepsis/septic shock (OR, 0.98; 95% CI, 0.76-1.26; n= 1297; $I^2 = 0\%$; p=0.86) nor among surgical patients (OR, 1.08; 95% CI, 0.63- 1.86; n= 860; $I^2 = 4\%$) significant differences for hospital mortality was observed comparing vitamin C and placebo or SOC groups (**Figure S1**).

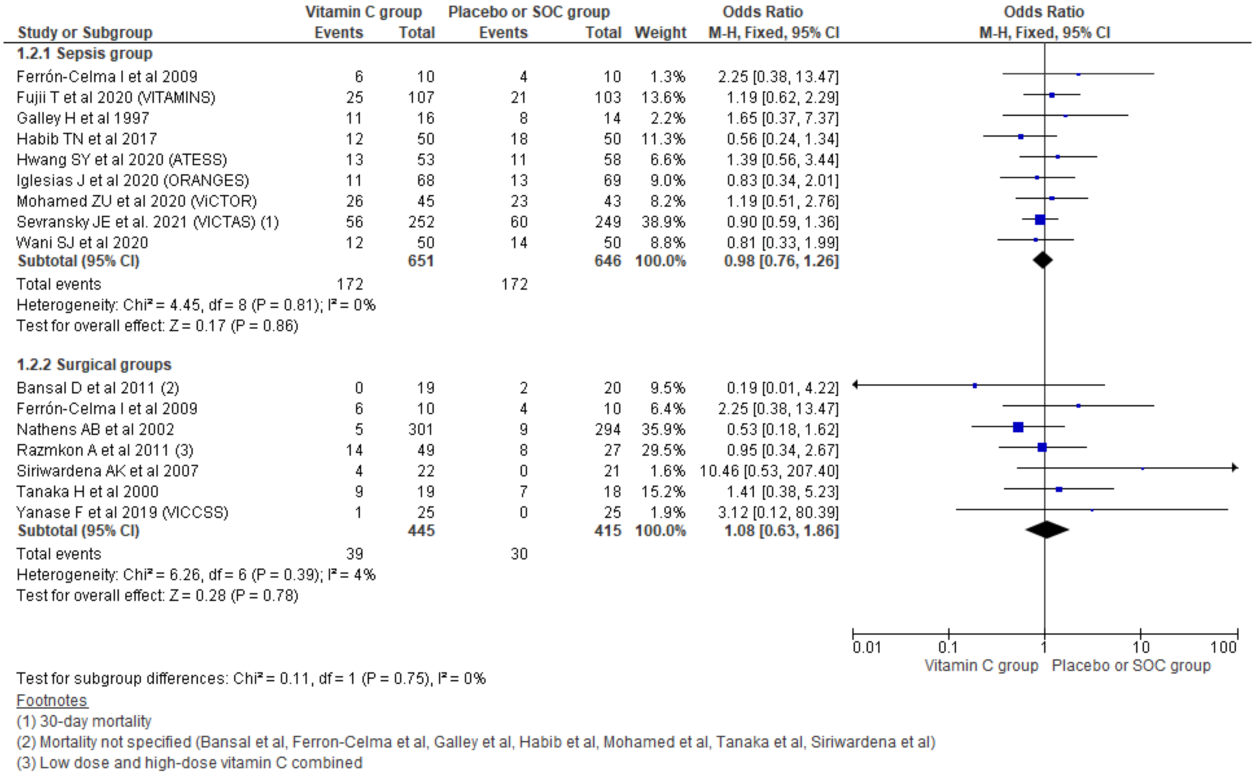

Figure S1. Forest plot showing hospital mortality outcome across surgical and sepsis group among RCTs

b. **Based on blinding**

Running analysis for hospital mortality taking RCTs based on blinding of trials also could not show significant differences between two groups. For double blinded trials, odds for mortality was showed to be slightly higher among vitamin C group (OR, 1.04; 95% CI, 0.76-1.43; n= 938; I^2 = 0%; p=0.80) while for single blinded/open odds was slightly reduced (OR 0.92, 95% CI 0.65 to 1.29; n= 1199; I^2 = 0%; p=0.61) comparing with placebo or SOC group; but it could not reach statistical significance (**Figure S2**).

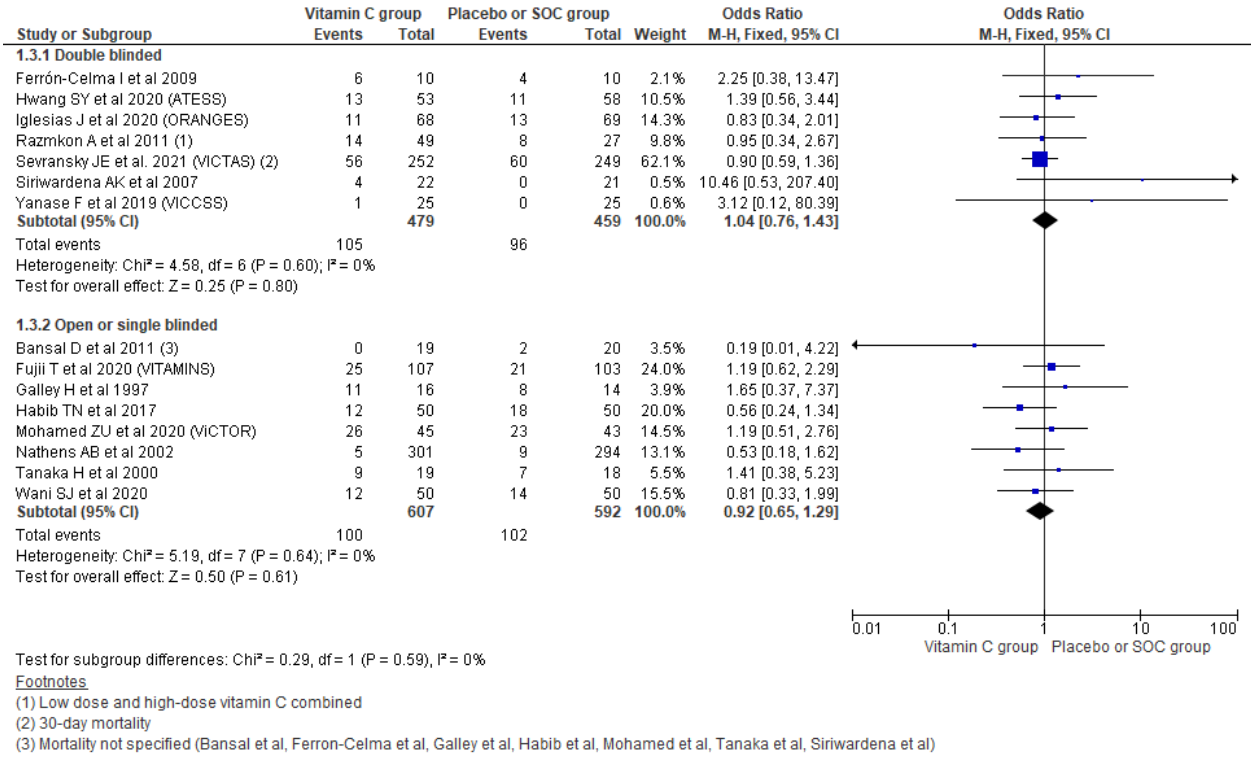

Figure S2. Forest plot showing hospital mortality outcome across double blinded and single blinded/open RCTs

c. Based on concomitant other anti-oxidants use

Analysis comparing placebo/ SOC with vitamin C only (OR, 0.93; 95% CI, 0.49-1.76; n= 207; I^2 = 5%; p=0.82) and placebo/ SOC with other concomitant anti-oxidants use with vitamin C (OR,

0.99; 95% CI, 0.77- 1.27; n= 1930; I^2 = 0%; p=0.94) also could not show significant differences in hospital mortality (**Figure S3**).

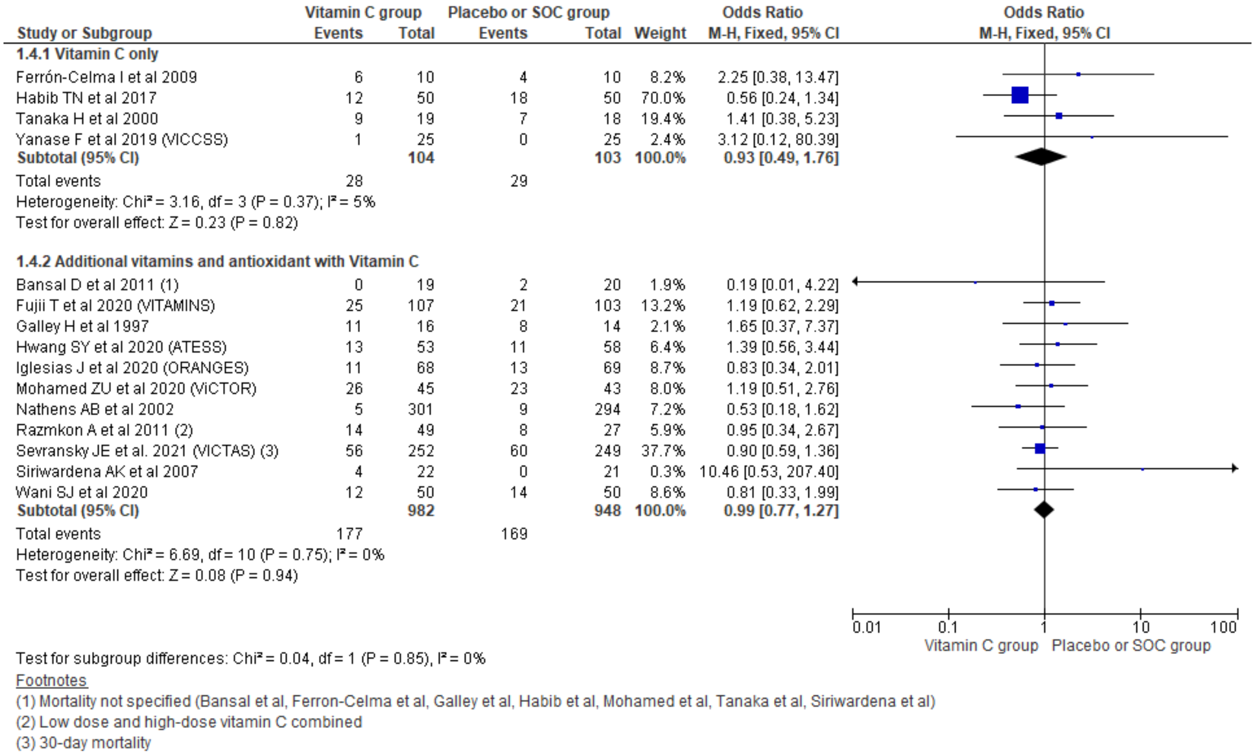

Figure S3. Forest plot showing hospital mortality outcome across use of vitamin C only or other anti-oxidants with vitamin C as treatment group across RCTs

1.2. Among observational studies

a. Based on patient group:

In sepsis/septic shock group mortality could not reach significant differences (OR, 0.73; 95% CI, 0.46-1.15; n= 340351; I^2 = 86%; p=0.17) while among surgical patients (OR, 0.69; 95% CI, 0.56-0.85; n= 5143; I^2 = 4%; p=0.0005) significant reduction in hospital mortality was observed comparing vitamin C and placebo/ SOC groups (**Figure S4**).

Study or Subgroup	Vitamin C group Events	Vitamin C group Total	Placebo or SOC group Events	Placebo or SOC group Total	Weight	Odds Ratio M-H, Random, 95% CI
**1.5.1 Sepsis group**						
Ahn JH et al 2019	16	35	16	40	7.9%	1.26 [0.50, 3.16]
Chang K et al 2020	20	52	32	88	9.1%	1.09 [0.54, 2.22]
Coloretti I et al 2020	28	56	34	56	8.8%	0.65 [0.31, 1.37]
Kim WY et al 2018 (1)	6	36	14	36	6.9%	0.31 [0.10, 0.95]
Litwak JJ et al 2019	19	47	19	47	8.4%	1.00 [0.44, 2.28]
Long MT et al 2020	21	79	41	127	9.5%	0.76 [0.41, 1.42]
Marik PE et al 2017	4	47	19	47	6.5%	0.14 [0.04, 0.45]
Mitchell AB et al 2019	18	38	13	38	7.8%	1.73 [0.69, 4.36]
Sadaka F et al 2019	9	31	14	31	7.2%	0.50 [0.17, 1.42]
Shin TG et al 2019	38	227	97	527	10.6%	0.89 [0.59, 1.35]
Vail EA et al 2020	1459	3574	92060	335023	11.6%	1.82 [1.70, 1.95]
Yoo JW et al 2020	19	33	33	36	5.7%	0.12 [0.03, 0.48]
**Subtotal (95% CI)**		**4255**		**336096**	**100.0%**	**0.73 [0.46, 1.15]**
Total events	1657		92392			
Heterogeneity: Tau² = 0.46; Chi² = 75.88, df = 11 (P < 0.00001); I² = 86%						
Test for overall effect: Z = 1.36 (P = 0.17)						
**1.5.2 Surgical group**						
Bedreag OH et al 2015 (2)	6	20	9	14	2.1%	0.24 [0.06, 1.02]
Giladi AM et al 2011	139	2272	172	2022	62.7%	0.70 [0.56, 0.88]
Kahn SA et al 2011	4	17	3	16	1.6%	1.33 [0.25, 7.17]
Lin J et al 2018	10	38	10	42	4.3%	1.14 [0.42, 3.15]
Nakajima M et al 2019 (3)	58	127	278	508	26.3%	0.70 [0.47, 1.03]
Sandesc M et al 2018	5	35	11	32	3.1%	0.32 [0.10, 1.05]
**Subtotal (95% CI)**		**2509**		**2634**	**100.0%**	**0.69 [0.56, 0.85]**
Total events	222		483			
Heterogeneity: Tau² = 0.00; Chi² = 5.23, df = 5 (P = 0.39); I² = 4%						
Test for overall effect: Z = 3.47 (P = 0.0005)						

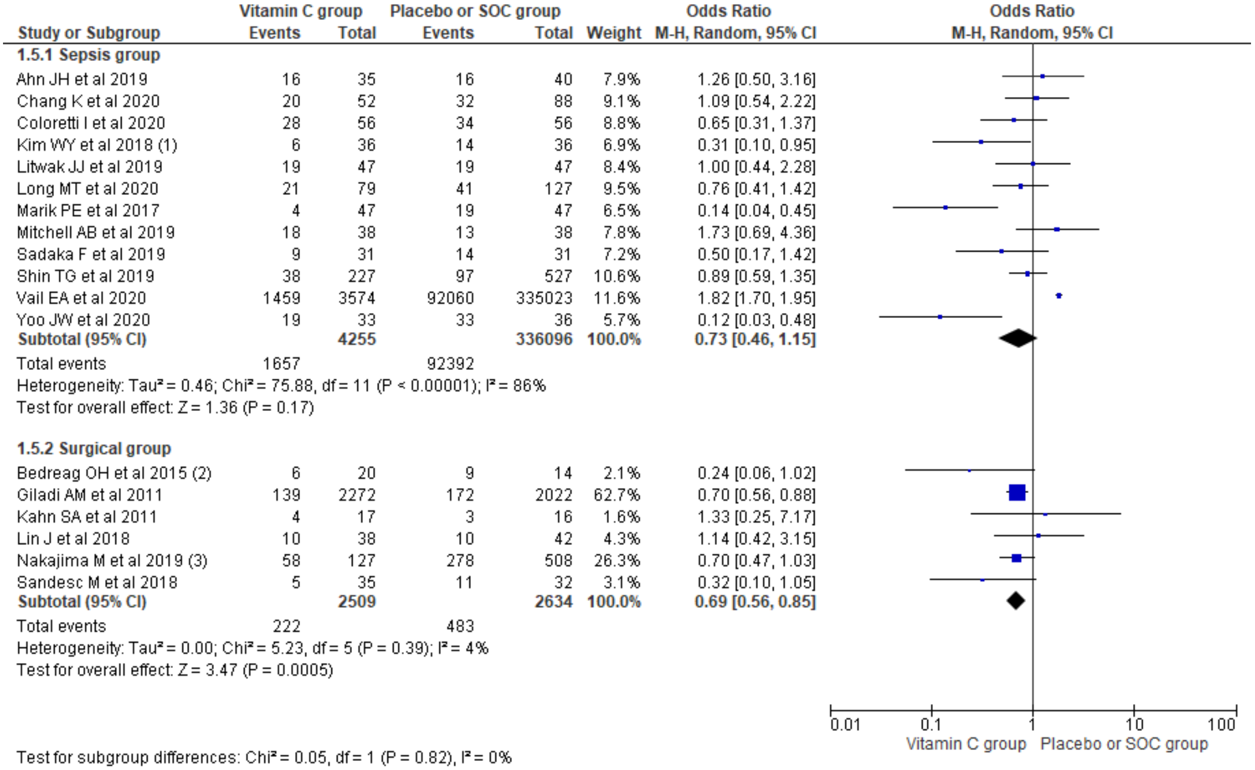

Test for subgroup differences: Chi² = 0.05, df = 1 (P = 0.82), I² = 0%

Footnotes
(1) Among matched cohort (Kim et al, Shin et al)
(2) Mortality not specified (Bedreag et al, Kahn et al, Lin et al, Sandesc et al)
(3) 24 g threshold of high dose Vitamin C

Figure S4. Forest plot showing hospital mortality outcome across surgical and sepsis group among observational studies

b. Based on concomitant other anti-oxidants use

Analysis comparing placebo/ SOC with vitamin C only could not reach significant differences in mortality (OR, 0.86; 95% CI, 0.64-1.16; n= 963; $I^2 = 0\%$; p=0.32); while comparing placebo/ SOC with other concomitant anti-oxidants use with vitamin C showed significant reduction in hospital mortality (OR, 0.60; 95% CI, 0.38-0.95; n= 344531; $I^2 = 91\%$; p<0.0001) (**Figure S5**).

Study or Subgroup	Vitamin C group Events	Vitamin C group Total	Placebo or SOC group Events	Placebo or SOC group Total	Weight	Odds Ratio M-H, Random, 95% CI
**1.6.1 Vitamin C only**						
Ahn JH et al 2019	16	35	16	40	10.7%	1.26 [0.50, 3.16]
Chang K et al 2020	20	52	32	88	18.0%	1.09 [0.54, 2.22]
Kahn SA et al 2011	4	17	3	16	3.2%	1.33 [0.25, 7.17]
Lin J et al 2018	10	38	10	42	8.8%	1.14 [0.42, 3.15]
Nakajima M et al 2019 (1)	58	127	278	508	59.2%	0.70 [0.47, 1.03]
**Subtotal (95% CI)**		**269**		**694**	**100.0%**	**0.86 [0.64, 1.16]**
Total events	108		339			
Heterogeneity: Tau² = 0.00; Chi² = 2.82, df = 4 (P = 0.59); I² = 0%						
Test for overall effect: Z = 1.00 (P = 0.32)						
**1.6.2 Additional vitamins and antioxidant with Vitamin C**						
Bedreag OH et al 2015 (2)	6	20	9	14	5.2%	0.24 [0.06, 1.02]
Coloretti I et al 2020	28	56	34	56	8.3%	0.65 [0.31, 1.37]
Giladi AM et al 2011	139	2272	172	2022	10.4%	0.70 [0.56, 0.88]
Kim WY et al 2018 (3)	6	36	14	36	6.6%	0.31 [0.10, 0.95]
Litwak JJ et al 2019	19	47	19	47	7.9%	1.00 [0.44, 2.28]
Long MT et al 2020	21	79	41	127	8.9%	0.76 [0.41, 1.42]
Marik PE et al 2017	4	47	19	47	6.3%	0.14 [0.04, 0.45]
Mitchell AB et al 2019	18	38	13	38	7.4%	1.73 [0.69, 4.36]
Sadaka F et al 2019	9	31	14	31	6.9%	0.50 [0.17, 1.42]
Sandesc M et al 2018	5	35	11	32	6.2%	0.32 [0.10, 1.05]
Shin TG et al 2019	38	227	97	527	9.8%	0.89 [0.59, 1.35]
Vail EA et al 2020	1459	3574	92060	335023	10.6%	1.82 [1.70, 1.95]
Yoo JW et al 2020	19	33	33	36	5.5%	0.12 [0.03, 0.48]
**Subtotal (95% CI)**		**6495**		**338036**	**100.0%**	**0.60 [0.38, 0.95]**
Total events	1771		92536			
Heterogeneity: Tau² = 0.52; Chi² = 140.31, df = 12 (P < 0.00001); I² = 91%						
Test for overall effect: Z = 2.19 (P = 0.03)						

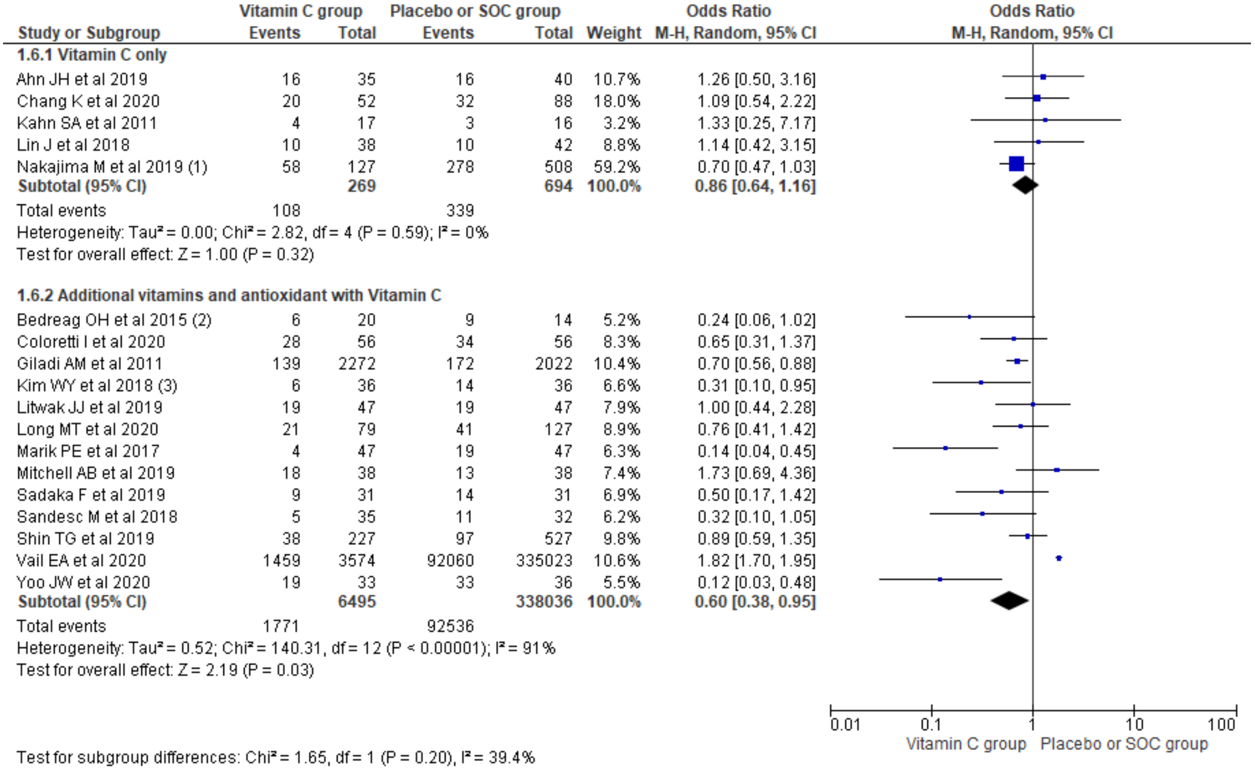

Test for subgroup differences: Chi² = 1.65, df = 1 (P = 0.20), I² = 39.4%

Footnotes
(1) 24 g threshold of high dose Vitamin C
(2) Mortality not specified (Bedreag et al, Kahn et al, Lin et al, Sandesc et al)
(3) Among matched cohort (Kim et al, Shin et al)

Figure S5. Forest plot showing hospital mortality outcome across use of vitamin C only or other anti-oxidants with vitamin C as treatment group across observational studies

2. 28/30-day mortality

Pooling data for 28/30-days mortality using random effect model also showed similar findings as in fixed effect model with no significant group differences in overall 28/30-days mortality (OR, 0.86; 95% CI, 0.69-1.07; n= 3405; I^2 = 28%; p=0.18); among RCTs (OR, 0.77; 95% CI, 0.57-1.05; n= 2131; I^2 = 37%; p=0.10); observational studies (OR, 1.00; 95% CI, 0.75-1.33; n= 1274; I^2 = 4%; p=0.98) (**Figure S6**).

Study or Subgroup	Vitamin C group Events	Vitamin C group Total	Placebo or SOC group Events	Placebo or SOC group Total	Weight	Odds Ratio M-H, Random, 95% CI
**2.1.1 RCTs**						
Chang P et al 2020 (HYVCTTSSS)	11	40	14	40	4.3%	0.70 [0.27, 1.82]
Fowler AA et al 2014 (1)	7	16	5	8	1.5%	0.47 [0.08, 2.66]
Fowler AA et al 2019 (CITRIS-ALI)	25	84	38	82	7.8%	0.49 [0.26, 0.93]
Fujii T et al 2020 (VITAMINS)	24	106	21	103	7.4%	1.14 [0.59, 2.21]
Hwang SY et al 2020 (ATESS)	11	53	9	58	4.2%	1.43 [0.54, 3.77]
Lv SJ et al 2020	15	61	24	56	5.8%	0.43 [0.20, 0.96]
Moskowitz A et al 2020 (ACTS)	35	101	29	99	8.5%	1.28 [0.71, 2.32]
Nathens AB et al 2002	4	301	7	294	2.8%	0.55 [0.16, 1.91]
Sevransky JE et al. 2021 (VICTAS)	56	252	60	249	12.5%	0.90 [0.59, 1.36]
Wani SJ et al 2020	20	50	21	50	5.7%	0.92 [0.41, 2.04]
Zabet MH et al 2016	2	14	9	14	1.3%	0.09 [0.01, 0.59]
**Subtotal (95% CI)**		**1078**		**1053**	**61.6%**	**0.77 [0.57, 1.05]**
Total events	210		237			
Heterogeneity: Tau² = 0.09; Chi² = 15.82, df = 10 (P = 0.10); I² = 37%						
Test for overall effect: Z = 1.65 (P = 0.10)						
**2.1.2 Observational studies**						
Ahn JH et al 2019	12	35	8	40	3.7%	2.09 [0.74, 5.92]
Coloretti I et al 2020	24	56	28	56	6.3%	0.75 [0.36, 1.58]
Mitchell AB et al 2019	22	38	23	38	4.6%	0.90 [0.36, 2.24]
Park JE et al 2020 (2)	21	89	16	89	6.5%	1.41 [0.68, 2.92]
Shin TG et al 2019	42	227	97	527	12.9%	1.01 [0.67, 1.50]
Yoo JW et al 2020	18	33	32	46	4.5%	0.53 [0.21, 1.33]
**Subtotal (95% CI)**		**478**		**796**	**38.4%**	**1.00 [0.75, 1.33]**
Total events	139		204			
Heterogeneity: Tau² = 0.01; Chi² = 5.24, df = 5 (P = 0.39); I² = 4%						
Test for overall effect: Z = 0.02 (P = 0.98)						
**Total (95% CI)**		**1556**		**1849**	**100.0%**	**0.86 [0.69, 1.07]**
Total events	349		441			
Heterogeneity: Tau² = 0.06; Chi² = 22.36, df = 16 (P = 0.13); I² = 28%						
Test for overall effect: Z = 1.35 (P = 0.18)						
Test for subgroup differences: Chi² = 1.41, df = 1 (P = 0.24), I² = 29.1%						

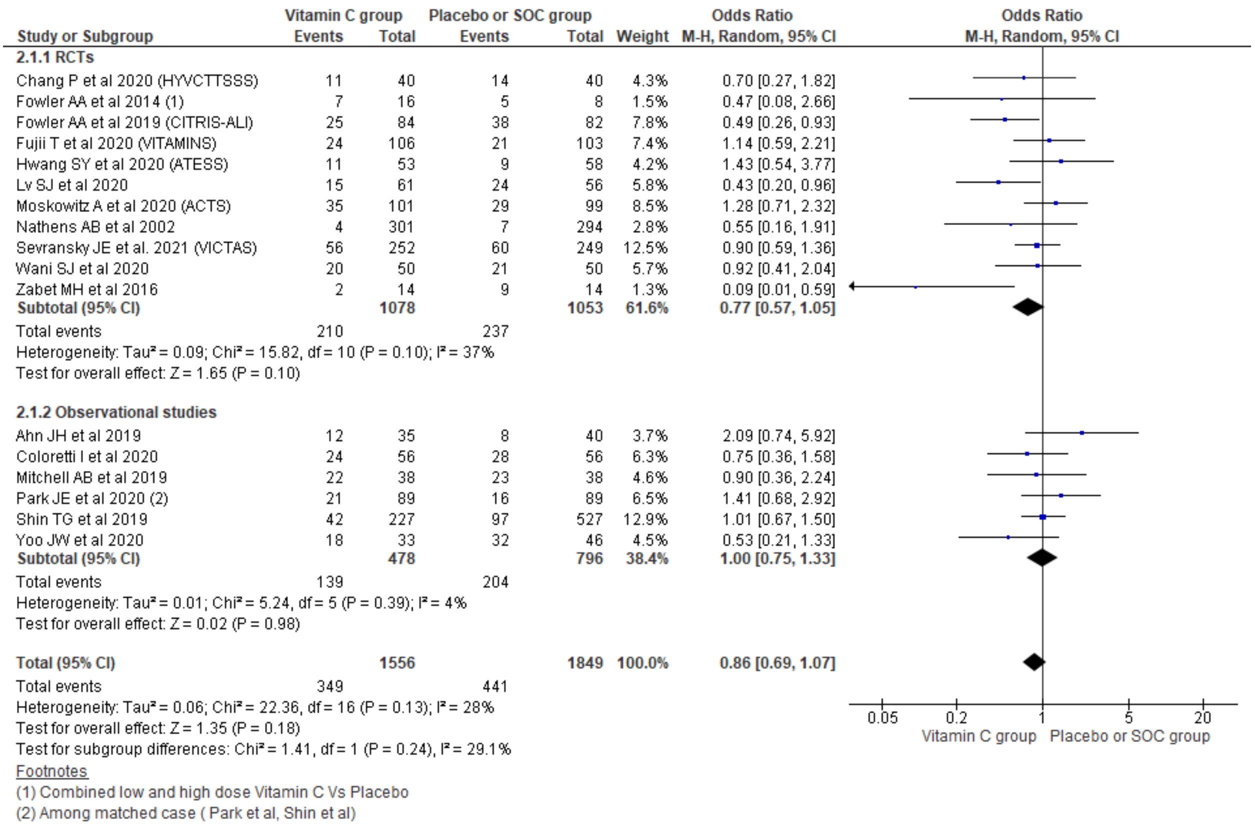

Footnotes
(1) Combined low and high dose Vitamin C Vs Placebo
(2) Among matched case (Park et al, Shin et al)

Figure S6. Forest plot showing 28/30-days mortality outcome using random effect model

3. ICU mortality

Pooling data from 14 studies reporting ICU mortality showed OR of 0.83 for overall ICU mortality (95% CI, 0.61-1.13; n= 2448; I^2 = 38%; p=0.24), though it could not reach statistical significance. Similarly, further subgroup analysis taking type of study under consideration showed similar result with RCTs (OR, 1.02; 95% CI, 0.75-1.38; n= 1716; I^2 = 0%; p=0.91) and observational studies (OR, 0.69; 95% CI, 0.41-1.17; n= 732; I^2 = 55%; p=0.17) (**Figure S7**).

Study or Subgroup	Vitamin C group Events	Vitamin C group Total	Placebo or SOC group Events	Placebo or SOC group Total	Weight	Odds Ratio M-H, Random, 95% CI
**3.1.1 RCTs**						
Fujii T et al 2020 (VITAMINS)	21	107	19	104	10.2%	1.09 [0.55, 2.18]
Hwang SY et al 2020 (ATESS)	7	46	7	52	5.5%	1.15 [0.37, 3.58]
Iglesias J et al 2020 (ORANGES)	6	68	10	69	5.9%	0.57 [0.20, 1.67]
Nathens AB et al 2002	3	301	9	294	4.4%	0.32 [0.09, 1.19]
Palli E et al 2017	15	60	11	64	7.8%	1.61 [0.67, 3.85]
Sevransky JE et al. 2021 (VICTAS)	52	252	49	249	14.5%	1.06 [0.69, 1.64]
Yanase F et al 2019 (VICCSS)	1	25	0	25	0.9%	3.12 [0.12, 80.39]
**Subtotal (95% CI)**		**859**		**857**	**49.1%**	**1.02 [0.75, 1.38]**
Total events	105		105			
Heterogeneity: Tau² = 0.00; Chi² = 5.73, df = 6 (P = 0.45); I² = 0%						
Test for overall effect: Z = 0.11 (P = 0.91)						
**3.1.2 Observational studies**						
Ahn JH et al 2019	12	35	12	40	6.8%	1.22 [0.46, 3.22]
Chang K et al 2020	16	52	20	88	9.0%	1.51 [0.70, 3.27]
Litwak JJ et al 2019	17	47	18	47	8.2%	0.91 [0.40, 2.11]
Long MT et al 2020	9	79	33	127	8.7%	0.37 [0.16, 0.81]
Mitchell AB et al 2019	12	38	13	38	6.9%	0.89 [0.34, 2.31]
Sadaka F et al 2019	3	31	13	31	4.0%	0.15 [0.04, 0.59]
Yoo JW et al 2020	18	33	32	46	7.2%	0.53 [0.21, 1.33]
**Subtotal (95% CI)**		**315**		**417**	**50.9%**	**0.69 [0.41, 1.17]**
Total events	87		141			
Heterogeneity: Tau² = 0.27; Chi² = 13.37, df = 6 (P = 0.04); I² = 55%						
Test for overall effect: Z = 1.38 (P = 0.17)						
**Total (95% CI)**		**1174**		**1274**	**100.0%**	**0.83 [0.61, 1.13]**
Total events	192		246			
Heterogeneity: Tau² = 0.12; Chi² = 21.13, df = 13 (P = 0.07); I² = 38%						
Test for overall effect: Z = 1.17 (P = 0.24)						
Test for subgroup differences: Chi² = 1.56, df = 1 (P = 0.21), I² = 35.9%						

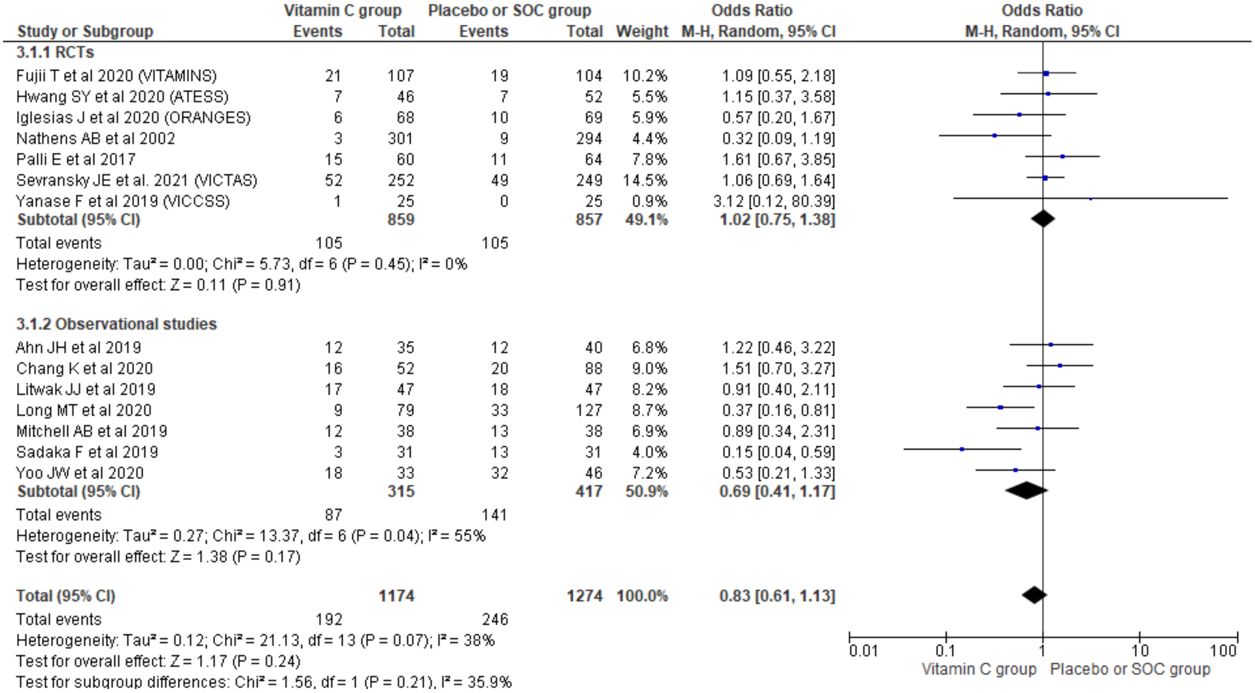

Figure S7. Forest plot showing ICU mortality outcome using random effect model

4. Overall length of hospital stay (LoHS)

4.1. Among RCTs

a. Based on patient group:

Among patients in sepsis/septic shock there was no significant differences in length of hospital stay between two groups (MD, 0.63; 95% CI, -0.41 to 1.68; n= 1141; I^2 = 0%; p=0.23) however among critically ill surgical patients average 1.7 days reduction in LoHS was observed among vitamin C group comparing with placebo/SOC (MD, -1.70; 95% CI, -2.89 to -0.51; n= 663; I^2 = 52%; p=0.005) (**Figure S8**).

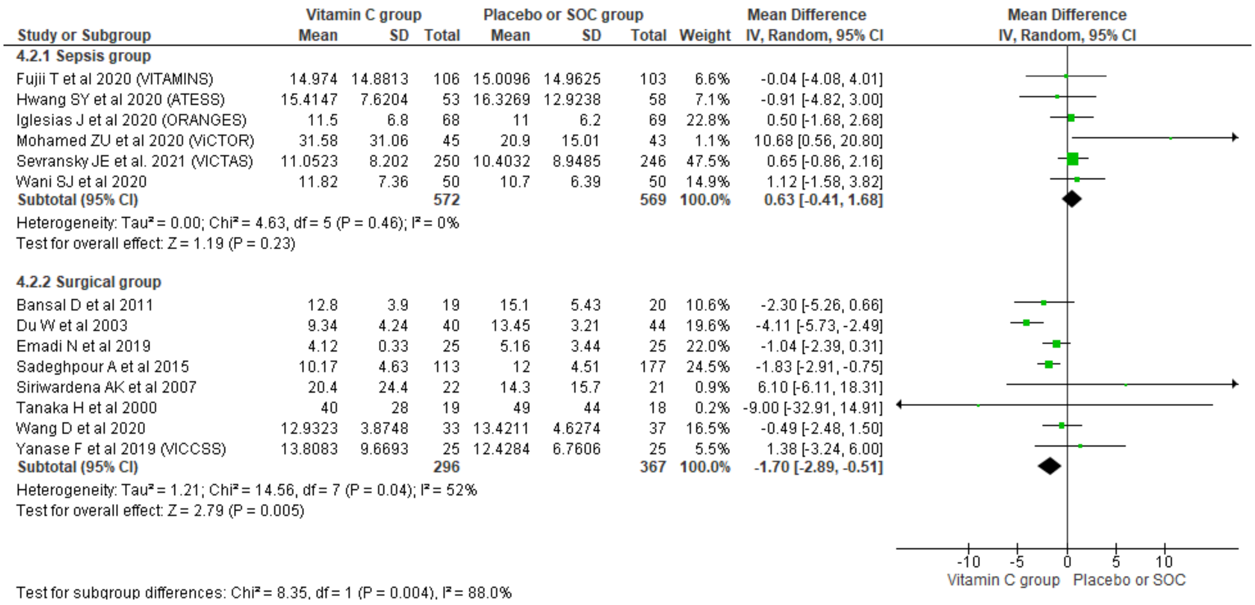

Figure S8. Forest plot showing LoHS outcome across surgical and sepsis group among RCTs

b. **Based on blinding**

Running analysis for LoHS taking RCTs based on blinding of trials showed some reduction in length of hospital stay of vitamin C group among both double blinded trials (MD, -0.54; 95% CI, -1.43 to 0.36; n= 1247; $I^2 = 34\%$; p=0.24) and single blinded/open RCTs reduction in LoHS among vitamin C group (MD, -0.78; 95% CI, -3.77 to 2.20; n= 557; $I^2 = 73\%$; p=0.61) comparing with placebo or SOC group but could not reach statistical significance (**Figure S9**).

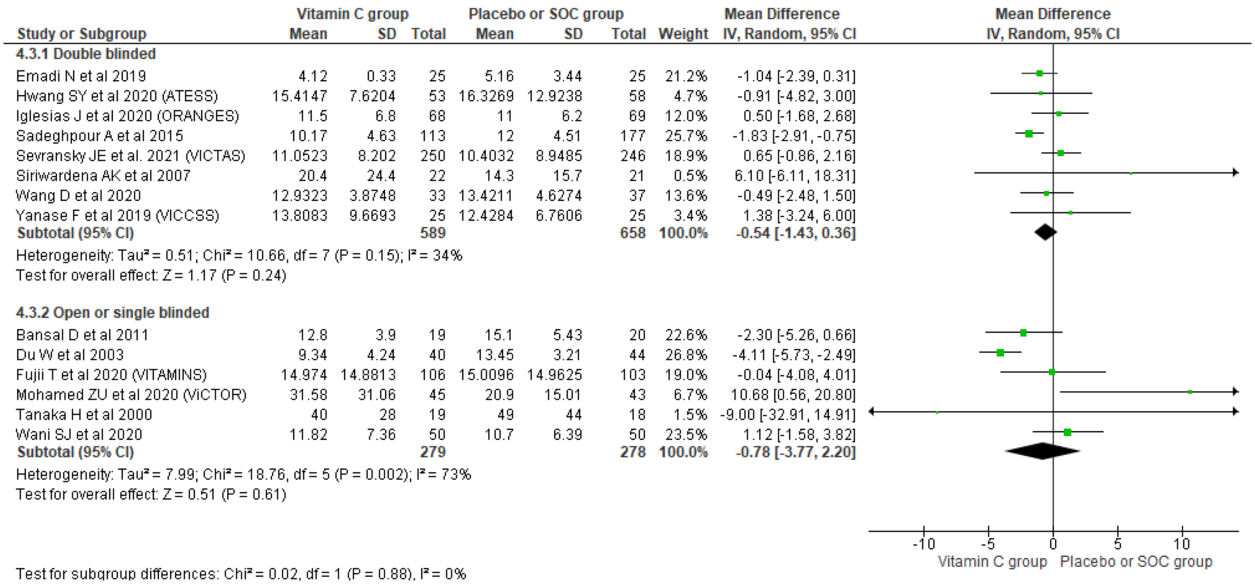

Figure S9. Forest plot showing LoHS outcome across double blinded and single blinded/open RCTs

c. Based on concomitant other anti-oxidants use

Analysis comparing vitamin C only with placebo/ SOC showed on an average 1.7 days reduction in LoHS among vitamin C group (MD, -1.70; 95% CI, -3.02 to -0.37; n= 581; I^2 = 61%; p=0.01) while such reduction is not seen in other concomitant anti-oxidants use with vitamin C (MD, 0.31; 95% CI, -0.91 to 1.53; n= 1223; I^2 = 21%; p=0.62) also could not show significant differences in hospital mortality (**Figure S10**).

Study or Subgroup	Vitamin C group Mean	SD	Total	Placebo or SOC group Mean	SD	Total	Weight	Mean Difference IV, Random, 95% CI
**4.4.1 Vitamin C only**								
Du W et al 2003	9.34	4.24	40	13.45	3.21	44	22.2%	-4.11 [-5.73, -2.49]
Emadi N et al 2019	4.12	0.33	25	5.16	3.44	25	24.7%	-1.04 [-2.39, 0.31]
Sadeghpour A et al 2015	10.17	4.63	113	12	4.51	177	27.2%	-1.83 [-2.91, -0.75]
Tanaka H et al 2000	40	28	19	49	44	18	0.3%	-9.00 [-32.91, 14.91]
Wang D et al 2020	12.9323	3.8748	33	13.4211	4.6274	37	19.0%	-0.49 [-2.48, 1.50]
Yanase F et al 2019 (VICCSS)	13.8083	9.6693	25	12.4284	6.7606	25	6.6%	1.38 [-3.24, 6.00]
**Subtotal (95% CI)**			**255**			**326**	**100.0%**	**-1.70 [-3.02, -0.37]**
Heterogeneity: Tau² = 1.37; Chi² = 12.84, df = 5 (P = 0.02); I² = 61%								
Test for overall effect: Z = 2.51 (P = 0.01)								
**4.4.2 Additional vitamins and anti-oxidants**								
Bansal D et al 2011	12.8	3.9	19	15.1	5.43	20	13.3%	-2.30 [-5.26, 0.66]
Fujii T et al 2020 (VITAMINS)	14.974	14.8813	106	15.0096	14.9625	103	7.9%	-0.04 [-4.08, 4.01]
Hwang SY et al 2020 (ATESS)	15.4147	7.6204	53	16.3269	12.9238	58	8.4%	-0.91 [-4.82, 3.00]
Iglesias J et al 2020 (ORANGES)	11.5	6.8	68	11	6.2	69	20.8%	0.50 [-1.68, 2.68]
Mohamed ZU et al 2020 (ViCTOR)	31.58	31.06	45	20.9	15.01	43	1.4%	10.68 [0.56, 20.80]
Sevransky JE et al. 2021 (VICTAS)	11.0523	8.202	250	10.4032	8.9485	246	31.8%	0.65 [-0.86, 2.16]
Siriwardena AK et al 2007	20.4	24.4	22	14.3	15.7	21	1.0%	6.10 [-6.11, 18.31]
Wani SJ et al 2020	11.82	7.36	50	10.7	6.39	50	15.3%	1.12 [-1.58, 3.82]
**Subtotal (95% CI)**			**613**			**610**	**100.0%**	**0.31 [-0.91, 1.53]**
Heterogeneity: Tau² = 0.62; Chi² = 8.86, df = 7 (P = 0.26); I² = 21%								
Test for overall effect: Z = 0.49 (P = 0.62)								
Test for subgroup differences: Chi² = 4.76, df = 1 (P = 0.03), I² = 79.0%								

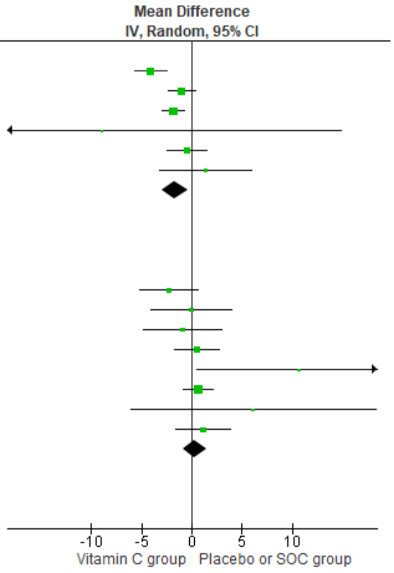

Figure S10. Forest plot showing hospital mortality outcome across use of vitamin C only or other anti-oxidants with vitamin C as treatment group across RCTs

4.2. Among observational studies

a. Based on patient group:

In both sepsis/septic shock group (MD, 1.34; 95% CI, -0.43 to 3.10; n = 246631; I2 = 64%; p=0.14) and surgical group (MD, -5.25; 95% CI, -14.19 to 3.69; n = 4526; I^2 = 97%; p=0.25) reduction in LoHS could not reach statistical significance comparing vitamin C with placebo/ SOC groups (**Figure S11**).

Study or Subgroup	Vitamin C group Mean	SD	Total	Placebo or SOC group Mean	SD	Total	Weight	Mean Difference IV, Random, 95% CI
**4.5.1 Sepsis group**								
Ahn JH et al 2019	24.7779	22.4164	35	41	33.8329	40	1.8%	-16.22 [-29.07, -3.37]
Litwak JJ et al 2019	17.9376	13.0005	47	15.0624	11.471	47	9.6%	2.88 [-2.08, 7.83]
Park JE et al 2020 (1)	17.0566	14.3178	89	16.1697	15.8249	89	11.3%	0.89 [-3.55, 5.32]
Sadaka F et al 2019	15.7126	9.3262	31	10.9389	12.2796	31	8.3%	4.77 [-0.65, 10.20]
Shin TG et al 2019	15.0526	9.6985	229	14.7511	11.1373	915	30.9%	0.30 [-1.15, 1.75]
Vail EA et al 2020 (2)	13.0503	9.6436	2115	11.05	8.1544	242963	38.2%	2.00 [1.59, 2.41]
**Subtotal (95% CI)**			**2546**			**244085**	**100.0%**	**1.34 [-0.43, 3.10]**
Heterogeneity: Tau² = 2.07; Chi² = 13.95, df = 5 (P = 0.02); I² = 64%								
Test for overall effect: Z = 1.49 (P = 0.14)								
**4.5.2 Surgical group**								
Dingchao H et al 1994	22	4	45	35	5.9	40	31.4%	-13.00 [-15.17, -10.83]
Giladi AM et al 2011	3.3501	3.7089	2272	4.7002	4.451	2022	32.0%	-1.35 [-1.60, -1.10]
Lin J et al 2018	35.0372	30.0463	38	28.4011	24.5627	42	20.2%	6.64 [-5.47, 18.74]
Sandesc M et al 2018	26.03	20.47	35	38.68	40.17	32	16.3%	-12.65 [-28.13, 2.83]
**Subtotal (95% CI)**			**2390**			**2136**	**100.0%**	**-5.25 [-14.19, 3.69]**
Heterogeneity: Tau² = 64.95; Chi² = 113.03, df = 3 (P < 0.00001); I² = 97%								
Test for overall effect: Z = 1.15 (P = 0.25)								

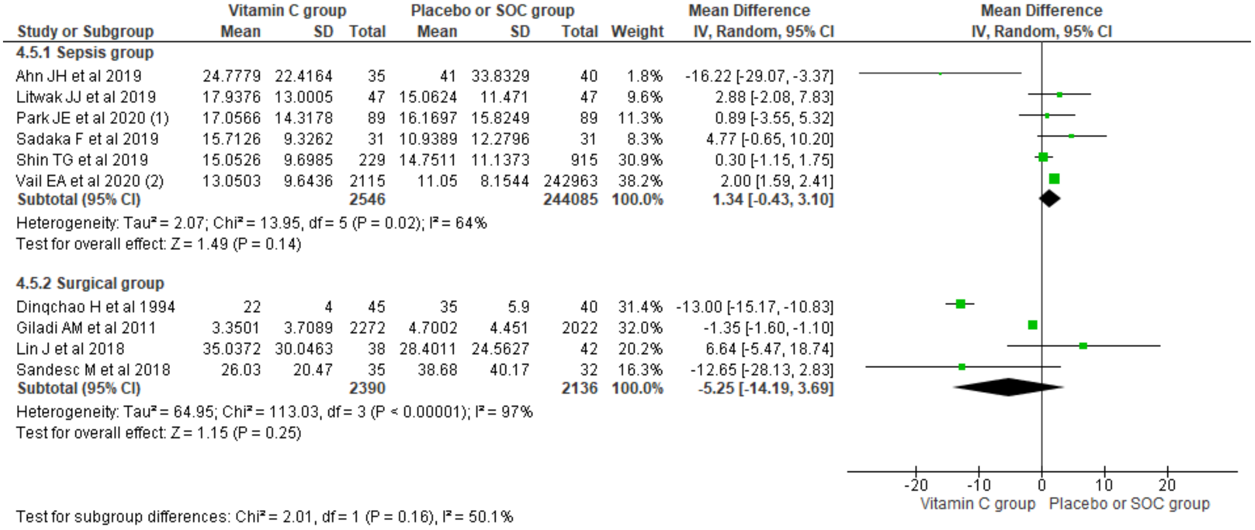

Test for subgroup differences: Chi² = 2.01, df = 1 (P = 0.16), I² = 50.1%

Footnotes
(1) Among matched cohort
(2) Among survivors

Figure S11. Forest plot showing hospital mortality outcome across surgical and sepsis group among observational studies

b. Based on concomitant other anti-oxidants use

Running analysis considering concomitant use of other anti-oxidants with vitamin C among observational studies also could not show significant differences in length of hospital stay comparing placebo/ SOC with vitamin C only (MD, -8.12; 95% CI, -19.97 to 3.74; n = 240; I^2 = 80%; p=0.18) or concomitant use of other anti-oxidants with vitamin C (MD, 0.83; 95% CI, -1.31 to 2.97; n= 250917; I^2 = 97%; p=0.45) (**Figure S12**).

Study or Subgroup	Vitamin C group Mean	SD	Total	Placebo or SOC group Mean	SD	Total	Weight	Mean Difference IV, Random, 95% CI
**4.6.1 Vitamin C only**								
Ahn JH et al 2019	24.7779	22.4164	35	41	33.8329	40	28.4%	-16.22 [-29.07, -3.37]
Dinqchao H et al 1994	22	4	45	35	5.9	40	42.0%	-13.00 [-15.17, -10.83]
Lin J et al 2018	35.0372	30.0463	38	28.4011	24.5627	42	29.5%	6.64 [-5.47, 18.74]
**Subtotal (95% CI)**			**118**			**122**	**100.0%**	**-8.12 [-19.97, 3.74]**
Heterogeneity: $Tau^2 = 85.83$; $Chi^2 = 10.13$, df = 2 (P = 0.006); $I^2 = 80\%$								
Test for overall effect: Z = 1.34 (P = 0.18)								
**4.6.2 Additional vitamins and anti-oxidants**								
Giladi AM et al 2011	3.3501	3.7089	2272	4.7002	4.451	2022	23.1%	-1.35 [-1.60, -1.10]
Litwak JJ et al 2019	17.9376	13.0005	47	15.0624	11.471	47	10.3%	2.88 [-2.08, 7.83]
Park JE et al 2020 (1)	17.0566	14.3178	89	16.1697	15.8249	89	11.6%	0.89 [-3.55, 5.32]
Sadaka F et al 2019	15.7126	9.3262	31	10.9389	12.2796	31	9.3%	4.77 [-0.65, 10.20]
Sandesc M et al 2018	26.03	20.47	35	38.68	40.17	32	1.8%	-12.65 [-28.13, 2.83]
Shin TG et al 2019	15.0526	9.6985	229	14.7511	11.1373	915	20.9%	0.30 [-1.15, 1.75]
Vail EA et al 2020 (2)	13.0503	9.6436	2115	11.05	8.1544	242963	23.0%	2.00 [1.59, 2.41]
**Subtotal (95% CI)**			**4818**			**246099**	**100.0%**	**0.83 [-1.31, 2.97]**
Heterogeneity: $Tau^2 = 5.14$; $Chi^2 = 195.84$, df = 6 (P < 0.00001); $I^2 = 97\%$								
Test for overall effect: Z = 0.76 (P = 0.45)								

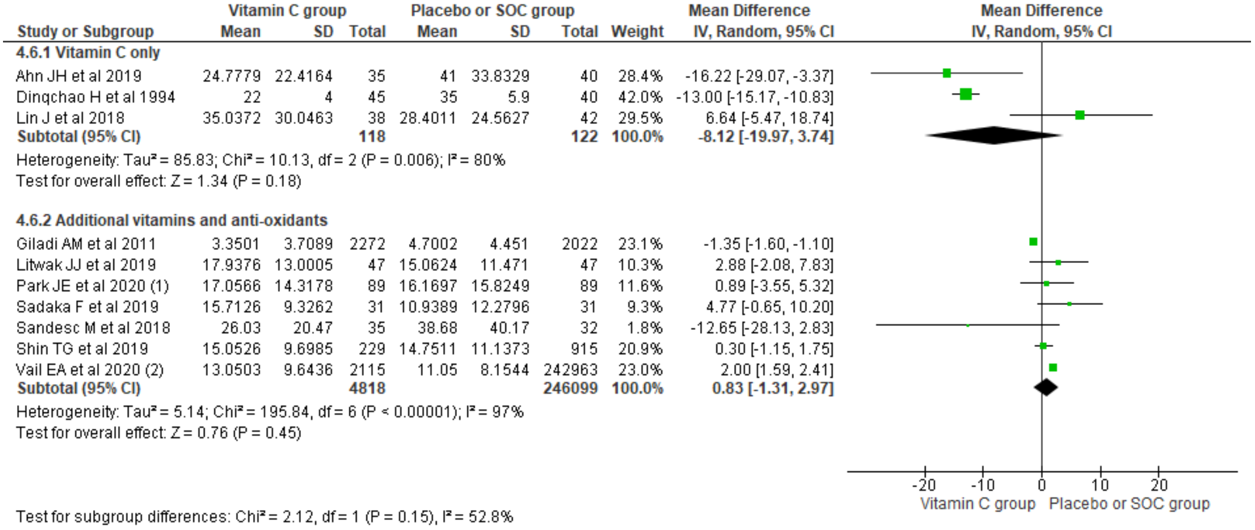

Test for subgroup differences: $Chi^2 = 2.12$, df = 1 (P = 0.15), $I^2 = 52.8\%$

Footnotes
(1) Among matched cohort
(2) Among survivors

Figure S12. Forest plot showing hospital mortality outcome across use of vitamin C only or other anti-oxidants with vitamin C as treatment group across observational studies

5. New AKI

Pooling new onset AKI outcome using random effect model also showed no significant difference in new AKI outcome (OR, 1.22; 95% CI, 0.94 to 1.58; n= 5330; $I^2 = 0\%$; p=0.14). Similarly, based on type of study also could not show significant changes with fixed effect model result among observational studies (OR, 1.66; 95% CI, 0.78 to 3.54; n= 4482; $I^2 = 37\%$; p=0.35) and RCTs (OR, 1.17; 95% CI, 0.85 to 1.61; n= 848; $I^2 = 0\%$; p=0.35) (**Figure S13**).

Study or Subgroup	Vitamin C group Events	Vitamin C group Total	Placebo or SOC Events	Placebo or SOC Total	Weight	Odds Ratio M-H, Random, 95% CI
**6.1.1 RCTs**						
Bansal D et al 2011 (1)	4	19	4	20	2.8%	1.07 [0.23, 5.05]
Chang P et al 2020 (HYVCTTSSS)	1	40	2	40	1.2%	0.49 [0.04, 5.60]
Fujii T et al 2020 (VITAMINS) (2)	75	107	75	104	19.3%	0.91 [0.50, 1.64]
Iglesias J et al 2020 (ORANGES)	54	68	52	69	10.6%	1.26 [0.56, 2.82]
Mohamed ZU et al 2020 (ViCTOR)	7	45	5	43	4.5%	1.40 [0.41, 4.80]
Moskowitz A et al 2020 (ACTS)	32	101	27	99	18.5%	1.24 [0.67, 2.27]
Siriwardena AK et al 2007 (3)	11	22	7	21	4.5%	2.00 [0.58, 6.87]
Yanase F et al 2019 (VICCSS)	14	25	12	25	5.5%	1.38 [0.45, 4.20]
**Subtotal (95% CI)**		**427**		**421**	**67.0%**	**1.17 [0.85, 1.61]**
Total events	198		184			
Heterogeneity: Tau² = 0.00; Chi² = 2.17, df = 7 (P = 0.95); I² = 0%						
Test for overall effect: Z = 0.94 (P = 0.35)						
**6.1.2 Observational studies**						
Ahn JH et al 2019	7	35	3	40	3.3%	3.08 [0.73, 13.00]
Giladi AM et al 2011	30	2272	26	2022	24.6%	1.03 [0.61, 1.74]
Kahn SA et al 2011	2	17	2	16	1.6%	0.93 [0.12, 7.55]
Lin J et al 2018	9	38	3	42	3.5%	4.03 [1.00, 16.23]
**Subtotal (95% CI)**		**2362**		**2120**	**33.0%**	**1.66 [0.78, 3.54]**
Total events	48		34			
Heterogeneity: Tau² = 0.23; Chi² = 4.79, df = 3 (P = 0.19); I² = 37%						
Test for overall effect: Z = 1.31 (P = 0.19)						
**Total (95% CI)**		**2789**		**2541**	**100.0%**	**1.22 [0.94, 1.58]**
Total events	246		218			
Heterogeneity: Tau² = 0.00; Chi² = 7.15, df = 11 (P = 0.79); I² = 0%						
Test for overall effect: Z = 1.46 (P = 0.14)						
Test for subgroup differences: Chi² = 0.72, df = 1 (P = 0.40), I² = 0%						

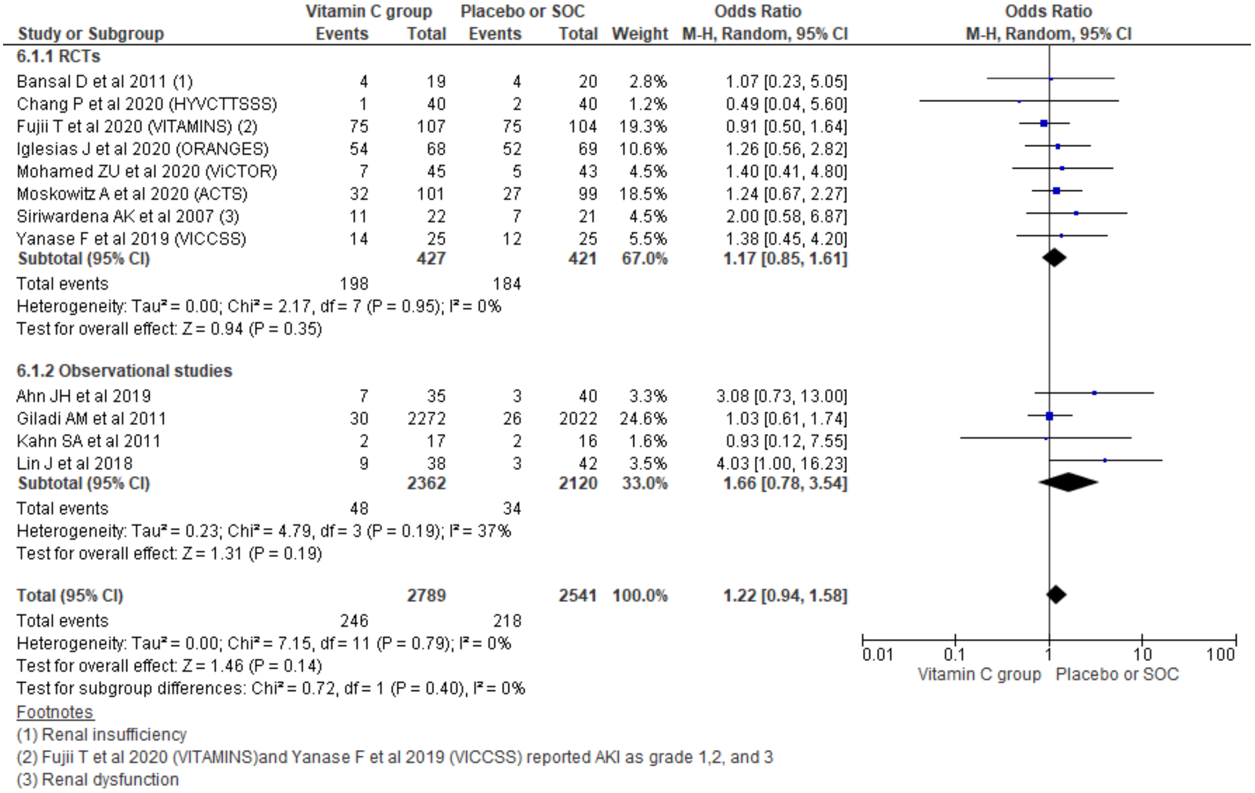

Footnotes
(1) Renal insufficiency
(2) Fujii T et al 2020 (VITAMINS)and Yanase F et al 2019 (VICCSS) reported AKI as grade 1,2, and 3
(3) Renal dysfunction

Figure S13. Forest plot showing new AKI outcome using random effect model
